# Genome-Wide Identification of ERF Transcription Factor Family and Functional Analysis of the Drought Stress-Responsive Genes in *Melilotus albus*

**DOI:** 10.3390/ijms231912023

**Published:** 2022-10-10

**Authors:** Na Wei, Qingyan Zhai, Hang Li, Shuwen Zheng, Jiyu Zhang, Wenxian Liu

**Affiliations:** State Key Laboratory of Herbage Improvement and Grassland Agro-Ecosystems, Key Laboratory of Grassland Livestock Industry Innovation, Ministry of Agriculture and Rural Affairs, Western China Technology Innovation Center for Grassland Industry, Engineering Research Center of Grassland Industry, Ministry of Education, College of Pastoral Agriculture Science and Technology, Lanzhou University, Lanzhou 730000, China

**Keywords:** *Melilotus albus*, ERF, drought stress, functional analysis, transgenic yeast

## Abstract

As an important forage legume with high values in feed and medicine, *Melilotus albus* has been widely cultivated. The AP2/ERF transcription factor has been shown to play an important regulatory role in plant drought resistance, but it has not been reported in the legume forage crop *M. albus*. To digger the genes of *M. albus* in response to drought stress, we identified and analyzed the ERF gene family of *M. albus* at the genome-wide level. A total of 100 *MaERF* genes containing a single AP2 domain sequence were identified in this study, named *MaERF001* to *MaERF100*, and bioinformatics analysis was performed. Collinearity analysis indicated that segmental duplication may play a key role in the expansion of the *M. albus* ERF gene family. *Cis*-acting element predictions suggest that *MaERF* genes are involved in various hormonal responses and abiotic stresses. The expression patterns indicated that *MaERFs* responded to drought stress to varying degrees. Furthermore, four up-regulated *ERFs* (*MaERF008*, *MaERF037*, *MaERF054* and *MaERF058*) under drought stress were overexpressed in yeast and indicated their biological functions to confer the tolerance to drought. This work will advance the understanding of the molecular mechanisms underlying the drought response in *M. albus*. Further study of the promising potential candidate genes identified in this study will provide a valuable resource as the next step in functional genomics studies and improve the possibility of improving drought tolerance in *M. albus* by transgenic approaches.

## 1. Introduction

Periodic drought is the primary limitation on plant growth and yield of crops in agricultural systems [1]. Drought-induced loss in crop yield probably exceeds losses from all other causes, since both the severity and duration of the drought stress are critical [2]. Under drought conditions, plants can resist the negative effects of harsh environments to a certain extent by mobilizing stress response genes and developing a variety of defense mechanisms at the molecular and physiological levels [3]. Transcription factors play important roles as master regulators in various biological processes and are considered to be excellent candidate genes for improving crops by genetic engineering [4]. The AP2/ERF (APETA-LA2, Ethylene Response Factor) superfamily is one of the largest transcription factor families in plants, accounting for approximately 9% of the total number of known plant transcription factor genes [5]. The AP2/ERF family of genes contains the AP2/ERF domain, which consists of 60–70 amino acids [6]. According to the sequence similarity and the number of domains, AP2/ERF family transcription factors can be divided into four subfamilies: ABI3/VP1 (RAV), AP2, ERF and Soloist [7]. Among them, the AP2 subfamily contains two AP2 conserved domains that are involved in the regulation of flower, ovule and seed development and the ability to maintain meristems [8]. The ERF subfamily contains an AP2/ERF domain, which functions in response to plant biotic and abiotic stresses and includes the ERF subfamily and the DREB subfamily. The RAV subfamily includes an AP2/ERF domain and a B3 domain with conserved DNA-binding domains present in other plant-specific transcription factors (TFs) [9]. In the past few decades, ERF family genes have attracted attention because overexpressing *ERF* genes in transgenic plants can improve abiotic stress tolerance [10].

The ERF (Ethylene Response Factor) transcription factor was first known as the ethylene response element binding protein, and according to the type of amino acids, 14 and 19 in the AP2/ERF domain, the ERF transcription factor family can be further divided into the ERF subfamily and DREB subfamily [11]. The ERF subfamily members usually bind to the GCC-box with AGCGCCC as the core sequence in ethylene-responsive genes, while DREB binds to the DRE *cis*-acting element with A/GCCGAC as the core sequence [12,13]. However, in recent years, more and more research has shown that ERF and DREB could simultaneously bind to GCC-box and DRE *cis*-acting elements [14,15,16], suggesting that the ERF and DREB subfamilies may have similar functions in different developmental stages of plants. In the past few decades, ERF family genes have attracted attention because overexpression of *ERF* genes could improve various abiotic stress tolerance in transgenic plants [10,17]. In *Arabidopsis thaliana*, it was reported that the *AtERF1* gene responds to drought and high salt stress by combining with GCC-box and DRE elements, and overexpression of *AtERF1* enhanced the tolerance to drought and salt in *Arabidopsis* [18]. In rice (*Oryza sativa*), overexpression of *OsERF48* could enhance root growth and drought tolerance by regulating *OsCMLI6*, a calmodulin-like protein [19]. Similar reports also found that in legume, *GmDREB2A* and *MtDREB2A* could activate the expression of downstream stress-related genes and significantly enhance the tolerance to drought in *soybean* (*Glycine max)* and *Medicago truncatula* [20,21]. In addition, it has recently been reported that ERF transcription factors can also induce the accumulation of proline. For example, the *OsERF71* gene in rice regulates the expression of proline synthesis genes under drought stress, resulting in the accumulation of proline. Under salt stress, overexpression *VaERF3* of *Vigna angularis* increases the level of proline accumulation and improves the salt resistance in *Arabidopsis* [22].

*M. albus* is an important forage crop worldwide, and it has high economic and utilization value for the promotion of green agriculture, rural areas and sustainable agricultural production. Mainly distributed in northwest, north, and northeast China, it has the characteristics of strong vitality, wide adaptability, fast reproduction, high seed yield, and strong nitrogen fixation ability [23]. In particular, the *M. albus* (2n = 16) genome and transcriptome were sequenced, and the first high-quality genome was recently published [24]. The publication of this genome provides information on *M. albus* genotypes and permits genome-wide research on this species. However, there have not been reports of ERF genes involved in drought stress resistance in *M. albus*. Based on these data, we identified the *MaERF* gene family at the genome-wide level, analyzed their expression patterns under drought stress, and verified the function of *MaERF* genes under drought stress through heterologous expression in yeast. The genome-wide study of the ERF gene family in *M. albus* can help us understand the molecular mechanisms of its stress resistance and provide valuable clues for the functional characterization of *MaERF* genes in response to drought stress, which will provide genetic resources with greater resistance to stress through transgenic technology.

## 2. Results

### 2.1. Gene Identification and Chromosomal Localization of MaERFs

**Figure 1:**
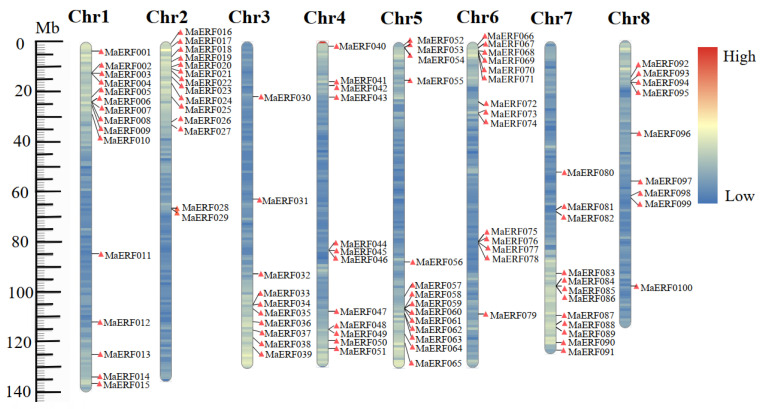
Chromosomal location distribution of 100 *MaERF* genes. The position of each gene shown on the graph is the average of the two ends of the gene, and the chromosome numbers are arranged in order. The scale on the left is in megabases (Mb). Chromosome colors from blue to red represent gene density from low to high gradually. The chromosome location map was generated using the R 3.6.3 software.

To identify ERF transcription factors in *M. albus*, previously identified ERF proteins in model plants were used as a query dataset for MaERF proteins in the *M. albus* genome. A total of 115 potential MaERF protein sequences were screened in the local database for *M. albus*. After deredundancy and domain identification, 100 MaERF protein sequences with one AP2 domain were retained for further phylogenetic and functional analysis (Table S1). All *MaERF* genes were named *MaERF001*–*MaERF100* according to their locations on the eight chromosomes (Figure 1). The results showed that the 100 *MaERF* genes were unevenly distributed across chromosomes, with the largest number of genes on Chr1 and the smallest number of genes on Chr8.

### 2.2. Phylogenetic Evolution and Physicochemical Properties Analysis of MaERFs

**Figure 2:**
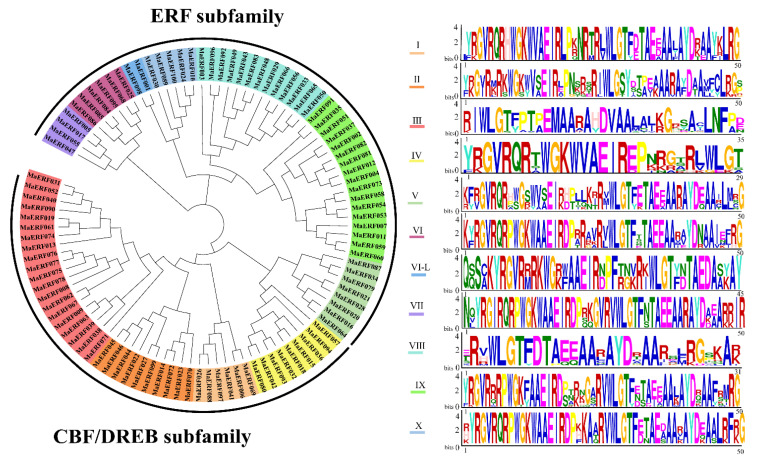
Phylogenetic relationship of 100 MaERF proteins in *M. albus*. An unrooted phylogenetic tree was constructed based on the multiple alignments of 100 *MaERFs* amino acid sequences with 1000 directed repeats. The classification of each subgroup is represented by a colored box. According to the classification method of *Arabidopsis*, 100 *MaERF* genes are divided into the ERF subfamily and DREB subfamily with 10 groups. Sequence-conserved motifs that are genetically similar in each group are identified by MEME.

To classify and study the evolutionary relationship of the ERF gene family in *M. albus*, the *ERF* gene classification of the model plant *Arabidopsis* was selected as a reference. A total of 122 At*ERF* genes and 100 *MaERF* genes with a single AP2/ERF domain were assigned to the ERF family, and based on the similarity of their encoded amino acid sequences, these genes were further classified into two subfamilies, including DREB classes (subgroups I, II, III, and IV) and ERF classes (subgroups V, VI, VI-L, VII, VIII, IX and X), based on phylogenetic analysis (Figure 2 and Figure S1).

In addition, the physicochemical properties of the 100 *MaERF* genes were also analyzed in this experiment, including protein length, MW (Molecular weight), *p*I (isoelectric point), GRAVY (Grand average of hydropathicity), and subcellular localization prediction (Table 1). The length of the 100 MaERF proteins identified ranged from 141 (*MaERF037*) to 506 (*MaERF032*) amino acids (aa). The molecular weights ranged from 14, 834 (*MaERF002*) to 56,148 (*MaERF032*) Da, and the *p*I ranged from 4.62 (*MaERF039*) to 10.19 (*MaERF029*). The grand average hydropathicity of all MaERF proteins was negative (<0), which shows that these MaERF proteins have good hydrophilicity. Moreover, the results of subcellular localization prediction indicated that most of the genes in this family are predicted to be located in the nucleus, followed by the cytoplasm (Table S2).

### 2.3. Multiple Sequence Alignment and Motif Distribution and Gene Structure Analysis of MaERFs

**Figure 3:**
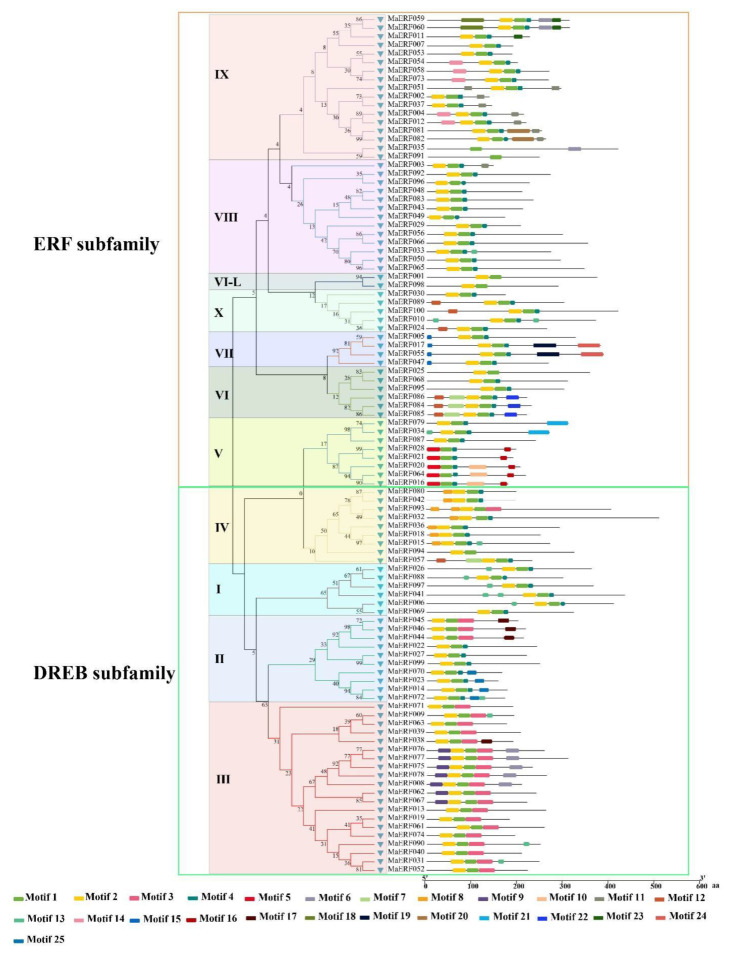
MEME was used to analyze protein motifs of MaERF proteins, the phylogenetic tree was constructed using MEGAX by the neighbor-joining method with 1000 bootstrap replicates.

**Figure 4:**
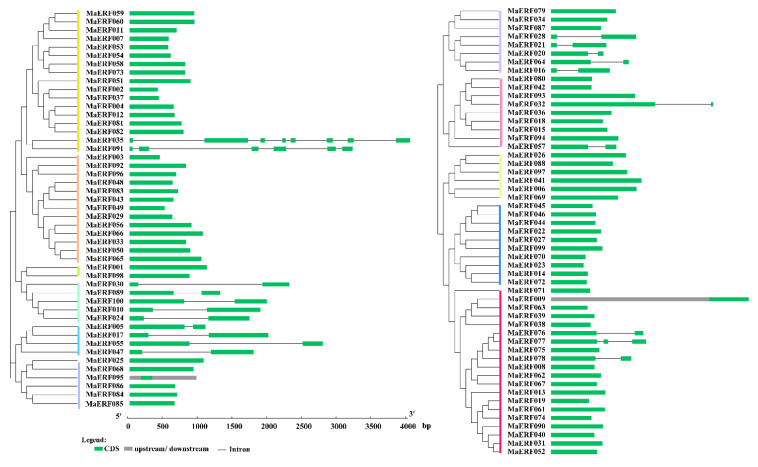
Structural analysis of the *MaERF* genes of *M. albus*. We clustered genes according to their phylogenetic relationships, with different colors representing different groups.

To study the sequence characteristics of ERF family genes, the MaERF protein sequences of *M. albus* were used for alignment. The results of multiple sequence alignment showed that the ERF gene family contains at least one highly conserved AP2/ERF DNA-binding domain, which consists of 57–70 amino acid residues. AP2 is characterized by two conserved regions called YRG and RAYD. Regarding the AP2 domain, the structure prediction revealed three β-sheets (β1, β2, and β3) and one α-helix region that shared significant amino acid similarity within two YRG and RAYD elements (Figure S2). In addition, the AP2 domains of ERF members were highly consistent among the groups; these domains are marked with red rectangles in the figure and include the β1-sheet G5 (glycine), β3-sheet G31 (glycine) and A39 (alanine) site in the α-helix. The conserved motifs in AP2/ERF family proteins in *M. albus* were investigated using MEME, revealing a total of 25 conserved motifs (designated motifs 1–25), as shown in Figure 3. Proteins in the same group contain similar motifs, while the motifs are divergent among different groups. For example, motif 6 is only present in the III group, motif 22 is only present in the VI group, and motif 16 is only present in the V and VII groups of the ERF subfamily. Similarly, motifs 1 and 2 are shared by members of the AP2 family. These results indicate that most motifs are distributed among specific groups, which is correlated with their functional divergence. Subsequently, we analyzed the gene structural characteristics of these 100 *MaERF* genes, and the results showed that 78% of genes in this family had no introns, and only 2% of them had UTR structural characteristics (Figure 4).

### 2.4. Duplication Events and Ka/Ks Analysis of the MaERFs

**Figure 5:**
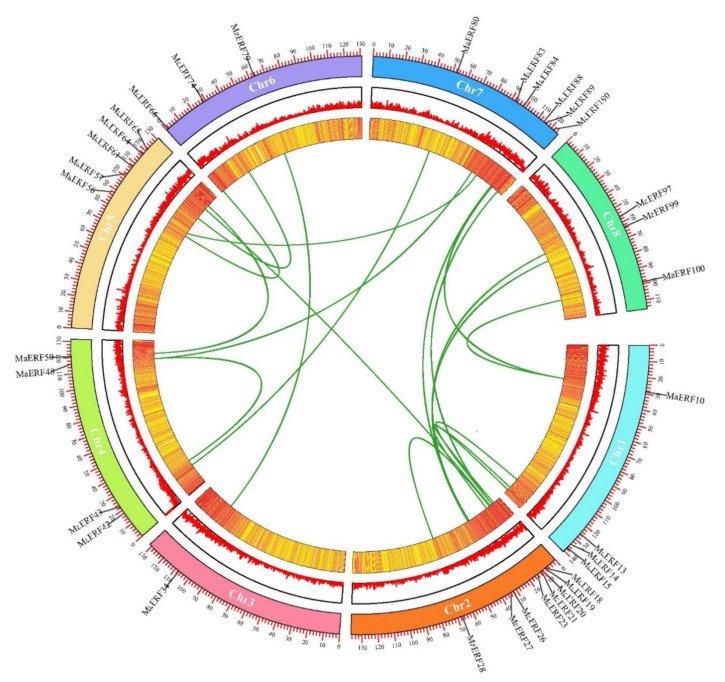
The collinearity analysis was performed on the *MaERF* genes, and only 18 pairs of gene names with collinearity were marked on the chromosome, and the green lines in the circle indicated the collinear relationship between genes.

To further examine the evolutionary relationship of *MaERF* genes, segmented and tandemly repeated genome duplication events were investigated by MCScanX collinearity analysis (Figure 5). The results showed that these 19 pairs of genes were all located on different chromosomes, indicating that chromosomal segment duplication was the main method for the expansion of the ERF gene family in *M. albus*.

In addition, the *Ka/Ks* ratio is widely used to measure the genetic evolution and selection pressure of genes. The *MaERF057*/*MaERF084* gene pair was too divergent due to gene sequence differences; thus, a numerical value could not be calculated. The *Ka/Ks* values of the remaining 18 gene pairs were all < 1, which indicated that purifying selection was the main force driving the evolution of the ERF gene family in *M. albus* (Table 2).

### 2.5. Expression Pattern Analysis in Response to Drought Stress of MaERFs

**Figure 6:**
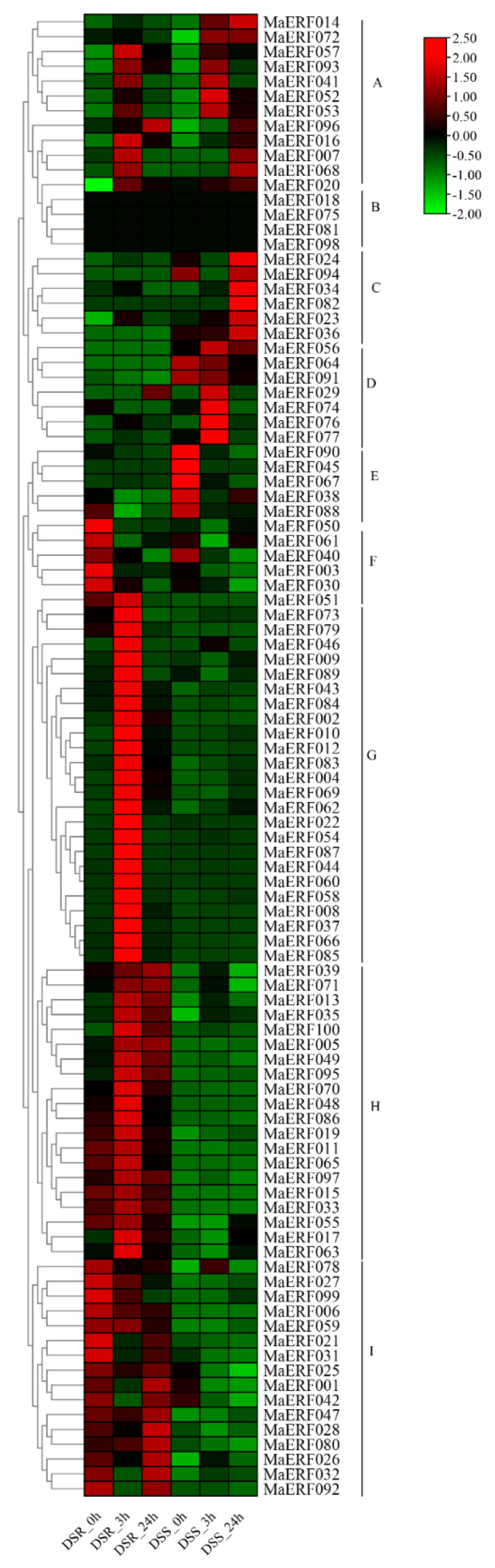
Heatmap representation of *MaERF* gene expression patterns under drought stress. Groups A to I exhibited nine expression patterns of *MaERF* genes. The bars at the right of the heatmap represent relative expression values. Root and shoot of drought stress expression levels at 0 h, 3 h and 24 h are shown.

To verify whether *MaERF* genes are involved in response to drought stress, we investigated the expression patterns of the 100 *MaERF* genes identified in this experiment under drought stress conditions. The expression profiles of 100 *MaERF* genes from two plant parts, namely, drought stress of shoot (DSS) and drought stress of root (DSR) were determined. According to the hierarchical clustering result, these *MaERF* genes had different transcription levels under various drought treatment time points, and could be clustered into nine clades, named A to I (Figure 6). Most genes showed significant up-regulation profiles under drought treatment in roots and peaked at 3 h, such as those in A, D, G and H groups, suggesting that these genes can respond rapidly to drought stress. Genes in Group C showed significant up-regulation after drought stress and peaked at 24 h in shoots, indicating that the genes in this group were positively regulated. The expression levels of group E and group F were suppressed after drought stress both in roots and shoots, and the expression levels of group B genes were basically kept unchanged in two tissues throughout the treatment.

### 2.6. qRT-PCR Validation of MaERF Genes Expression

**Figure 7:**
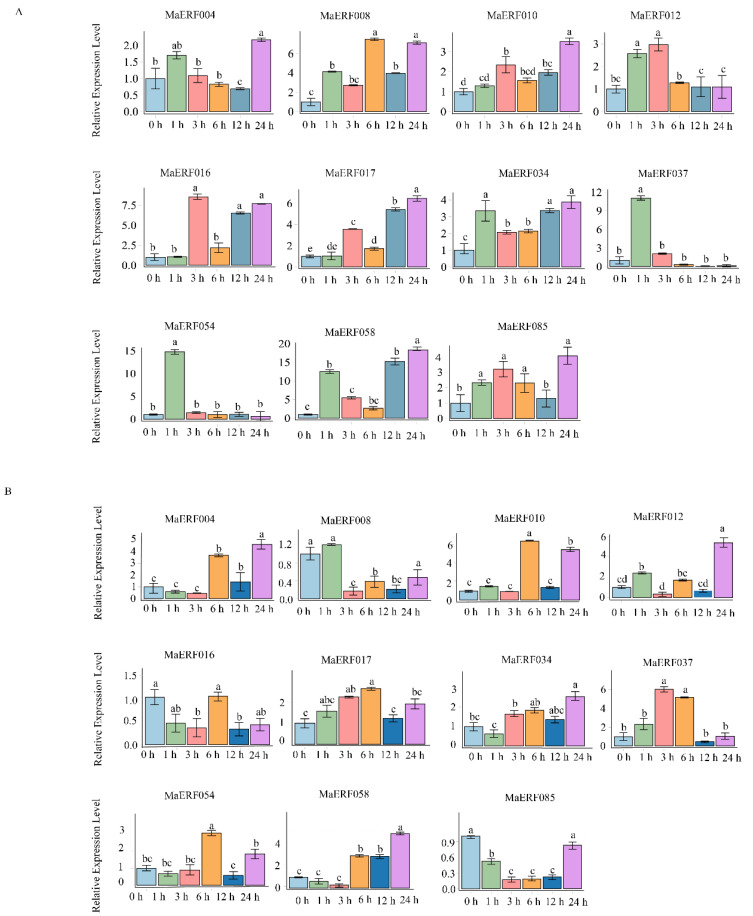
Expression patterns of *MaERF* genes in response to drought stress. The relative expression levels of the *MaERF* gene in *M. albus* root (**A**) and shoot (**B**) treated with PEG6000 (20%) were determined by qRT-PCR. Different letters indicate significant differences between different treatment time (*p* < 0.05). The name of the gene is written on the top of each bar diagram (error bars indicate the standard deviation from three replicates).

In order to verify the response of MaERF genes to drought stress, 11 genes (MaERF004, MaERF008, MaERF010, MaERF012, MaERF016, MaERF017, MaERF034, MaERF037, MaERF054, MaERF058, and MaERF085) which significantly up-regulated (more than 10-fold) under drought stress were selected and further tested by qRT-PCR (Figure 7). These 11 genes showed different expression profiles under drought stress in different tissues. In roots, almost all MaERF genes induced rapidly under drought stress within 3 h, especially for MaERF037 and MaERF054, whose expression reached peaks at 1 h and expression levels were 10 times higher than that of control. Compared to root, most MaERF genes peaked at 6 h or 24 h under drought stress in shoot.

### 2.7. Analysis of cis-Acting Elements and Protein-Protein Interaction Networks of MaERFs

**Figure 8:**
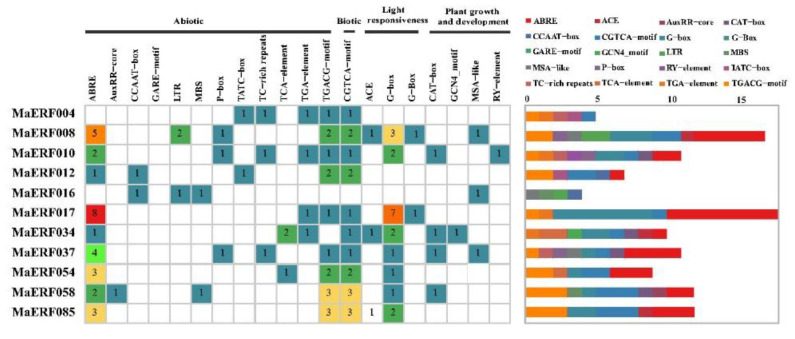
Analysis of *cis*-acting elements in the promoter in *MaERF* genes of the *M. albus*. A total of 20 *cis*-acting elements, including biotic, abiotic stress and phylogenetically related elements were counted, and their numbers were counted with different colors. The picture was generated using the R 3.6.3 software.

The promoter is a DNA sequence that determines the correct and efficient transcription of genes in plants. To study the function of *MaERF* promoters, the promoters of the 11 genes were extracted from 2000 bp upstream of the start codons using TBtools. Subsequently, potential functional elements in the promoter regions of these genes were analyzed by querying the PlantCARE database. The promoters are mainly grouped into four categories: abiotic, biotic, light responsiveness, and plant growth and development (Figure 8). We found that all 11 *MaERF* genes contain *cis*-acting elements related to abiotic stress, such as ABRE (abscisic acid response element); AuxRR-core (auxin response element); LTR (low-temperature response element); MBS (drought stress-related elements) and other abiotic stress-related elements. Except for *MaERF004* and *MaERF016*, the remaining genes contain an ABRE, and only *MaERF016* and *MaERF058* contain an MBS element. These binding sites are located upstream of coding sequences and can provide binding sites for transcription factors that respond to abiotic stresses. In addition, except for *MaERF016*, all other genes had biological stress-related elements, and the *MaERF017* gene contained the largest number of light-response-related *cis*-elements. At the same time, not all 11 genes had elements related to growth and development.

Furthermore, the STRING softer was used to determine the functional and physical relationships of 11 MaERF proteins predicted to be stress related through an *M. truncatula* association model (Figure S3). Using the model crop *M. truncatula* as the reference genome, the protein interaction prediction of these 11 orthologs of MaERF proteins was performed. The predicted result shows that an NAC-like transcription factor (AET04823) and a gene of unknown function (AES64073) interacted with *ERF1A* in *M. truncatula*. A previous study found that overexpression of the NAC-like transcription factor confers tolerance to drought and high salt stress through an ABA-independent signaling pathway [25]. Whether the interaction between *ERF1A* and NAC-like transcription factors can improve plant stress resistance remains to be verified, which provides a reference for predicting the potential regulatory roles of MaERF proteins in *M. albus*.

### 2.8. Transgenic Yeast to Analysis Drought Stresses

**Figure 9:**
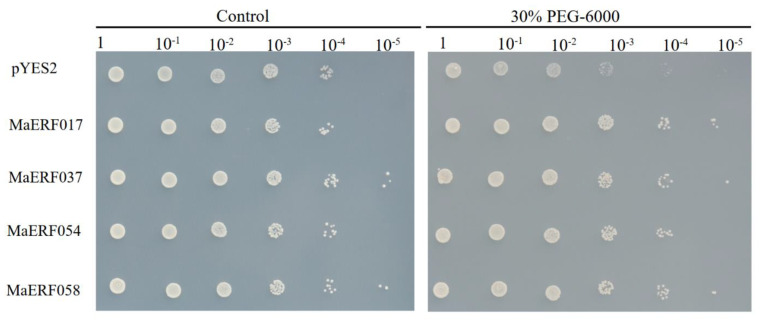
Expression of *MsERF017*, *MsERF037*, *MsERF054* and *MsERF058* in yeast-induced sensitivity to drought stress. The left panel represents the growth of yeast cells under normal conditions, and the right panel represents the growth of yeast cells under drought stress.

Considering both yeast and plants belong to eukaryotes, and yeast has been widely used as a model research system to study the functions of transcription factors under multiple abiotic stresses [10], in this study, we chose the yeast system to test the potential functions of four *MaERFs* in response to drought stress. The results showed that the transgenic yeast cells and untransformed empty yeast cells grew well under the control treatment. After simulating drought stress with 30% PEG-6000, the growth of transgenic yeast cells was significantly better than that of untransformed empty yeast cells (Figure 9). This indicated that heterologous expression of *MaERF017*, *MaERF037*, *MaERF054*, and *MaERF058* improved the tolerance of yeast to osmotic stress. It was preliminarily proven that these four drought-tolerant candidate genes were possibly involved in the drought-tolerant response of *M. albus*.

## 3. Discussion

AP2/ERF transcription factors are an important family of transcription factors in all plant species that can participate in the regulation of various growth and development processes in plants and the responses to various stresses [26]. At present, many studies have confirmed that the ERF family plays an important role in the response to plant abiotic stresses [11]. However, there have been no studies on the molecular function of ERF TFs in response to abiotic stresses in *M. albus*. Therefore, this study focused on systematically exploring the potential molecular functions of *MaERF* genes under drought stress.

First, we identified a total of 100 *MaERF* genes in *M. albus* (Figure S1). This result is similar to the number of genes in *M. truncatula* (n = 107) [27] and soybean (n=98) [28] but slightly less than that in rice (n = 139) and *Arabidopsis* (n = 122). In total, 100 ERF proteins were grouped into 11 different subfamilies through phylogenetic analysis. The phylogenetic analysis results of most of the ERF proteins were consistent with a previous report in *Arabidopsis* [29], which showed that the results of the phylogenetic analysis in the present study have a high degree of credibility (Figure S2). The nucleotide sequence lengths, molecular weights and theoretical isoelectric points of the *MaERF* genes varied. It is worth noting that all *MaERF* genes have good hydrophilicity, and most genes are predicted to be localized to the nucleus (Table S2), which is consistent with the fact that transcription factors regulate the transcription of downstream genes in the nucleus to adapt to changes in the external environment. In domain research, it was found that the *MaERF* domain contains two important regions, namely, YRG and RAYD (Figure S2). The YRG region contains 20 amino acids in the β1 sheet of the AP2 domain and has been shown to play a key role in establishing direct contact with DNA molecules. In contrast, the RAYD region contains approximately 40 amino acids in the α-helical region and is involved in protein‒protein interactions [30]. In addition, it has also been reported that the hydrophobic surface of the α-helix of the RAYD region interacts with the major groove of DNA [31]. Moreover, we found that gene members belonging to the same subfamily had similar motifs, and found that the motif structures of MaERF proteins in each group were highly conserved and all amino acid sequences of MaERF proteins contained a conserved AP2 motif (Figure 3). The above results are consistent with the reports of *Arabidopsis*, rice and soybean [29,32].

Gene structure analysis showed that the *MaERF* gene family had fewer introns (Figure 4). The number and distribution of introns may be linked to plant evolution, and the intron number of ERF subfamily genes has likely decreased during plant evolution [33,34], which was also confirmed in *Arabidopsis* and *M. truncatula* [27,29]. In addition, we found that gene duplication occurred in the *MaERF* gene family, and a total of 19 duplication events were identified. All repetitive genes show segmental duplication, indicating that segmental duplication is important to the process of gene duplication in the *MaERF* gene family. Meanwhile, *ERF* gene duplication has been found in many species, including *Rosa chinensis* [35] and *Gossypium barbadense* [36] (Figure 5). Interestingly, 18 of the *MaERF* genes had *Ka/Ks* ratios < 1, indicating that the *MaERF* gene family has undergone purifying rather than positive selection (Figure 6) [37].

A *cis*-regulatory element is a specific motif located in the promoter region of a gene that acts as a binding site for a gene and can play an important role in the stress response by regulating the transcription of downstream genes [38]. In addition, some phytohormones (abscisic acid, salicylic acid, jasmonic acid and ethylene) are also involved in regulating the adaptive response of plants to abiotic stresses [39]. After the analysis of *cis*-elements in the promoter region of the candidate genes screened in the previous stage [40], it was found that the promoter elements involved in stress response and hormone response, including ABRE, LTR, MBS and TC-rich elements, exist in the promoter region of *MaERF* genes. These elements are involved in the ABA response and low temperature, drought and other biotic or abiotic stress defense processes (Figure 7). The results indicate that the *MaERF* gene promoter may play an important role in the transcriptional activation of stress response-related genes and the process of the plant stress response [41].

Studies have shown that ERF transcription factors can be potential candidates for crop improvement as they are key regulators in different plant developmental processes and responses to various stresses [42]. Nevertheless, the function of the *MaERF* gene in *M. albus* is still unclear. Therefore, it is necessary to analyze the transcriptional regulation of ERF transcription factors in *M. albus*, in order to use them to improve the quality and abiotic stress tolerance of *M. albus*. Here, we first systematically analyzed the expression patterns of these genes under drought stress and determined the potential functions of *MaERF* genes under drought stress. We observed a significant up-regulated expression trend of *MaERF* genes under drought stress, implying that they may be broadly related to the regulation of *M. albus* in response to drought (Figure 8). In addition, the expression levels of *MaERF017*, *MaERF037*, *MaERF054*, and *MaERF058* genes were more fold-up-regulated, suggesting that these genes may play roles in key processes. In particular, *MaERF058* showed a continuous up-regulation with a 20-fold increase. Interestingly, *MtERF1A* (the homologous gene of *MaERF058* in *M. truncatula*) mediates resistance to a subset of root pathogens, playing an important function in *M. truncatula* responding to biotic stress [43]. As previously reported, overexpression of the *BrERF4* gene in *Arabidopsis* can improve drought and salt tolerance and overexpression of *SodERF3* in tobacco significantly improved the drought and salt tolerance of transgenic tobacco [41,44]. In this study, *MaERF017*, *MaERF037*, *MaERF054*, and *MaERF058* were validated in transgenic yeast cells, and the results showed that transgenic yeast cells grew better than empty vector yeast cells after drought resistance treatment (Figure 9). It is speculated that the drought resistance of transgenic yeast is related to the combination with the DRE/CRT (dehydration response element/C-repeat) *cis*-acting element (TAC-CGACAT) that can regulate the expression of a series of downstream stress response genes and functional proteins [42]. However, the specific reasons for this up-regulation of these genes are still to be verified, and whether these *MaERF* genes can function as negative regulators in drought stress in *M. albus* still needs to be further investigated. The results of this study will lay a foundation for analyzing the drought resistance function of the *MaERF* gene family in *M. albus* and creating new germplasm of *M. albus* with high drought resistance through genetic engineering technology.

## 4. Materials and Methods

### 4.1. Identification of the ERF Gene Family in M. albus

To identify *ERF* gene family members in *M. albus*, this study first obtained the ERF protein sequences of 122 *Arabidopsis*, 139 *O. sativa* and 98 *M. truncatula* proteins as a query [27,29]. The MaERF protein and gene sequences were obtained by searching the genome sequence of *M. albus* with the local BLASTP program, and the E value was 10^−5^ [45]. The Pfam program online tool (http://pfam.xfam.org/search#tabview=tab1, accessed on 9 November 2021) and the NCBI-CD Search (https://www. ncbi. nlm. nih. gov/Structure/cdd/wrpsb.Cgi, accessed on 12 November 2021) were used to determine that these sequences contained only one AP2 domain [46]. The CD-Hit tool (http://weizhongli-lab.org/cd-hit/, accessed on 12 November 2021) was used to remove redundant sequences in the remaining ERF protein sequences [40]. The molecular weight (MW), grand average hydropathicity (GRAVY), and isoelectric point (*p*I) of *M. albus* ERF gene family members were detected using ExPASy (http://web.expasy.org/protparam/), accessed on 18 November 2021 [47].

### 4.2. Chromosomal Location and Phylogenetic Analysis of MaERF Genes

Through the *M. albus* genomic gff3 data, the corresponding position of the *MaERF* gene on the chromosome was obtained. The chromosome location map was drawn using R 3.6.3 software. Then, we named the genes according to their position on the chromosome. Phylogenetic analysis of protein sequences was performed using MEGA-X software [48]. The phylogenetic tree was constructed based on the neighbor-joining (NJ) method, the bootstrapping multiple calculation method was set to 1000, and other parameters were set to the default [49].

### 4.3. AP2/ERF Domain Analysis of MaERF Genes

To analyze the sequence features of the AP2 domain in the MaERF protein, we performed a multiple sequence alignment of the ERF protein sequence in *M. albus* using DNAMAN [50]. The sequence logos for AP2 repeats were generated using WebLogo online tool (http://weblogo.threeplusone.com/, accessed on 12 December 2021) with the default settings [51].

### 4.4. Conserved Motif and Gene Structure Analysis of MaERF Genes

We performed conserved motif analysis using the online tool MEME Suit 5.3.3 (https://meme-suite.org/meme/tools/meme, accessed on 18 December 2021) [52]. The conserved motifs in full-length ERF proteins were identified using MEME, with the maximum number of motifs as 25 [32]. Gene structure analysis of the *ERF* gene family members in *M. albus* was performed using the GSDS 2.0 (gene structure display server) (http://gsds.gao-lab.Org/, accessed on 22 December 2021) online website [53].

### 4.5. Gene Duplication Events and Ka/Ks Analysis of MaERF Genes

We used the MCScanX tool to assess the complete genome sequences and genome annotation files of *M. albus* [54]. TBtools software was used to visualize the obtained results [55]. Advanced Circos was used to visualize the collinearity of homologous genes based on homology and their positions in the genome. In addition, nonsynonymous (*Ka*) and synonymous (*Ks*) substitution rates were calculated to explore the mechanism of gene divergence after duplication by using TBtools.

### 4.6. Expression Analysis of MaERF Genes under Drought Stresses

To analyze the expression profiles of the *MaERFs* in shoot stress (DSS) and the root stress (DRS) under drought stress, the expression data were obtained as reported previously [56], and used for heatmap creation to display the gene expression profiles with TBtools software.

### 4.7. Analysis of Cis-Acting Elements and Protein-Protein Interaction Networks of MaERF Genes

According to the results of the expression pattern analysis, the *cis*-acting elements of 11 *MaERF* genes with high differential expression were analyzed. Subsequently, TBtools was used to extract the promoter sequences 2000 bp upstream of the start codons of these genes. Next, potential *cis*-acting elements in the promoter regions of these genes were analyzed using the PlantCARE database (http://bioinformatics.psb.ugent.be/webtools/plantcare/html/, accessed on 29 December 2021) and graphs were drawn using R 3.6.3 software [57]. Using the model plant *M. truncatula* as a reference, the network structure interactions of 11 MaERF proteins were predicted, and the MaERF proteins network interactions were analyzed based on STRING software, where the high confidence was set to 0.4 and the max number was set to 10 [58].

### 4.8. Plant Cultivation and Drought Treatments

The variety “Ma46” of *M. albus* was chosen as the study material. First, we selected seeds with full grains and good shape for sterilization and germination. When the seeds germinated and revealed white buds, we selected seedlings with good growth and uniformity, transferred them to a hydroponic box containing 1/2 MS (pH = 5.8), and regularly replaced the culture medium in the hydroponic box [59]. The guaranteed incubation conditions were as follows: 16 h light/8 h dark cycle, 80% relative humidity, and 22 °C. Previous studies have indicated that H_2_O_2__,_ proline, malondialdehyde (MDA) and soluble sugar can be used as indicators to evaluate drought resistance in plants [60]. Our previous study found that under drought stress, the content of H_2_O_2_ peaked up to 24 h and the content of malondialdehyde (MDA), proline, and soluble sugar increased continuously up to 48 h, while the soluble protein content remained basically unchanged for 24 h and then decreased significantly [24]. So, in this study, we used 20% PEG6000 to simulate the drought stress and set different time points within 24 h [56]. To avoid the influence of circadian rhythm on plant growth and plant gene expression, the root and shoot tissue were uniformly sampled after 24 h, flash-frozen in liquid nitrogen and stored at −80 °C.

### 4.9. Plant RNA Extraction and Quantitative Real-Time PCR Analysis

Total RNA was extracted from *M. albus* under different treatment times. A cDNA synthesis kit (FastKing gDNA Dispelling RT SuperMix, Tiangen Biotech, Bei Jing) was used for reverse transcription, and first-strand cDNA was obtained after genomic DNA was removed. The concentration of cDNA was detected by a NanoDrop 2000 UV spectrophotometer (ND-8000, Xi’an) and then uniformly diluted to 100 ng·µL^−1^ for qRT‒PCR. Gene-specific primers for qRT‒PCR analysis were designed with NCBI Primer-BLAST (Table S3). qRT‒PCR analysis was performed using a 7500 fast real-time PCR system (Applied Biosystems, USA), and the experiment was performed in 3 technical replicates. The qRT‒PCR system included 5 µL 2SG Fast qPCR Master Mix (B639271-0005, Shanghai), 0.5 µL forward and reverse primers, 1 µL DNF Buffer, 1 µL cDNA and 2 µL ddH2O. The qRT‒PCR conditions were as follows: reaction denaturation (95 °C for 30 s), and 40 cycles of 95 °C for 5 s and 60 °C for 30 s. β-Tubulin was used as an internal reference gene for the ΔCt method to calculate the relative fold difference, and the gene expression level was calculated using the FC = 2^−ΔΔCt^ method [61].

### 4.10. Yeast Heterologous Expression and Functional Validation of MaERF Genes

Total RNA was extracted from the *M. albus* seedlings, and the first-strand cDNA was obtained after genomic DNA was removed. After the target gene was cloned and sequenced, the amplified fragment was ligated into the pYES2 (Invitrogen, Carlsbad, CA, USA) expression vector by seamless cloning; the primer sequences are shown in Table S4. An empty pYES2 control plasmid and five expression vectors were transformed into the yeast strain INVSc1 by using a lithium acetate procedure according to the pYES2 vector kit instructions [37]. Following a previous method [62], we performed the transformation of yeast strains, and the transformants were incubated in a synthetic complete (SC) medium devoid of uracil with 2% (*w*/*v*) glucose at 30 °C for 36 h at 220 rpm. The drought stress evaluation experiment was carried out in the SC-Ura medium. We added 30% PEG-6000 stress solution to the yeast cells and added water to another group as a blank control. Afterwards, we serially diluted the 36 h-stressed culture and spotted it on SC-Ura agar plates. The spotted SC-Ura medium was placed upside down at 30 °C and observed after 48 h.

## 5. Conclusions

This study explored the potential molecular function of *MaERF* genes in *M. albus*. We identified the *MaERF* gene family at the genome-wide level and studied the basic gene characteristics of the family. In addition, the gene structure, motif composition, phylogenetic relationship, chromosomal location, collinearity analysis, *cis*-regulatory elements and other characteristics of the ERF gene family were studied. At the same time, we studied the expression changes of this gene family under drought stress at the molecular level and verified the function of *MaERF* genes by heterologous expression in transgenic yeast. These results will enrich our knowledge of the *MaERF* gene family and lay a foundation for further exploration of the function of *MaERF* genes.

## Figures and Tables

**Table 1:** Analysis of physicochemical properties of each group in *MaERF* genes of *M. albus*.

ERF Groups	No of Genes	Length (aa)			Molecular Weight (Da)			*p*I			GRAVY		
		Max.	Min.	Avg.	Max.	Min.	Avg.	Max.	Min.	Avg.	Max.	Min.	Avg.
ERF-I	6	432	296	362	48,782	26,888	40,630	8.7	5.18	6.70	−0.50	−1.02	−0.67
ERF-II	10	245	155	199	26,888	17,822	22,134	8.73	4.67	6.26	−0.36	−1.05	−0.59
ERF-III	20	308	173	224	34,906	18,886	24,968	7.91	4.62	5.83	−0.31	−0.94	−0.62
ERF-IV	9	506	195	295	56,148	20,927	32,730	9.21	4.86	6.82	−0.44	−0.99	−0.75
ERF-V	8	309	176	225	39,747	34,573	25,299	9.32	5.7	7.22	−0.61	−0.81	−0.70
ERF-VI	6	355	216	271	39,748	24,196	30,327	9.19	4.77	5.72	−0.62	−0.73	−0.68
ERF-VI-L	2	385	265	338	41,535	32,026	36,780	6.68	5.15	6.005	−0.66	−0.79	−0.72
ERF-VII	4	371	287	329	42,402	29,227	36,742	5.82	4.75	5.09	−0.47	−0.96	−0.73
ERF-VIII	13	352	144	248	38,846	16,465	27,309	10.19	4.88	7.84	−0.21	−1.07	−0.68
ERF-IX	17	417	141	242	46,532	14,834	27,132	9.33	4.73	7.05	−0.41	−0.87	−0.66
ERF-X	5	417	171	304	45,288	19,032	33,463	8.69	6.08	6.93	−0.71	−1.08	−0.81
All	100	506	141	276	56,148	14,834	30,683	10.19	4.62	6.49	−0.21	−1.08	−0.69

Note: *p*I, isoelectric point, GRAVY, Grand average of hydropathicity.

**Table 2:** *Ka/ks* analysis in the *MaERF* genes of *M. albus*.

Sequance1	Sequance2	Ka	Ks	Ka_Ks	EffectiveLen	AverageS-Sites	AverageN-Sites
MaERF10	MaERF89	0.642115	0.966072	0.664665	852	200.8333333	651.1666667
MaERF10	MaERF100	0.360433	0.676364	0.532898	1017	250.5833333	766.4166667
MaERF13	MaERF19	0.20672	1.432935	0.144263	519	120.5	398.5
MaERF14	MaERF23	0.233732	0.863167	0.270785	465	98.16666667	366.8333333
MaERF15	MaERF18	0.378074	1.842193	0.20523	720	154.8333333	565.1666667
MaERF20	MaERF64	0.338863	1.741791	0.194549	606	133.1666667	472.8333333
MaERF21	MaERF29	0.344693	1.521477	0.226552	561	125.25	435.75
MaERF26	MaERF88	0.422522	1.191733	0.354545	873	193.5	679.5
MaERF26	MaERF97	0.280705	0.812429	0.345514	990	225.9166667	764.0833333
MaERF27	MaERF99	0.235774	0.406662	0.579779	642	144.4166667	497.5833333
MaERF34	MaERF79	0.204008	0.839805	0.242923	780	167.4166667	612.5833333
MaERF42	MaERF80	0.246629	0.871625	0.282953	579	135.3333333	443.6666667
MaERF43	MaERF48	0.424768	2.126746	0.199727	558	136.3333333	421.6666667
MaERF48	MaERF83	0.372831	1.583792	0.235404	594	138.1666667	455.8333333
MaERF50	MaERF65	0.278726	0.672596	0.414404	846	184.8333333	661.1666667
MaERF56	MaERF66	0.504723	1.158554	0.435649	873	194.5	678.5
MaERF57	MaERF84	0.570869	NaN	NaN	567	130.5833333	436.4166667
MaERF61	MaERF74	0.313703	1.264034	0.248176	576	130.6666667	445.3333333
MaERF90	MaERF19	0.351229	2.145154	0.163731	537	125.1666667	411.8333333

## Supplementary Materials

The following supporting information can be downloaded at: https://www.mdpi.com/article/10.3390/ijms231912023/s1.
