# Supplementary material for: Genome-Wide Identification of ERF Transcription Factor Family and Functional Analysis of the Drought Stress-Responsive Genes in Melilotus albus
Source: Int J Mol Sci. 2022 Oct 10;23(19):12023. doi: 10.3390/ijms231912023 (PMC9570465; doi:10.3390/ijms231912023)

β1 β2 β3 α

```
MaERF001 QSSAKYRGVRMRKW. GRWAAEIRDPFR.......NVRIWLGTYNTAEDAAYAYESKRLHFELRH..QAMDARTCIKKRS 178
MaERF002 GGHVKYRGVRCRPW. GKFAAEIRDSNRQ.......GQRVWLGTFNTAEEAARAYDRAAFNMRGSF..AILNFPN..EYT. 80
MaERF003 KEEVRFRGVRRRPW. GKYAAEIRDPSKQ.......GTRMWLGTFDTAEEAARAYDRAAFNLRDHL..AILNFPS..EYYS 83
MaERF004 RKEKSYRGVRRRPW. GKFAAEIRDSTRN.......GMRVWLGTFDSAEAAAMAYDQAAFSMRGSS..AILNFPV..EVVR 135
MaERF005 QRKNLYRGIRQRPW. GKWAAEIRDPRK.......GVRVWLGTFNTAEEAARAYDKEARKIRGKK..AKVNFPN..EDDA 138
MaERF006 ARGKLFKGVRQRHW. GKWVAEIRLP.......RNRTRVWLGTFETAEDAAIAYDTAAYILRGEC..AQLNFPNLKHVIQ 307
MaERF007 HGTRHYRGVRRRPW. GKYAAEIRDPTRK.......GSRVWLGTFDSEIDAAKAYDCAAFRMRGQK..AILNFPL..EAG. 163
MaERF008 TRHPVYRGVRKRDS. GKWVCEVREP.......NKKTRIWLGTFPTPEMAARAHDVAAIALRGRS..ACLNFADSS.... 103
MaERF009 TTTGRYRGTRCRS.. GKWVSEIREP.......RKTKRIWLGTYPTAEMAAAAYDVAALALKGPD..TTLNFPNSI.... 105
MaERF010 EKRRRYRGVRQRPW. GKWAAEIRDPHK.......AARVWLGTFETAEAAARAYDEAALRFRGNR..AKLNFPE..NVTA 208
MaERF011 ...KHYRGVRRRPW. GKFAAEIRDPNRK.......GSRVWLGTFDTAIEAAKAYDKAAFQMRGSK..AILNFPL..DVAT 144
MaERF012 KNNKSYRGVRRRPW. GKFAAEIRDSTRH.......GIRVWLGTFDSAEAAALAYDQAAFSMRGSA..ATLNFSV..EKVK 143
MaERF013 NKHPVYQGVRMRNW. GKWVSEIREP.......RKKSRIWLGTFPTAEMAARAHDAAALCVKGKS..AILNFPHLA.... 110
MaERF014 KAMKPYRGIRMRKW. GKWVAEIREPNKR.......SRIWLGSYITPIAAARAYDTAVFYLRGPS..AHLNFPELLFKDQ 104
MaERF015 NASCTYKGVRQRTW. GKWVAEIREPNRG.......ARLWLGTFETSHEAALAYDAAARKLYGSD..AKLNLPELS.... 99
MaERF016 VQSKKFRGVRQRHW. GSWVSEIRHP.......LLKRRVWLGTFETAEEAAKAYDEAAILMSGRN..AKTNFPINVEN.. 68
MaERF017 KRKNQYRGIRQRPW. GKWAAEIRDPSK.......GVRVWLGTFNTAEEAARAYDAEARRIRGKK..AKVNFPE..EA.. 178
MaERF018 NPSCPYKGVRQRTW. GKWVAEIREPNRG.......ARLWLGTFETSYEAALAYDAAASKLYESR..AKLNLPEMF.... 94
MaERF019 NKHPVYRGVRMRNW GKWVSEIREP.......KKKSRIWLGTFPTPEMAARAHDVAALSIKGSG..AILNFPELV.... 96
MaERF020 QQTKKFRGVRQRQW. GSWVSEIRHP.......LLKRRVWLGTFETAEAAARAYDQAAILMNGQS..AKTNFPVSKNQ.G 70
MaERF021 RPQQRYRGVRQRHW. GSWVSEIRHP.......ILKTRIWLGTFETAEDAARAYDEAARLMCGTR..ARTNFPYNPNV.. 69
MaERF022 KKKKMYKGVRLRKW. GKWVSEIRLP.......NSRERIWLGSYDSAEKAARAFDAALYCLRGRH..ATFNFPDTPFHLD 93
MaERF023 QEEKQFRGIRKRKW. GKWVAEIREPNKR.......SRIWLGSYITPVAAARAYDTAVFCLRGPT..ARLNFPELLFQDD 94
MaERF024 VRRRHYRGVRQRPW. GKWAAEIRDPKK.......AARVWLGTFETAEAAAVAYDEAALRFKGSK..AKLNFPE..RVQS 137
MaERF025 K...KFRGVRQRPW. GKWAAEIRDPAR.......KVRLWLGTFETAEEAAMVYDNAAINLRGPD..ALTNFLT..PPQK 171
MaERF026 KPTKLYRGVRQRHW. GKWVAEIRLP.......KNRTRLWLGTFDTAEEAALAYDKAAYKLRGDF..ARLNFPNLKHQGS 235
MaERF027 SSKKKYKGVRMRSW. GSWVSEIRAP.......NQKTRIWLGSYSTPEAAARAYDAALLCLKGSSSASNLNFPLTTSSSH 90
MaERF028 RPQQRYRGVRQRHW. GSWVSEIRHP.......LLKTRIWLGTFETAEDAARAYDEAARLMCGPK..ARTNFPYNPNG.. 69
MaERF029 PSNKNYRGVRKRPW. GRYSAEIRDRIG.......RCRHWLGTFDTAEEAARAYDAAARRLRGSK..ARTNFKI..PLVL 134
MaERF030 KNKPHYRGVRQRPW. GKWAAEIRDPKK.......AARVWLGTFDTAEDAALAYDKAALKFKGTK..AKLNFPE..RVVQ 111
MaERF031 TKHPTYRGVRMRAW. GKFVSEIREP.......RKKSRIWLGTYPTAEMAARAHDVAALAIKGHS..AYLNFPNLA.... 120
MaERF032 NSDCNFRGVRQRIW. GKWVAEIREPINGKHV.GEKANRLWLGTFTTAHDAALAYDKAAKAMYGPS..ARLNFPDGSPSS. 147
MaERF033 AWGGRYLGVRRRPW. GRYAAEIRDPST.......KERHWLGTFDTAEEAALAYDRAARGMRGSR..ARTNFVY..HDT. 90
MaERF034 NNTNKFVGVRQRPS. GRWVAEIKDTTQ.......KIRMWLGTFETAEEAARAYDEAACLLRGSN..TRTNFIT..HVSL 100
MaERF035 KRSSIYRGVTRHRWTGRYEAHLWDKSTWNQNQNKKGKQVYLGAYDDEEAAARAYDLAALKYWGPG..TLINFPVTDYTRD 135
MaERF036 NASCEYRGVRQRTW. GKWVAEIREPKKR.......TRLWLGSFATAEEAAMAYDEAARRLYGPD..AYLNLPHLQTHSN 94
MaERF037 PKQIKYRGVRIRPW. GKFAAEIRDSARH.......GARVWLGTFNTAEEAARAYDRAAFAMRGSS..AILNFPH..EHFP 82
MaERF038 TRHPVYRGVRKRRW. GKWVSEIREP.......KKKSRIWLGSFPVPEMAAKAYDVAVYCLKGQN..AQLNFPDEV.... 87
MaERF039 GRHPVYRGVRRRNN. GKWVSEIREP.......KKPNRIWLGTFPTPEMAAIAYDVAALALKGKN..AELNFPNSS.... 86
MaERF040 NNHPVYHGVRMRSW. GKWVSEIREP.......RKKSRIWLGTYASPEMAARAHDVAALSIKGHS..PILNFPEIA.... 100
MaERF041 STTKLYRGVRQRHW. GKWVAEIRLP.......RNRTRLWLGTFDTAEDAALAYDREAFKLRGEN..ARLNFPDLFLNKE 282
MaERF042 NSRCNYRGVRQRTW. GKWVAEIREPNRG.......SRLWLGTFATAIGAALAYDEAARAMYGSR..ARLNFPNVS.... 124
MaERF043 QREIRYRGVRKRPW. GRYAAEIRDPGK.......KTRVWLGTFDTAEEAARAYDTAAREFRGTK..AKTNFPT..PLEI 92
MaERF044 CSS.LFRGVRKRKW. GKYVSEIRLP.......NSRQRIWLGSYDSAEKAARAFDAAMFCLRGSG..AKFNFPNNPPDIA 83
MaERF045 CSSSLYRGVRKRKW. GKYVSEIRLP.......NSRQRIWLGSYDSAVKAARAFDAAMFCLRGSG..AKFNFPNDPPDIA 81
MaERF046 SSL..YRGVRKRKW. GKYVSEIRLP.......NSRQRIWLGSYDSAEKAARAFDAAMFCLRGGG..ANFNFPNDPSNIV 82
MaERF047 ARKNVYRGIRQRPW. GKWAAEIRDPQQ.......GVRVWLGTFSTAEEAARAYDAAAKRIRGDK..AKLNFPD..TP.. 153
MaERF048 VKEAHFRGVRKRPW. GRYAAEIRDPGK.......KSRVWLGTFDTAEEAARAYDAAAREFRGPK..AKTNFPF..PIQS 89
MaERF049 NREGHYRGVRKRPW. GRYAAEIRDPWK.......KTRVWLGTFDTPEEAALAYDGAARSLRGAK..AKTNFP...PAPV 72
MaERF050 TEPGKFLGVRRRPW. GRYAAEIRDPAT.......KERHWLGTFDTAQEAAFAYDRAALSIKGSN..ARTNFIY..PSSS 111
MaERF051 RVEKHYRGVRRRPW. GKYAAEIRDSSKK.......GARVWLGTFNTAEEAALAYDKAALRIRGSK..ACLNFPI..QTVT 211
MaERF052 STHPTYRGVRMRNW. GKFVCEIREP.......KKKSRIWLGTYPTAEMAARAHDVAALAIKGHS..AYLNFPELA.... 125
MaERF053 PCWQRYKGVRRRPW. GKFAAEIRDPNKN.......SARVWLGTYESAEDAALAYDQAAFKMRGSK..ANLNFPH..LIGS 145
MaERF054 SKRLGYRGVRRRPW. GKYAAEIRDPKRN.......GARVWLGTYETAENAALAYDRAAFKIRGSK..AKLNFPH..LIDS 185
MaERF055 KRKNQYRGIRQRPW. GKWAAEIRDPRK.......GVRVWLGTFNTAEEAARAYDDAARRIRGKK..AKVNFPE..EA.. 184
MaERF056 GATVRYRGVRRRPW. GRYAAEIRDPHS.......KERRWLGTFDTAEQAACAYDCAARALRGSK..ARTNFFY..PETN 107
MaERF057 ...KKYRGVRQRAW. GKWVAEIRDPKR.......ATRVWLGTFQTAENAARAYDQDAIKFHGAR..AKINFDF..SDYD 190
MaERF058 VKGKHYRGVRQRPW. GKFAAEIRDPAKN.......GARVWLGTFETAEDAALAYDRAAYRMRGSR..ALLNFPL..RVNS 207
MaERF059 E.KQHYRGVRQRPW. GKFAAEIRDPNKR.......GSRVWLGTFETAIEAAKAYDSAAFRLRGSK..AILNFPL..EVN. 228
MaERF060 ..KQHYRGVRQRPW. GKFAAEIRDPNKR.......GSRVWLGTFETAIEAAKAYDSAAFRMRGSK..AILNFPL..EVNA 226
MaERF061 KHNPTYRGVRMRQW. GKWVSEIREP.......RKKSRIWLGTFPTPDMAARAHDVAALTIKGSS..AYLNFPELA.... 134
MaERF062 TRHPVYRGVRRRNN. NKWVCEVRVP.......NDKSTRIWLGTYPTPEMAAHAHDVAALALRGKS..ACLNFADSA.... 116
MaERF063 KRHPTYHGIRSRG.. GKWVTEIREP.......RKTNRIWLGTFPTPEMAAAAYDVAALALKGGD..AVLNFPNSA.... 76
MaERF064 VQSKKFRGVRQRHW. GSWVSEIRHP.......LLKRRVWLGTFETAEEAARAYDQAAILMSGRN..AKTNFPITQTPEG 70
MaERF065 TEPGRFLGVRRRPW. GRYAAEIRDPTT.......KERHWLGTFDTAQEAALAYDRAAISMKGNQ..ARTNFIY..S... 111
MaERF066 GGTNRYRGVRRRPW. GRYAAEIRDPQS.......KERRWLGTFDTAEEAACAYDCAARAMRGLK..ARTNFVY..PTSP 107
MaERF067 TRHPVYRGVRRRNN. NKWVCEMRVPNSYNKN.NNIKSRIWLGTYPTPEMAARAHDVAALALKGKS..ACLNFADSA.... 121
MaERF068 PA.KKYRGVRQRPW. GKWAAEIRDPAR.......GVRVWLGTFQTAEEAAIVYDNAAIKLRGPD..ALTNFIT..PPSS 162
MaERF069 CKKKLYRGVRQRHW. GKWVAEIRLP.......QNRMRVWLGTYETAEAAAYAYDRAAYKLRGEY..ARLNFPNLKDPTK 182
MaERF070 ENNKKFKGIRRRKW. GKWVSEIRVP.......GTQERLWLGTYATPEAAAVAHDIAVYCLKRPSTLDKLNFPEILSSSYG 83
MaERF071 AASSAYRGVRKRKW. GKWVSEIREP.......GTKTRIWLGSFETPEMAAAAYDVAALHFRGRE..ARLNFPELA.... 70
MaERF072 EGERRYKGIRMRKW. GKWVAEIREPNKR.......SRIWLGSYSTPIAAARAYDTAVFYLRGPS..ARLNFPELLAGE. 87
MaERF073 ATGKHYRGVRRRPW. GKYAAEIRDPAKN.......GARVWLGTFETDEDAALAYDRAAYRMRGSR..AMLNFPL..RVNS 198
MaERF074 KH.PLYRGVRMRSW. GKWVSEIREP.......KKKSRIWLGTFPTPEMAARAHDVAAQAIKGSS..AYLNFPELA.... 96
MaERF075 TRHPVYRGVRKRNL. DKWVCEMREP.......NKKTKIWLGTFPTAEMAARAHDVAAMALRGRY..ACLNFADSA.... 117
MaERF076 TRHPVYRGVRKRNL. DKWVCEMREP.......NKKTKIWLGTFPTAEMAARAHDVAAMALRGRY..ACLNFADSA.... 121
MaERF077 TRHPVYRGVRKRNL. DKWVCEMREP.......NKKTKIWLGTFPTAEMAARAHDVAAMALRGRY..ACLNFADSA.... 121
MaERF078 TRHPVYRGVRKRNL. DKWVCEMREP.......NKKTRIWLGTYPTAEMAARAHDVAAMALRGRY..ACLNFADSV.... 113
MaERF079 EKRDKFVGVRQRPS. GRYVAEIKDTTQ.......NIRMWLGTYETAEEAARAYDEAATLLRGSN..TRTNFVT..HVSY 93
MaERF080 NSRCNYRGVRQRTW. GKWVAEIREPNRG.......NRLWLGTFSNAVGAALAYDEAARAMYGSC..ARLNFPNVE.... 124
MaERF081 NNQSVFRGVRRRPW. GKFAAEIRDSTRK.......GARVWLGTFNTAEEAALAYDQAAFSTRGSS..AVLNFPE..EVVK 169
MaERF082 SNKRVFRGVRRRPW. GTFAAEIRDSTRK.......GARVWLGTFNTAEEAALAYDQAAFSTRGSS..AVLNFPE..EVVK 180
MaERF083 ATEIHFRGVRKRPW. GRYAAEIRDPGK.......KSRVWLGTFDTAEEAARAYDNAARQFRGPK..AKTNFP...PPPP 89
MaERF084 AK.KKYRGVRQRPW. GKWAAEIRDPRR.......AVRVWLGTFTTAEEAARAYDNAAIEFRGPR..AKLNFPL..VDES 153
MaERF085 AK.KKYRGVRQRPW. GKWAAEIRDPRR.......AVRVWLGTFTTAEEAARAYDNAAIEFRGPR..AKLNFPF..VDES 148
MaERF086 AK.KKYRGVRQRPW. GKWAAEIRDPRR.......AARVWLGTFNTAEEAARAYDNAAIEFRGPR..AKLNFPM..VDDS 156
MaERF087 KSRSKFVGVRQRAS. GKWAAEIKDTSK.......NIRMWLGTYKTAEEAARAYDEAACLLRGSN..TRTNFSTTHSIPT 88
MaERF088 KSTKLYRGVRQRHW. GKWVAEIRLP.......KNRTRLWLGTFDTAEEAAFAYDKAAYKLRGEF..ARLNFPHMRFH.. 177
MaERF089 QPKRKYRGVRQRPW. GKWAAEIRDPFK.......ATRVWLGTFENAEDAAKAYDQASLRFRGNK..AKLNFPE..NVRL 195
MaERF090 SNHPVYRGVRMRAW. GKWVSEIREP.......RKKNRIWLGTFATPEMAARAHDVAALAIKGNS..AILNFPELA.... 102
MaERF091 QETCLMRGVYFKN.. MKWQAAIKVD.......KKQIHLGTVASQEEAARLYDRAAFMCGREP...NFELSE..... 169
MaERF092 VKEIRYRGVRKRPW. GRFAAEIRDPWK.......KTRVWLGTYDTAEQAAQAYDTAAIKFRGSK..AKTNFAI..PEHI 114
MaERF093 NSENKYRGVRQRTW. GKWVAEIREPNRG.......SRLWLGTFPTAQEAALAYDYAARAMYGPS..ARLNFPDIADYS. 145
MaERF094 NNKFRYRGVRQRSW. GKWVAEIREPRKR.......TRKWLGTFSTAEDAAKAYDRAAIILYGSR..AQLNLQPSASSS. 122
MaERF095 RRHIKFRGVRQRPW. GKWAAEIRDPTR.......RKRLWLGTFSTAEEAAVEYDRVAVMIHGPN..AVTNFPI..TPVK 188
MaERF096 FKEPRYRGVRKRPW. GRFAAEIRDPLK.......KARVWLGTFDTAEQAARAYDTAARNLRGPK..AKTNFPL..QQPF 88
MaERF097 KPTKLYRGVRQRHW. GKWVAEIRLP.......KNRTRLWLGTFDTAEEAALAYDRAAYRLRGDF..ARLNFPNMKDQ.. 234
MaERF098 QQSCKYRGVRRRKW. GKFAAEIRNPFT.......GKRKWLGTFDTAEDASKAYQDQRLEFTARA..TRLSNENRNKNFK 148
MaERF099 EKKKKYKGVRMRSW. GSWVSEIRAP.......NQKTRIWLGSYSTAEAAARAYDAALLCLKGSS.ATNLNFPLECSSHF 100
MaERF100 EQRRRYRGVRQRPW. GKWAAEIRDPHK.......AQRVWLGTFDTAESAARAYDEAALRFRGSR..AKLNFPE..NVRS 248
```

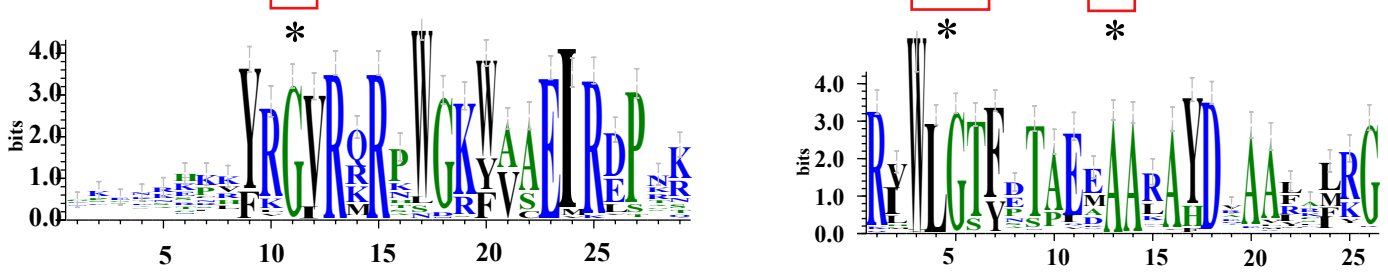

Supplement: Supplementary file 1 [file ijms-23-12023-s001.zip › Figure S2.pdf]
