# Supplementary material for: Genome-Wide Identification of ERF Transcription Factor Family and Functional Analysis of the Drought Stress-Responsive Genes in Melilotus albus
Source: Int J Mol Sci. 2022 Oct 10;23(19):12023. doi: 10.3390/ijms231912023 (PMC9570465; doi:10.3390/ijms231912023)
Supplement: Supplementary file 1 [file ijms-23-12023-s001.zip › Figure S3.pdf]

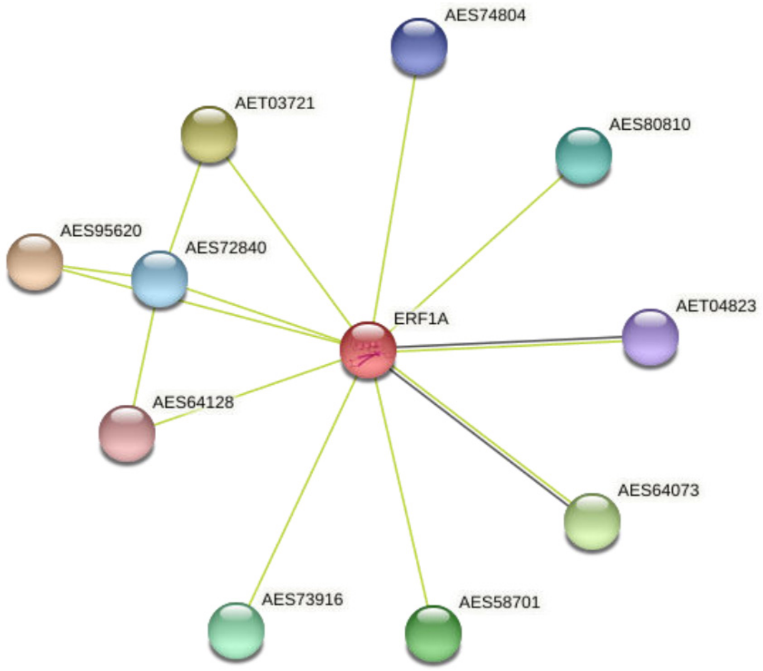

Figure S3: Protein interaction network for 11 stress-related MaERF proteins based on these orthologs in *M.albus*. Black lines indicate proteins that are predicted to interact with ERF proteins.
