# Supplementary material for: Genome-Wide Identification of ERF Transcription Factor Family and Functional Analysis of the Drought Stress-Responsive Genes in Melilotus albus
Source: Int J Mol Sci. 2022 Oct 10;23(19):12023. doi: 10.3390/ijms231912023 (PMC9570465; doi:10.3390/ijms231912023)
Supplement: Supplementary file 1 [file ijms-23-12023-s001.zip › Table S1 .pdf]

>MaERF001
ATGTCGAGTTCTAGAAAATCTAGAGCTAGACAAAGGCCTAACCCTTTGAA
AGAATCCAAATTGATGACAAGGAAATTGCGTGTTGTTTATGATGATCCTG
ATGCAACTGATTCATCTGAAGATGAGTCACAACAACCAAGAACTACAACA
AAAAGAAGCTTCGTTGAGGTTACTCTCCCTCCTTTTGTATCTGCAGTTTC
CTTGGCTTCTCACAACAGTTTCCCTGTTAAGCAAAAGACATCAACAAGGA
GAAAGACACCATGTCTTTCAGCAAAACCTCGAACCAATAAGAAGGTTGTT
CCACCAACAGCAACAACAGGAGGGAGACAATCTTCTGCCAAATACCGAGG
CGTTCGTATGAGGAAATGGGGAAGATGGGCTGCTGAGATTCGCGATCCTT
TCCGAAATGTTAGAATTTGGTTGGGAACTTACAACACTGCTGAAGATGCT
GCTTATGCTTATGAATCAAAAAGGCTTCATTTTGAACTTAGACATCAGGC
AATGGACGCTAGAACATGCATCAAGAAAAGGTCTTCTTCTGTAGCTTGCA
TCAAGAAAAGGTCCCCTCGTTCAGCTGCTGCATCAAACAACTCTTCTGCA
AATGTTGATGCTGTTGTGTCAGAGAAATTTTCAACTACTGAAGACTCTGA
GAGTTTGTTCTCGCACACATCGCCATCTTCCGTGCTTGAGTTGGATACCT
TAGCATCCAATTTGACTGAGAAGTTTGATGATGTTCCATCAAACAATGAA
ATTGGGGCTGTTGATGAAGCTAAAGAAATGGTGGTTTGTCAACTTGAAGA
GCTAGAAATTCCAGATCTGACTGTGTTGAACTTGGCTGAGCCAATTGCTA
CAGAGAATCCAATTGGGACTGATCCCAATCTCGGTTTTGGATTTGACTTT
GATCGGTTTAACATTGATGATTTTGGACCGAATTTTGATGAATTTGGTGA
CTTTGATGTCGGTGACTTCTGTGATTTCAGCGATATTCAAGTTCATGGGT
TTGATGACAATGAGCATAGTGAGCTTCCTGATTTCGATTTTGGTGTTATT
GGTGATGATGATGAATTTGCTGGTTGGATTGAGGAACCCCTCCAACACAA
CATACCTTGTGTCTAA
>MaERF002
ATGAGTGAAGATGGATCAAGGGGAAGGGTGAAGGACGACGGCGGACATGT
CAAGTACCGCGGTGTTCGCTGCCGCCCATGGGGGAAGTTTGCGGCCGAGA
TTCGCGACTCCAATAGGCAGGGTCAGAGGGTGTGGCTTGGAACTTTCAAC
ACTGCTGAGGAAGCTGCTAGAGCTTATGATAGAGCTGCTTTTAATATGAG
AGGTTCATTCGCAATCCTTAATTTTCCAAATGAGTACACTATGGTTGCTG
GTGCCGGTGCCGGTTCCGGTTCCGGTGCCGGTACTGGTTCATCTTCAAGG
AATGCAAATGCTGAAGGCAGAGGAAACCAAGTGTTTGAGTTTGAGTACTT
GGATGATAACTTGTTGGAGGAGCTTCTTGATGTTGAGGAGAAAAAGAATA
AAGGATCCTGA
>MaERF003
ATGGAGGATCACCACAAGGGAAAGCATGGCAAGGAGGAGAAGGGAAAGGA
AGAGGTTCGATTCCGAGGCGTGAGGAGGAGGCCGTGGGGGAAGTATGCGG
CGGAGATAAGGGATCCATCAAAGCAAGGGACAAGGATGTGGTTAGGGACA
TTTGATACGGCTGAAGAAGCAGCAAGAGCTTATGATCGAGCAGCTTTTAA
CTTGAGGGATCATCTTGCAATTTTGAATTTTCCTAGTGAGTATTATTCTA
AAATAAGAGGCTCACCACCATATCCACCTCATTTAGCACCACCATCTTAC
ACCTCTTCATCATCTCATCATGCTAGTGGTAGTTCTTCTGGGCCACAACA
TAGGCCCATTTTTGAGTTTGAGTGTTTGGATGATAAGATATTGGAGGAAC
TTCTTGGGTCAGAAGAGGTGAAGAAGAAAAAGTAG

>MaERF004
ATGGATTCAAGTTCAACCTCAATTCCAAACTCTTCCACTTCAGATTTCTC
ATCAGAATCCTCCTCCTATGAATTAAATTACCTCCCCTTCAACGAGAACG
ATCCCGAAGAGATGCTTCTCTACGGTATGATCAACGACGCAAACAACCTC
AACAACACAACAATGAAGGAAGAAGAGGTAAGTTCCGAAGAGAATGAACC
AAGGAAGGAGAAGTCGTACCGAGGTGTAAGAAGAAGACCATGGGGGAAAT
TTGCAGCAGAGATAAGGGATTCAACGAGGAATGGAATGAGGGTTTGGTTA
GGAACTTTTGATAGTGCTGAAGCAGCTGCTATGGCTTATGATCAAGCTGC
TTTTTCCATGAGAGGTTCTTCTGCAATACTCAATTTTCCTGTTGAGGTTG
TGAGAGAATCGCTTAGTGATATGAATTGTGATGATGGTTCTGAAGATGGG
TGTTCCCCTGTTGTTGCTCTCAAGAGAAAACATTCCTTGAGAAGGAAAAT
TGGTTTCAAGAGAAACAAAGAAAGAGATTTTAGGAGCAATAATAGTAGTG
TTGATAATGCTGTTGTGTTTGAAGATCTTGGTGCTGATTATTTGGAACAA
TTGTTGATTTCATCTGATGAACTTCCCTGTCCTAAGTGA
>MaERF005
ATGTGTGGTGGTGCTATCATTGCTGACTTCATTCCTCGCCGTGACGGCCG
CCGTCTCACTGCCTCAGAGCTCTGGCCTAACTCATTTGGCCAACAAATTG
GCTCCTCAGACTTTGATTTCTCTCATTCTGGTGGTGATCAACAACCACCC
AGTGCTCTCAAAAGATCACAACCTCCCAAAGTTAGTGAACGAGTTGAAAA
GCCGGTGAAAAAACAGAGGAAGAATCTCTACAGAGGGATTCGACAGCGTC
CATGGGGAAAATGGGCTGCAGAGATTCGTGATCCAAGAAAAGGGGTTCGT
GTTTGGCTTGGTACATTCAACACTGCTGAAGAAGCTGCCAGAGCATATGA
CAAAGAAGCTCGAAAAATCCGTGGCAAAAAAGCTAAGGTTAATTTCCCTA
ATGAAGATGATGCATATACCATTCAAGCTTCTCGTCGTTACAATAATAAT
CCACCACCAATTCGACCACAGAAGCTTCCTCTCTATCATCAACAACAACA
ACAACAGTATCCAAAGAATCTGAACTTGGAGTTTGGTTATGATCTGAACC
AAACTGATACAGTTCAAAATTCTCATGTGGGTGCTATCAATACTGAACCT
TTTAACATATCTGGTTCTGGTGGTGATGAGAATTCTTTATTTGGGTCTGG
TTCAGGTTCTGGTTCAGAAGTTGGTTTTTCTTTGATGGAGTTCAATGGTG
CTGCTAATCAGAATGAAACTGGTTATTTTGGTGGTGTTGTGAATGAACAT
GAAAAAGAGAAAGAGAAAGTGGAAGAACAAAAAGAGAAAGAAAAACATGT
TATTGATCAAGCTGAAGCTGAATTGGCAAAGAATGAAGTGCAAGAGTTGT
CTGATGAATTGTTGGCATACGAGGATTATATGAAGTTTTATCAGATTTAC
TATGATGGACAATCTGTGATGCCACCAAACATTGTTCAGGAACATGTGGT
TGGAGATTTATGGAGCTTTGATTGA
>MaERF006
ATGGAAGATACATTTCCCAAGATGGAAATCTTTACACAAAAGGAATTACC
AACTTGTTTGCAAGAAATAGGAACAACAGAATCCAAGTATTTGGAGGATA
CAATTATAAGAAGAGGCTCTTACTCTCTCACTTCATCAACAGAATCACCA
CAGAAACTTTTCTGGTGTACTTCAAATTCTACTTCAATGAAACAAGAGGT
ACCTTTAACCACTTTAGTTAATCAAATGCCTGCAAAGTTCAATGATCATC
ATGATGACATGAAGAATATGAAAACTATAAACCAACCCATTTCTGAATTC
AATTCTATCACCAATCCTAATGTCTTAGCCTCAGATATAAAACATAGTTT
TGTTCCTCTAAATCTTTTGGAAACTCTCCCGGCTTTAACCGTAACTCCGG

TGTCTGATGAGCCTACAGTTTCTTTAGCTTCTTCTTCTACTATACCGAAA
TATCCAAATTTGACTTTGTTTTTGCAGGAACCATCCATGCTATACTCATC
ATCCTCACTTATCCAAAGTAGTGAAATCATGTCAACAACTTCAAATCCCA
CATATCCTATGTCTCAATTTGGCCTAACTCAGCATCATCAACAAAGTGGT
TATGAATTTTATGATTCTAACAAAATGAATAAGAATATGATGACAAATAT
GCAATCCAAGTCTTTCAATGAGAATTGGTTGAGTACCACAAAGACTCAAC
CTTTGAAGTATGGTGCAAGAGGGAAGCTTTTCAAAGGGGTAAGGCAGAGA
CATTGGGGAAAATGGGTAGCTGAAATTAGGCTACCAAGAAACAGGACTAG
GGTCTGGTTAGGGACATTTGAAACAGCTGAAGATGCTGCAATTGCATATG
ATACAGCAGCATATATACTAAGAGGAGAATGTGCTCAGTTAAATTTTCCA
AATTTAAAGCATGTGATTCAAGCCAATTCATTGAATGGAACAACAGCTTC
ACTTGTTGAGGCAAAACTGCAAGCAATATCACAAGGTGTTTCTTCTTCTC
ATAGAAAGCAAGGTGATTCATCCGCAAGGTTAAACAATAAGCACATTGAT
GAGATTAAAAATGGAAAGGATGAAAGTATAAATGAGTTGAAGGTTGGAGG
TGACATAACTGAGAGAAGCAAAAGTACACAGAATGAGATATGTGATGTGG
AAACTGTTCAGTTAAGCAGAATGCCATCTTTAGATATGGATATCATTTGG
AATGAACTTCTAGTTTCTGATTCATGA
>MaERF007
ATGACAACATCAGAAGAAACATCAAACTTGAAACTATTGAGCCAACATCT
TTTAGAGGATGTATCAGACTCATTTTTCACCAATCTTAGTTCTATAAAAC
TTGATTACACATCATCATCACCAGAAATTGACTTGGATTCATATTTTTCA
GAACCAAACAGATTTCTTGAAAGCCTTGGTTTTGAAGCTGATACAAAAGT
TATTGACTTCACTTCTTCTCATAACAAGGTACGAAACACTAAAGCTGAAA
GTCTCTTTGAGGAAACACAGATGAAGGAACAAAGATGTCATGGAACAAGG
CATTACAGAGGAGTGAGGAGAAGGCCATGGGGAAAATATGCTGCAGAAAT
TCGTGATCCGACAAGGAAAGGAAGCAGGGTTTGGTTAGGAACATTTGACA
GTGAGATTGATGCTGCTAAGGCTTATGATTGTGCAGCTTTCAGAATGAGG
GGTCAGAAAGCTATATTGAATTTTCCTTTGGAGGCTGGTGAAGCTAATCC
AAAGCCTAATAACTGTGGCAGGAAAAGGAGAATACATCAAAGATATGATG
CTACAAGTTCAAGTTAA
>MaERF008
ATGTTTACTATGAATAATATTTCGGAGGCACATGATCCATGTTCATCATC
TTCAGAGAGGTTTCTTGCAGAGACTATGCCGAAGAAGCGTGCAGGGAGGA
AGAAGTTCAGAGAAACGCGCCACCCTGTGTACCGTGGTGTTAGAAAGAGA
GATTCCGGAAAGTGGGTTTGTGAAGTAAGAGAACCTAACAAGAAGACTAG
AATTTGGCTTGGAACATTTCCTACGCCGGAGATGGCGGCGCGGGCGCACG
ATGTGGCGGCGATTGCGCTGAGGGGAAGGTCTGCCTGTCTCAATTTTGCT
GACTCTTCTTGGAGGTTGCCGGTTCCGGCAACAGCGGATGCTAGGGATAT
TCAAAAAGCGGCGGCAGAGGCAGCGGAGGCTTTCAGGCCGGAAGCTGAGT
TTGAGAATAATGAAGGGAGGAAGGATAGTGAGGAGTTGTCAACGGTGGCG
GTGGCGGTGGCGGAGACGGTGATGGAGCAACGAGAGGAGGAAGAAGATAC
GGTGCCGGAGTATTTGAGAAACATGGTACTTATGTCGCCGGCACATTACT
GGGGGAGTGATTGTGGTGTTGCTGACGTGGAATTTGATGAAACTGAAGTT
TCATTGTGGAGTTATTCATTTTAA

>MaERF009
ATGGATGGCACCAACAGCAACACTAATTATAACCCTTCAGCAGTGCCGCC
ACCAATACAACTCCCTGATGTACTAGTAACCTCTTCATCATCACCACCAA
CAACTCCTTCATCTTCTCCGGCAACAACTACCGGACGATACCGTGGAACC
CGTTGTAGGAGTGGGAAATGGGTATCTGAGATACGTGAGCCACGTAAAAC
AAAGCGCATTTGGTTAGGAACATACCCTACAGCTGAAATGGCAGCTGCTG
CTTATGATGTTGCTGCTCTAGCTTTAAAAGGACCTGATACAACTCTTAAC
TTTCCCAACTCCATTCACTCTTACCCTATTCCTGCTTCATTGTCTTCAAC
TGATATTCGTGCTGCTGCTGAAGCTGCTGCACAGGCCAGAATCATGAGAC
CACCATCTCAACAACAACAACAACAACAAAACCCTTCAGCATTTGGTTTT
GATCAGGGAAGTTCTTCAGCTAGTGGAGTACAACAAGGTCATCATCATGA
ATATATTGATGAAGATGAGTTGTTGAACATGCCGAATTTGTTAGATGAAA
TGGCTAGAGGAATGCAGTGA
>MaERF010
ATGTCTTGTCCTATTGTTTCAGCGCCGACTCATGGATTGGGCCAGGCCCA
AAATGAGTGGGTCCAACAAGTCCAACAACAAGGAAGTGGTTATTTTCCTA
TGATGTCAGGTTTTGTTCCTGTCTCTTCTAGTTATAATTCTCCTCGTATG
AGTTCTTCTAGTGGTGCTCTTTTGGGGTCTGGTTCTGGTTCTTGGGTTGG
ACATAAAAGAGGGCGTGAAGATGGTGTTCATAGGAGTGATGATGTTGTTG
GTGGTTCTTCTCAACAAGATATGACTACAATTCATAGAAATATTGCTGAT
TTCAGATTACCTTCATCCCAGGGAAATTCATCATCAGCAACAGAAGCAAC
TGCAACAACTACAAGTACAAGCAACACAACACAATCAAGTGAAGCAGCAG
CAACAAACGAAGAAGAAACCGGCGAGAAAAGAAGAAGATACAGAGGAGTG
AGACAGAGACCATGGGGAAAATGGGCAGCAGAGATTCGAGATCCACACAA
AGCAGCAAGAGTATGGCTTGGAACATTTGAAACTGCAGAAGCTGCAGCAA
GAGCTTACGATGAAGCTGCATTAAGATTCAGAGGAAACAGAGCAAAACTC
AACTTCCCTGAAAATGTTACAGCAACAAGACCAACTTTTCCGACGAATAT
TTCAACGGTATCAGTTTCTCCGGCAACACATTATTCACCTGCACCACCGC
AAATGCAGCCACAACAGCCATTTCTTCAGTTTCAAAGCTCTTCTGATTTG
TTGAGAGACTATTATCAATATTCTCAGATTCTGAAAGGTTCTGGTGATTT
TCAAGGGCTTGAACAATGGTTCTATGAATCTCAAATGGCAGCAATGCATT
CATCTTCTTCGATGTTATCGCCAACGCCGTCACTTTCCTTGTCATCTTCT
TCATCTCCAACTGCATTTTCACCTTCCAGTCAATTATCCTCTGTTTCACT
TCCCCTGTTTCCTGGTCAGCAAATGGAGTTTTTCCGGCCACCGGAAGATC
ACTCTCGCGGCGGAGGATACAGTGGTGGGTCCCATTTTCCACCTTCAACA
TGGTCAGATACTGGTGGTTATCATCCTCCTCCACCACCACCACCTTCTTC
AAGTTGA
>MaERF011
ATGGAACAAACCTTTGAACCCACCTTCACTTTTTGGTCCTCAAACATCCT
AGAAATTCAAAATTCAATTTCTCAAAGCAACAACATGACACAAACCTCAT
TCTCCAAACCTCCAAGATCATCCTCAAACCTAAGCAACCGCAAACCCTCC
TTAAACATCACAATCCCTTCCATAACCAAACCTCTCCCACATCAAAACAT
TGAAAAAGGTGAAAACATCAACACCAAAGAGGATAACAAGCACTATAGGG
GTGTTAGAAGAAGACCATGGGGAAAATTTGCAGCAGAAATTCGTGACCCT

AATAGAAAAGGGTCAAGGGTGTGGCTTGGAACCTTTGACACAGCTATAGA
AGCTGCAAAAGCTTATGATAAAGCTGCTTTCCAGATGAGAGGAAGTAAGG
CCATTTTGAACTTCCCTCTAGATGTTGCTACTGACTCTGTAGAATCATCA
CTTTCTAAATGCATCAAAGTTGGTGAGAAAAGGCAAAGGGAAGAAGAAAA
CTGTGTTGAGATCAATAATAATACTAAGCAAGTTAAGAAAGAAGAAGGGG
TTTCAGCAGCAGTAACAATTTGTCCTTTAACTCCTTCATGTTGGAAAGGC
TTTTGGGATACTGATGTCATGGGAACTATCTTTAGTGTACCTCCTTTGTC
ACCCTTATCTCCACTCATGGGATTTTGA
>MaERF012
ATGGATTGTTCCTTCATTCCTTTCTCATCAAAGTCCAATTTTTTTCCACA
ATCTCCATTTGTGTCACAAGATTCAATCTCTTTGAATCCAAAAAACCAAG
AGTTTCTCCCTTTCAATGAAAATGACCCAGAAGAGATGCTTCTATATGGT
ATGATAACATCATCACCACAACAACAAACTCTAATTTCCAATAGCAAAGA
AACAACAAAAAAGAACAACAAAGAGAAGAACAACAAGTCCTATAGAGGTG
TTCGAAGGCGTCCATGGGGGAAATTCGCGGCCGAGATAAGGGACTCAACA
AGACATGGCATAAGGGTATGGTTAGGAACATTTGATAGTGCTGAAGCAGC
TGCACTTGCTTATGATCAAGCTGCTTTTTCAATGAGAGGTTCAGCTGCAA
CACTTAATTTTTCAGTTGAGAAGGTTAAGGAATCACTTAGGGACATGAAT
TATGTGTTGTCTAATGATGATGAGTGTTCTCCTGTTGTGGCTTTGAAGAG
AAAACACTCCATGAAACGTAAAATGGATGAAAAGAAGAAGAAACATGAGA
CAGATGTTAGGATAGATAATTTGGTTGTGTTTGAAGATCTTGGTGCTGAT
TATTTGGAACAATTGTTGATGTCTTCTGATGACAATCAAAATACTTGGTG
A
>MaERF013
ATGAGAGTTTCACCACAAAATTCAGAAACCGAGAGCACCTCAAATTCATC
ACCACCAACATCACCATCCTTAAACTCAATTCAAGAGAATCATGATCAAG
AATCCAATAAGAAGCTTAAACGCAACCGTGACTCCAACAAACACCCCGTC
TACCAAGGCGTTCGCATGAGAAACTGGGGAAAGTGGGTGTCTGAAATCCG
CGAGCCTCGCAAAAAATCGCGGATTTGGTTAGGCACTTTCCCCACAGCTG
AAATGGCTGCCAGAGCGCACGATGCTGCTGCGCTCTGTGTTAAAGGAAAA
TCCGCCATTCTAAATTTCCCTCACTTAGCCAATTCTCTTCCAAAACCCGC
TTCTTTAGCCCCTCGCGATGTCCAGGCCGCGGCGGCTAAAGCAGCTAAAA
TGGACATTAGTAAATTTGAACTATCCTCATCTTCTTCTTCATTACCATCG
TTGTCGTCTTCCCTGACAACGACGTCAACATCAACAAGTCTGTTAATGAA
GACAACGAAAACACCGTCTTCATCAGCAGACTTGTCTGCTACTTCGGAAG
AACTTAGTGAGATAATTGAGCTACCTACTTTGGAAAACGGTTATGATGAT
GTTGGGAAAGAGGAGTTTGTGTTTGTTGACTCACAATCACAAGACTATGC
TTGGATGATGTATCAGCAACCAATGACATGGCTACAGACAACACAAGAAG
ATGGTTGTTGTGTTGGTGATGATGGATTTGTTAATAATAATGGTGTTGTA
ACTAGTTTTGAGAGTTTTCTTTGGAATTACTAG
>MaERF014
ATGGAAGAAGATAGGGAATATTGTTGTTCTATGAATTCAAGAACCAATAG
CCCTATGGCTATAGATAATAATATCAACAACAATAAGGGTATGAAAAAAG
AGAAAAAAGCTATGAAACCATATAGAGGAATAAGGATGAGAAAGTGGGGT

AAGTGGGTGGCAGAGATTAGAGAACCAAACAAGAGATCAAGAATTTGGTT
AGGTTCTTACATAACACCTATTGCTGCCGCACGTGCCTATGACACAGCTG
TGTTCTATCTTAGAGGTCCTTCAGCACACCTTAATTTTCCTGAATTATTG
TTCAAAGATCAACAAGAAGATGAAGAAAATCTACAACAACATGGGAATAT
GTCGGCTGATTCAATAAGGAAAAAAGCTACACAAGTTGGTGCTAGAGTTG
ATGCTCTTGAAACTAATTCTCTAATTAATCATCATGCTTCTTCCAATAAT
CATCATGATCACTCTAATTCTTCTTTTACTTCTCTCAAACCTGACTTGAA
TGAGTTTCCAAAACCTGAAGATTGTTGA
>MaERF015
ATGATGAAATCAGGGATGGCAATTGAAGAGAGAAAACAGTTGAAAAGGCC
AGCACAAGCAAGTTCAAGAAAAGGGTGTATGAGAGGTAAAGGAGGACCAG
AAAATGCAAGTTGCACATACAAAGGTGTGAGACAGAGAACATGGGGAAAA
TGGGTGGCTGAAATTCGTGAACCTAACCGTGGTGCACGTCTTTGGCTTGG
TACTTTTGAAACTTCTCATGAAGCTGCTTTAGCTTATGATGCTGCAGCTC
GTAAACTTTATGGTTCTGATGCTAAACTTAACCTCCCTGAACTTTCTGCA
CCCCCTCAAAATAATTCTACACCCTCTCCTACACCTCAAATGCAACAACA
ACATCCTCATATTCAAATTCAACCCAACAACAACCACAACAACAATAGTT
TCAATATCTCAAATCTCAATTCTTGCAACAACATCAACATGAACAACAAC
ATCAACAACAATAGTCCTGTTTTTGTGTCTCTGTCTTCTCAACAAGTTGG
TGATATTACACCAATATATAGCAGTGATAATTCTGTTATGTCATTTCCAT
TGGATAGTACTATTACAAATACAATGGATACAAAGGGTATGGAGATTAGT
ACTGATTCATTTTTTGGAACAGTGAATTATGAAACCATGCCAGTGATTGA
TGATGATTCAATTTGGACAGAAGCTGCTATGTCTTTAGATGCTGCAATTT
CAATGGATTTTCCAATGATTGTTGATGATGCTGATGGAATTTATAATTCA
GGTGGAAATTTTGCTGAAGTTGGTGCTTGGGATTCTCTGCAAACTCCATG
GTGCATGTAA
>MaERF016
ATGGTACAATCAAAGAAGTTCAGAGGTGTCAGGCAACGCCACTGGGGCTC
TTGGGTTTCTGAGATTCGTCATCCATTATTGAAAAGGAGGGTGTGGTTAG
GAACATTTGAAACAGCTGAAGAAGCAGCTAAAGCATATGATGAAGCAGCA
ATTTTGATGAGTGGAAGAAATGCAAAAACTAACTTTCCAATTAATGTGGA
AAATCAAACCAGTTCTATTTCATCTTCATCTTCAACTTCTTCCAAAGCAT
TTTCTGCAGTTCTTAGTGCAAAGTTAAGGAAGTGTTGCAAGTTTCCTTCA
CCTTCACTCACTTGCTTGAGGCTTGATGCAGAGAATTCACACATTGGTGT
GTGGCAGAAAGGTGCAGGTCCTCGTTCTGAATCAAATTGGATTATGATGG
TTGAGTTAGAAAGGAAAAAAAGTGATTCTGAGCCAGAGAAGGTAAAGCCA
GAGGAATTAAGTAAAAATGGTTTGGATGATGAACAGAAAATTGCTTTGCA
GATGATAGAAGAACTTCTCAATAGAAACTAA
>MaERF017
ATGTGTGGTGGTGCAATTATCTCCGACTTCATCTCAACTGCGGCGGTGGT
CGGTGGTTCCCGCCGTGTCACCGCCGACATGTTGTGGCCCAATTTCACGA
AACCCACGTCGAGGAAACCATTTTTCCTTGACGATGATTTCGAGGCTGAG
TTCAGAGAGTTTGAGGATGATTCTGATTTCGATGAAGACGATGACGAGGA
TGATAAGGAAAGGTTGTTGGTCGGTGTCAAAGGATTTACCTTTGCTACAA

CTAACAAAAAATCCTCTGTTCGTGGATCAAGTGCTGCAAAGTCTGTTGCA
TCGAAATCAAACGAGCAAGGTGAAAAGGAATTGAAGAGAAAGAGGAAGAA
TCAGTATAGGGGTATCCGCCAACGTCCATGGGGAAAATGGGCCGCTGAGA
TCCGTGACCCAAGTAAAGGAGTTCGTGTTTGGCTCGGAACTTTCAACACT
GCTGAAGAAGCTGCAAGAGCTTACGATGCCGAAGCTAGGAGAATCCGTGG
CAAGAAAGCCAAGGTGAATTTCCCCGAAGAGGCTCCTAATGCTTCTTCAA
AACGTCTGAAGACAAATCCTCCTGGGACACAGCTGTTGAACAAAAATCTG
AACTCTTTCAAGCCGAGCGGAAATTTCAGCGAAAATATGGAGAACTACTA
TTCCCATATGGATCAGGTGGAACAGAAAGCATCGGTTGACAACCAGTATG
CTAACGTGGGAGCTTTTGCTGGAAACGGTGTTCAGTTTGCATCTGCTGAT
GTTAATGCTTATTTCAGTTCAGAGCATTCAAGCAATTCGTTTGATTATCC
TGATCTATGTTGGGGCGAACAAGGCCCAAAAACACCTGAGATTTCGTCTG
TGTTTTCTGCTCCTGCTCCTATTGAAGGCGAATCTCAGAAGAATCTGCTG
TCTGGCAACTCTCAGGATATGCTACCTATGCAAAATGATTCTGAAAAGAC
AAACTCTGAGGAGCTTGCAGATATTGAATCCCTGCTGAAGTTCTTTGAGA
ACTCGTTTGATGATAACTGGAGCGATGCTTCATTGGCAGCTCTGCTTGAT
GGAGATACAACTCAGGATGGTGGAAATACAATGAACCTTTGGAGCTTTGA
TGACCTGCCTTCCATCGCAGGTGGAGTTTTCTGA
>MaERF018
ATGGAAGTTCAGGAGAGAAAAAAATTGAAGAAGCCTCAACATGCCACCTC
CAGAAAAGGTTGTATGAAAGGTAAGGGAGGACCTGAAAATCCGAGTTGCC
CTTACAAGGGTGTGAGGCAGAGAACTTGGGGTAAATGGGTTGCTGAAATC
CGTGAGCCCAACCGTGGTGCTCGCCTTTGGCTTGGGACATTTGAGACATC
CTATGAAGCTGCCTTGGCTTATGATGCCGCCGCCAGTAAGCTTTATGAAT
CAAGGGCCAAGCTCAATCTACCTGAAATGTTTGTGAAATCTCAAGGTAAA
ATATCTTCCTCTGACACTCAAATTATTCGAATGGAAGACTCATATCAGCC
TCCAATTAATCACAACGCAAATATGGACACATCTTATGACGTCAATACCA
ACCCATACCCAACAATGTCAATGGCATCTCAACCATTGGACCGCAACAAC
ACCAACTCGCACCCAACCATGTCAATAGCTTCTCAGCCATTAGACTGCAA
CAACTCCACACCCTCTTTTCCCTTGGATACCAACCCTAACCCAATTGATC
AAACCTACAAGGAAATTTTTCCACCTTTGGAAAGAATGAATGATGGTTTT
GCAAGTTTAGATGATTCCATATGGCCAGAAGATGTCATGTCAATTGATTT
TCCTATTAATTATGCAAATTCTGGAATGATCACAGAGGCAAACTTGACAA
ATGGAAGTGTTTGGGACTCATTGCAGAAGCAATGGTGCATGTAA
>MaERF019
ATGAGTAGTACTACTATTCAACAACCTCATAACTCACAAACTGAGAGTAG
CTCAAATTCACCATCACCACCAATTAAACGCATAAGAGACACAAACAAGC
ATCCAGTATACCGCGGGGTCCGAATGCGAAATTGGGGAAAATGGGTGTCC
GAAATTCGCGAGCCAAAGAAAAAATCTCGAATATGGCTCGGCACATTTCC
CACACCGGAAATGGCAGCTCGAGCACACGACGTAGCTGCTCTTAGCATAA
AAGGAAGCGGCGCCATTCTAAATTTCCCCGAATTGGTTAACTCTCTCCCT
CGGCCCGCTTCACTGGCTCCCCGCGATGTCCAAGCCGCCGCAACTAAAGC
CGCTCATATGGAGTTTCCATCTTCCACAACTTCTTATGAATTGAGTGAGA
TAATTGAGCTTCCTCACTTGGGAAACAGTGGAGATTTTGGAAAAGAGTTT

GTGTTCATGGATTCTATTGATTCTTCATGGATGTATCAGCCTCCTTGCTT
GCACACTATGGAAGATGTGATTTGGAGTGGTATTTATAATAACTAG
>MaERF020
ATGGTACAACAAACAAAGAAGTTCAGAGGAGTCAGGCAGCGCCAGTGGGG
CTCTTGGGTGTCAGAAATTCGCCATCCTTTGTTAAAGAGAAGGGTGTGGT
TAGGGACATTTGAGACAGCAGAGGCAGCAGCAAGGGCATATGATCAAGCT
GCAATTTTGATGAATGGTCAAAGTGCAAAGACTAATTTCCCTGTGTCAAA
GAATCAAGGTGAAGAAGTTGCTATTGACACCCCTTCTGATGACTCTTTCT
TGTCTCCCAAGGCTCTCTCTGAGCTACTCAGCACAAAGCTTAGAAAGTAT
TGCAAAGACCCTTCTCCATCACTCACTTGTTTGAGGCTAGATAATGATAA
TTCTCACATTGGAGTGTGGCAAAAAAGAGCTGGACCACATTCTGATTCTA
ATTGGGTCATGAGGGTTGAACTTGGTGGAAAGAAAAAGACTATAGAATCA
GAGATAGGGTCAACAGAACACATTATTGATGGTGGTAATAATGGTAATGC
TGATAATGAAAATAATAGGGTGGTAGTAGAAGAAGAGGAAAGAGTTGCTT
TGCAGATGATTGAAGAGTTACTTAATTGGAACTATCCATGTGGTTCAACT
TCAAGTAATTAA
>MaERF021
ATGGCTAGACCACAACAGCGTTACCGAGGCGTCCGACAAAGGCATTGGGG
CTCTTGGGTCTCCGAAATTCGCCACCCCATATTGAAAACTAGGATATGGC
TTGGTACTTTTGAAACAGCAGAGGATGCAGCTAGAGCATACGATGAAGCA
GCAAGACTAATGTGTGGTACAAGAGCAAGAACAAATTTCCCCTACAACCC
CAATGTGTCACAATCATCATCTTCTAAGCTTCTCTCAGCAACTTTAACTG
CTAAATTGCATAGATGCTATATGGCTTCATTGCAAATCACTAGGCCAATA
TCTCTGCAACAGCCTCAGAGAGAATCTTCACAAAGTAACATGATCTCCAC
AACTAATGTTCCTATGAAAAACAGCAAAGAAATTGAGACTTCTATGAAAC
ATGAAGAGGAGCAAGAATCAGAGGGAAATTGGGTTTTCAAGAAAGTTAAA
GTGGAAAATTCTCAGCAATTTATTAAGCCTCTTGAAGAAGATCACATTGA
ACAAATGATAGAGGAATTGCTTCATTATGGATCTATTGAGCTTTGCTCAG
TTTTTCCACCACAGACATGA
>MaERF022
ATGGATCAAATTAATATCCCATTGTCATCATCTTCTTGTTCTAGTGGTAA
TGATACTAATGATAATAATAATAAGAAGAAGAAGATGTACAAAGGAGTGA
GATTGAGAAAGTGGGGAAAATGGGTATCTGAAATAAGGCTACCAAATAGC
CGTGAAAGAATATGGTTAGGATCATATGATTCAGCTGAGAAAGCAGCTAG
AGCATTTGATGCTGCACTTTACTGTCTCCGTGGTCGACATGCAACATTCA
ATTTTCCTGATACACCTTTCCATTTGGATATCAATAATGTATCTGCAGCT
TGTAACAATTCTCTTACACATCAACAGATTCGAGAGGTTGCTGCGAATTT
CGCCAAAATTTGTCCACCAATTATTGATAATAATAATAATAATAATAATG
GTAATGCTCTTCAATCCAATATCGTGACGGAAATTAGTGGCTCTTCATCT
TCTACTACTATGTATGATAATGAGAGCACGATTGATTGGACATTTTTGAA
CATGTTGGATGGTTCAAGTACTCATGATGCTAATTTTGTTGGGTCTGAAA
ATATTGGTGGTTTCTATTCTGACTTAGAAAAGATGCATTCGGGTGAGTTA
ATATATTCCATTCCACCACTGATTTTTGAGGATAATCAGAACGAATTAGT
TGAAGGCCATGATGATGTTGATGATGACCCATTTTCTCATCAACCAATCC

TTTGGAACTGGAACTTCTGA
>MaERF023
ATGGAAGAAGAAAGAGATTATTCAACAATAGGAAGCAACAACACTCCCCA
AGAGAAGAAGCAGAAGCACAAACAACAAGAAGAGAAACAATTCAGAGGAA
TAAGGAAGAGAAAGTGGGGTAAGTGGGTAGCAGAAATAAGAGAACCAAAC
AAAAGATCAAGAATCTGGTTAGGTTCTTACATCACTCCGGTAGCCGCCGC
ACGCGCCTACGACACCGCCGTTTTCTGTCTGAGAGGTCCTACTGCTCGTC
TTAATTTTCCCGAATTATTGTTTCAAGACGACGAAGAAAACAACGAAGGT
TCTGTTCAACAAGGTAACATGTCTGCTGACTTAATACGCAAAAAAGCTAC
AAAAGTCGGTGCTAGAGTTGATGCTCTTCAAATTGCTCTTGATCAAGCTT
CATCTCGTACCAATTCGGCTCAATTCAACTCTGATTTGAACGAGTTTCCA
GAACCTGAAGATTATTAA
>MaERF024
ATGGATAGAAGGAGAGAATCTTGGGAAGAAGAGGGAAAAGAAGACGAGAA
TTTGTTTCCGGTGTTTTCTGAAAGGTCGCAGCAAGATATGTCAGCCATAG
TATCTGCTCTTACACAAGTCATGGGCGGCAATAACAATAATGAAATGCAT
GAAGCTTCCTCAATCCATAATATTGAACAATCTCAACCACCACAGCAAGA
TCAAGGCAATGTAAGAAGAAGACACTATAGAGGTGTGAGGCAGAGGCCAT
GGGGAAAGTGGGCTGCAGAGATTCGCGATCCGAAGAAGGCAGCAAGGGTA
TGGCTTGGAACCTTTGAAACTGCTGAGGCTGCAGCTGTTGCCTATGATGA
AGCTGCACTCAGATTCAAAGGAAGCAAAGCTAAGCTCAATTTTCCTGAAA
GGGTTCAAAGTACAGCAGAATTTGGATATAACCAAGAATATCATCACCTA
GTGTCAACCACAAGTAACGACCAACAATCTAATCCAGTTCCTCATCCACA
CTTTTCTCAAGAAACATATCCAAATCCTTATCAATATGCTGCACAAGCAC
ATCTTCAAGCTAGTGGCAGTAGCAACTTCAATCAGGATATGCTTCGTTTC
TATGGGAGAGATATGTTTGTTTCTAATTCTCAGCCCTTATCAACTGCATC
TTCATCTTCATCATCATCATCTTCTGGATTGTCTCAGCAGCAACAAGAGC
TTCTGAGATTGTCGATGCAATATGGAGGTTCTTCTTCTTCCTCTTCTCAG
CCTCCAAGGAATTGGAGGGATGACATGGATGGGAGACAATGA
>MaERF025
ATGGAAAAACAGAGTATCCTATGCAAATACACAGAACATGAAACTGTTAC
AAAAAAACTCATCACAACAAAGAAAAATGGTCACAACAACAACACAACCA
TAAACCCAAGAGTTGTCAGAATATCTTTTACCGACCCAGATGCAACCGAC
TCATCCAGCGATGAAGAAGGCCAAAGACAAACCTTCAAAAACAGAACAAA
ACGCTATGTAAACCGTATTGAAATTGAAACAACCGCAAAAGCGGTTGTTA
ACAGAAAAAGACCCGCCGGAGAAACAACAACCGTTCGACGGCCGGCGAAG
ATTCCGGCAGTCAACAACGGTAAAAAGTTCCGTGGTGTACGGCAGAGACC
ATGGGGAAAATGGGCTGCTGAGATTCGAGATCCTGCTCGGAAGGTGAGGC
TCTGGTTGGGAACATTTGAAACTGCTGAAGAAGCTGCCATGGTTTACGAC
AATGCCGCGATTAATCTTCGTGGCCCAGACGCTTTAACAAACTTCCTCAC
ACCGCCGCAGAAAGAAACTCCGGTAGAACAACCCTCCGCCGTGAAGCCAG
AAATGAAGGTAATTGTTGACGCCGATGTTGAAGCCGAAGCTTCTGGGTTT
GGTAACTCTGGTTATGATTCCGGTGAGGAACGTTGTGTTCCACTTTCTTC
TCCAACTTCGGTATTAAATTTCCGGAGTAACTCCGGCGAATCACAAGAAT

CAGAAGAACGCAGTCAAAGTGAAGCTTGTGGAGTGTTTAGAGAATGTCAA
GGTGAATCAAATCTGTTCGATGAAACTGCGTCGTTTTTTCAACATGATAT
GCCTTCTTGGGATGATGTGTTCAACTTTGAAACTCCAGAGTTTCCTTTGA
TGTTTGAAGAAGAACAGAGTGAAATGTTGTTTGGAGAAAATACAACACCG
TTTTTGTTTGATGAAGATTTAAGTGCCAGCATTGTTCTTGCTGACTCACT
CATAGATTTTGACAAAGCATGTTTTCCTTCACCTTCTCCTCCTTCTTCAT
CGTTATGCCAAGTGGATGATTTCTTTCAAGACATTTTGTTAGGCTCAGAT
CCTCTTGTTGTGCTTTGA
>MaERF026
ATGGCAGCTATGATGGATTATTACAGTAACATGCAACAATTTCACAGCTC
AGATCCATTTAGAGGTGAATTAATGGAAGTTCTTGAACCTTTTATCAAAA
GTCCTTCTTCAACTTCATCTTCAACACCATCACCTTCTTATTCTTCTTCT
TCTTCTTCTTCTTCTTCTTCTTCTTCTTCTTCTTCTTCTTCTTCTTCTTC
TTCTTCTTCTTCTTCTTCTTCTCTCTCTCCACCTTCTTTCTCTTCTCTCC
CTCCTTTCTACACAGAGCAAAACTTCATAGGCTTTGCTCAACCATCTTCT
TCTTCTCTTCTTGGTCTCAACCACTTAACCCCATCTCAAATCAACCAAAT
CCAAGCACAAATCCAACACCAAAACTTTGTACAACAACAACAACATGTAC
AACAGCAACAGCAACAACAACAACGTTGTCTCAGTACTACTTTGAGTTTT
CTGAGTCCAAAATCGATCCCTATGAAGCACGTGGGTGGAAATGCTTCAAA
ACCCACGAAGCTATACAGAGGGGTGAGACAAAGGCACTGGGGAAAATGGG
TAGCTGAGATAAGACTTCCAAAGAACCGTACAAGGCTTTGGCTTGGTACC
TTTGATACAGCAGAAGAAGCTGCTTTGGCTTATGATAAAGCTGCTTATAA
GCTTCGTGGTGACTTTGCTAGGCTTAATTTCCCGAATTTGAAGCACCAAG
GTTCGGTTATTGGTGGTGAGTTTGGTGAGTTTAAGCCTCTTCCTTCCTCT
GTTGATGCTAAGCTTCAAGCTATTTGTGAAGGTTTGGCTGAGATGCAGAA
ACAGGGGAAGGTGGAGAAGCCTAAGAAGACGCCGAAGTCGAAAGCTGCTT
CCAAGGTGGCTCCTAAGGAAACTGTTGATGATTTGAAGAAGGGTTCAGAT
GAGTGTTGTAAGGTTGAAGAAGTTTCGGTGATAACTGAGAGTGAAGGTTC
TGAAGGTTCTTCACCACTTTCAGATCTAACTTTTGGTGATGTTGGTGAGC
CACAGTGGGAGGGTGATTCAGAAAATTTTAATTTGCTGAAGTACCCTTCT
TATGAGATTGATTGGGATTCTCTGTGA
>MaERF027
ATGGTTAAGTCAGAGAATAACAAGATCAAAAAAGAAGCATCAAATTCAAT
CTCAGATAGTAGCAAGAAGAAATACAAAGGAGTAAGAATGAGGAGTTGGG
GTTCATGGGTTTCAGAGATTAGAGCACCAAATCAAAAAACAAGAATATGG
TTAGGTTCTTATTCAACTCCTGAAGCAGCTGCTAGAGCTTATGATGCTGC
ACTTTTATGCCTTAAAGGTTCATCATCAGCATCAAATCTCAATTTCCCTT
TAACAACCTCTTCTTCACATTACAATATTCCTCAAGATATCATGTCTCCA
AAATCAATTCAAAGAGTTGCTGCAGCTGCTGCAAATAGTTTCATTGATAA
TAATAATAATGTTAATGTCAATGTCAACAATGTTAACACTCCCTCTTCAT
CATCATCATTGGTATCATCTCCATCATCAATGGTTTCTTCTGATGATGTT
TCTTCACTTATGTCATCTTTTGATCAAGCTAATAATGAATCAATGACTAT
GATGGAAACTTGGTATGGATTAGAAGGTTTACAATCTCCTAAATATGTTG
ATCAAATGTTGTTAAGTGCTTCTTTCTTTGATATTGATTCATCATCACAT

TTGCTTGGTGATGATCTTTATGAAGAAAGTGACATTCGTTTGTGGAACTT
CAGCTGA
>MaERF028
ATGGCAAGACCACAACAACGTTATAGAGGTGTACGTCAAAGACATTGGGG
CTCTTGGGTTTCTGAAATTCGTCACCCTTTATTGAAGACAAGAATATGGT
TAGGAACATTTGAAACAGCAGAAGATGCAGCAAGAGCATATGATGAAGCT
GCAAGGTTAATGTGTGGACCAAAAGCACGCACCAATTTCCCATACAATCC
AAATGGACCACAATCTTCTTCATCTAAGCTTCTTTCAGCAACTTTAACTG
CAAAGCTAAATAAGTGTCACATGGCTTCTCTTTCTCTTCAAATGACCAAA
CAACAAGAAGCACCACAAAAAGAGCCACAAAAAGTTTCAACACAAAGTTT
CAATTCTTCCATTAATAATAACACTTTTGTCTATGGCAATGACATTGGTG
GAACAAGTGGTGAAACTAGTTTAATTAGATGGCTTGATGAAGGAAATAAT
TGTGTTGGTTTTGAAGGTCAAGTTGAAGTTTCTCATCAACAATTTCAACC
GGTTCTTGAAGAAGATCACATTGAACAAATGATTCAAGAGTTGCTTGATT
ATGGTTCAATTGAGCTTTGTTCTGTTGATTCATCTTAG
>MaERF029
ATGAACATTGAAAACTTGTCACACGACCCCACAACACCAACAACCACTAC
CTTAAACTTTCAACAAACATCTCTCGAAGGAATCGCTGCTATTGTTGGAG
AACAAATCCTCTACGGTTCCCACAACCCTAAAACAACATCATCAACAACT
TGTGTTTCAAACCCTAAAACCAAAACAAAACCAACAAAAAACAACACAAA
CCCATCAAACAAGAACTATAGAGGAGTTAGAAAAAGACCATGGGGAAGAT
ATTCAGCTGAAATACGTGATAGAATCGGTCGGTGTCGTCATTGGTTAGGA
ACGTTCGATACGGCGGAAGAGGCGGCGCGTGCTTATGACGCGGCAGCTAG
AAGATTGAGAGGTTCTAAGGCAAGAACTAATTTTAAGATACCGTTGGTTT
TGCCAATACCGTTGTCACCATCAACCTCATCTTCTTCGTCCTCGGATATG
AAAGGTAAGAACAAGAAAAGTTTACATAGGAAGTGTTGTGTTGTTAGTTC
TCTTGGAGAGTTGTTTAGTGGTGTGCCTGAAGTTAGAAGAGAAAGAGAAA
GGGATAATAATGATAATGGAAATGGAAATGTAGTTTTTGGTGGTAGAAGC
ACTGGAATGGTTATGTAG
>MaERF030
ATGGTTTCTGCTCTCTCTCAAGTCATTGGAACATCTACCAATAATAATAA
CCCACACATGGCACAATCAACCTCAACTACTATGGTCAATGAAGAATCTC
AACCACCTCAACCTCTTCTTGTTCAAGAGACCAAAAACAAACCACACTAT
AGAGGAGTGAGACAGAGACCATGGGGAAAATGGGCAGCTGAAATTCGTGA
CCCAAAAAAAGCAGCTAGGGTTTGGTTAGGAACATTTGATACAGCTGAAG
ATGCAGCTCTTGCATATGACAAAGCAGCACTCAAATTCAAAGGCACAAAA
GCTAAACTTAATTTCCCTGAAAGAGTTGTTCAATGCAACACTTCTAGTGC
AATTCAACAAAGTGGTTCTGTTTCTAATTCTGTTGATCAACAAGTTTTTC
CAAACTTGTTTCAATATGCTCAGATTCTTTCTAGTGATGTTGAATTCCCT
TATTATGCATCTCATCTTTTCAATCAACAACAACATCAAGGAGATAGTCA
CCCACCAGGCCAGTAG
>MaERF031
ATGGAAAATGATGCTAATGAAAACTTCTTTAGCACATCCTCATCAACATC
ATCAACACCAACATCTCCTAACTCAAATTCAAATTCAAGCACAAGTATTC

ATGACACCAAAAATAGCCCACAAAATGTAATGAAGAACAAGAAAAGAGAG
AGAAATGAAAATGACACAAAACACCCTACTTATAGAGGGGTAAGAATGAG
AGCATGGGGAAAATTTGTATCTGAAATCCGTGAGCCAAGAAAGAAATCAA
GAATTTGGCTTGGAACATACCCTACAGCTGAAATGGCAGCTAGAGCTCAT
GATGTAGCTGCTTTAGCTATAAAGGGTCATTCAGCTTACCTCAATTTCCC
TAATTTGGCTCAAAATCTTCCAAGGCCTTCAACTACATCACCTAAAGACA
TTCAAATTGCAGCTGCAAAAGCAGCAGCCACTGTCTATGTTGAGATTGAG
AATTGCCAAGGTGAAAATGATCAACAACAAAGCCAAGAAGATCAAGATCA
AGATCAAGTTTCATCTTCAACTCTTTCTTCCATAGATAATGTTCAAGAGT
CTAATTCAAGTTCTCCATCTACTACTATTACAAATGATGATATTGATGAC
ACACTCTTTGATCTGCCGGATCTTTTTCCTGATGGAAAAAAATGGAATTCT
TTCTTATTCTTCTTCTTGGCATTTATGTGCCATTGATAGTGGACTTCGTC
TCGAGGAGCAATTTTCGTCGTCTTGGGAGTACTACTAG
>MaERF032
ATGAAGATTGATACTGTTTCTGGACAAAGAAAATCACGCAAGAGACGCAG
TGGTGGAAGAACCGACTCAGTTGAAGACACTCTTGAGAAGTGGAAGAACT
ACAACAGACAGCAACAACAAAGACTTGGTTGTGGAGATAATGGAGCTGAA
AAGATTCATAAAGTTCCTGCGAAAGGTTCAAGAAAAGGGTGCATGAGAGG
TAAAGGTGGTCCTCAGAATTCAGATTGCAATTTCAGAGGAGTTAGACAGA
GGATTTGGGGTAAATGGGTTGCTGAGATTCGCGAACCTATCAATGGTAAA
CATGTTGGTGAGAAAGCAAATAGGCTTTGGCTTGGTACTTTCACTACAGC
ACATGATGCTGCTCTTGCTTATGATAAAGCAGCTAAGGCTATGTACGGAC
CTAGTGCTCGTTTGAACTTCCCTGATGGATCACCATCATCATCAAGTGGT
GGTTCTGCAGATTCTTTGAATGGCGAGGAAGATATTGGAAAAGCTGAAGA
GTTGGAGGCTAATCTTCATCAGTCTGATGAAGAGAATAAGATTCTTTCTA
AAGATTTTGTTGCTGATGACGACTCAGTAGAGGAATCGAAGGAAGTGATA
ATTGGTACTATCCAATGTCAAACAAATAAAAGATGTAAGAAAATGGTTCG
TCAAGGGTCTTACAAGAATGTTAAATCTGAAAAACCTGGCAAAAACGAAC
AACTAGAGGGTGAATTAGAGAAGATTCTAGCGAATTCTGGCATGGGTGGA
GGGTGTAATCATGTGCATAAGGAGCCTATGGATACAGGAATGAACTCAAG
AGCTGATTGTAGATCTTCTGATATAGCAGAAAATGGGATTCTGGTGCAGA
GTGAAGAAACAATAAGAGGATCAGTGGAGGATTTGAAATCCTTTGAGTTG
AGCTGCAGCAACCATTTCCTTGGAAACCTGCACAATATGTTCCCAGATAG
CGATCCAAGACCAAATTCTGAGCGTTGTAACATCAAAACCGAGGCTTATC
TTGCAAAGAAACATAACAAGGAAGTAAGTGGAGATTTCTTAAGCCACGCT
CAGCCACAAAATGAGCAAAATAAAAATGGATATTTTGATGAGATCAAAAC
TGAGCTTAAGGGATTGGAATACAAGCTGAGAGGCCAATCTATCGATTGCA
AGAATGATGAGGCACACGTAGTACCTTACATGCAGGGTATTGATCTGTTT
GGTGGTGGCAGTGTTGGACCAATTGAAAGAATGCCCCAAATTGAAGCTTT
GAACAATACTAATAAAGGCACTAAATTAAAAGAGAGAGGAAATAATGGAA
GTGCACTGCATGGATTTAGTGCAGGACAAAGTAGGAAGCTGAGTGATCTT
TCTCAACAGCTGCAGAAATTGGGTGGTTACTTGCCTGAAAATTGGAATAA
TATGCAGTTTGCAGATCTTGAAGTTGGTTATGATTATAGTTTCTTAAAAC
CTGATTATGATTTTGGCTTATTAGAAGAGAAGAAGTTACTAGATATATGT

TTTTCACATATAGGATCTTGA
>MaERF033
ATGAGTCCTACAAAAGGAAAGAAGAAGCAAACAACAAGCGGCGAAACAAC
GACACAGGAGCAACAACAAACAGCATGGGGAGGAAGATACCTAGGAGTAC
GAAGAAGACCATGGGGAAGATACGCAGCAGAGATACGTGATCCTTCTACA
AAAGAAAGACACTGGTTAGGAACATTCGACACTGCTGAAGAAGCTGCTTT
AGCTTACGACAGAGCCGCACGTGGCATGCGTGGTTCACGTGCCAGAACCA
ATTTTGTTTACCATGATACCCCTCCTGGTTCTTCTGTTACTCCAATCCTC
TCCCCTGATCAACCACAACAACAACAACCTCAAAGTTTTTACGATCTTTC
CTTTGTTCTTCAACCTGAACCGGTTCCTATTCAACAGAACCCGGTTTTGT
CTCTTGAACCGGTTTCAATTATAACTAATTACGGTTACGGTGAGGGAAAT
AATAATAATAATAAATATAGTCTTCCTCATCAAATTCCTCTTGACACATA
TAATATTAATACTAATGGAAATTCCTATTTTAACCTTAGCAATACTAGTA
GCATTAACAACGACAGCGTTGAGCTTCCTCCATTGCCACCGGATATTACG
AGCTCCGCGTGTTATAGTCAGGGTGATTCAGTATTCAACGAAGGTGGTGA
CAATGGATATTATTATTCATCATGGGAGCAGAATGTAGGAGTGGATTGTT
ACAATAATCAAATGTTGGGTACAAATATGGGAGGTGCAGAGAATAATAAT
AATAATATTATGGCAGGTTCTGTTAATGGTACTTTTGATTTTGGAAGCTC
TTCATTTTTCTACTAA
>MaERF034
ATGGAAATTCAATTTCAGCAACCAAACATGCAGCATCAGAAAGCAGGAAT
TTCAGTAACCAACAAAGGAGGAAAATTCAAAGGAAGAAACAGAAATAGCA
ACAACACAAACAAATTTGTTGGTGTTAGACAAAGGCCATCAGGTAGATGG
GTAGCTGAGATCAAAGACACAACTCAGAAGATAAGGATGTGGCTTGGAAC
ATTTGAGACTGCTGAGGAAGCAGCTAGAGCTTATGATGAAGCCGCGTGCC
TTCTTCGAGGATCGAACACTCGAACCAATTTCATCACTCATGTCTCATTG
GATTCTCCTCTTGCTTCTAGGATAAGAAATCTTCTTAATAACAGAAAAGG
CGACAAAAAAAAAGGAAGAGATTGTTGTTGCTTCTGCTCCAAGTAACAGCA
ACACTACCATTAGTAGTACTAGTACAATTACAAGCAATGATGATAACAAG
GAGAGCACTCTTTCAACTTGTGCTACTCAAAATACAGAACTTTTTGAGGA
TGCTTATAAACCAGATTTGAGCAATTGCAAGGAAGTTTTTGAGTTAGGTT
CACAATCCAATGTTTCATGTGGTTTTGGACCTTTCTTTGATCATTTTTCT
TTTACTCAATTATTGGATATGGCGAAGAATGATGGCGTAACCGATACATC
TAGCTTGGAGCTTTCAGAATTTGAAAGAATGAAGGTTGAAAGACAGATAT
CAGCTTCACTTTATGCTATTAATGGAGTGCATGAGTACATGGAAACTGTT
CAAGAGTCAAATGAAGCCTTATGGGATCTTCCACCCTTGTGCTCATTATT
CTGTTAA
>MaERF035
ATGGCTTCATCTTCCTCCGATCCCGGTGGCGGCAAGTCTGTTGAAACCTC
AGAAGCTGTGGCGGCGGCGAATGATCAGCTTTTACTGTACAGAGGATTGA
AGAAAGCAAAGAAAGAAAGAGGTTGTACTGCTAAAGAACGAATCAGTAAA
ATGCCTCCTTGTGCTGCTGGTAAACGCAGCTCCATCTACCGTGGTGTCAC
CAGGCATAGATGGACTGGTCGTTATGAAGCGCATCTTTGGGATAAAAGTA
CATGGAACCAAAATCAAAATAAGAAAGGAAAACAAGTTTACTTGGGGGCA

TATGATGATGAGGAAGCAGCAGCTAGAGCGTATGACCTTGCTGCTCTGAA
ATATTGGGGTCCTGGGACTCTCATTAACTTCCCAGTGACTGATTATACAA
GAGATCTCGAAGAAATGCAGAACGTTTCAAGAGAAGAATATCTTGCATCT
TTGCGACGGAAGAGCAGTGGTTTTTCAAGAGGTCTATCAAAATATCGTGG
ACTCTCCAGTCGATGGGGGCCATCATATGGTCGAATGGCTGGATCTGATT
ACTTCAGTAGTATACATTATGGGGATAATTCAGCTGCGGAAAGTGAATAT
GTTAGTGGTTTCTGCGTAGAAAGAAAGATTGATTTAACAAATCACATCAA
ATGGTGGGGATCTAATAAGAATCGACAACCTGATGCTGGAACAAGATTAT
CAGAAGAAAAGAAACATGGTTTTGCTGGGGATATTTACAGTGAACTTAAA
ACATTGGAACAGAAAGTCCAACCAACAGAACCGTACAAGATGCCAGAGTT
AGGACCGCTGCACAATGAGAAAAAACATAAAAGTTCTTCAATCTCTGCCC
TAAGTATATTGTCTCAGTCTGCTGCTTACAAGAGCATGCAAGAGAAAGCG
GCAAAAAGACAGGAAAATAGCACTGATAATGATGAGAATGAAAACAAAAA
TATAGTCAATGAGTTGGATCGTGGGAAGGCAGTCGAGAAACCCTCAAATC
ATGATGGTAGCAATGATCAACTTGACATTGCAATGGGAATGAGTGGGGCG
TTGTCTCTTCAAAGAAATGTTTACCCGTTGACATCATTTTTGTCTGCACC
ACTTTTGACAGCCTACAACACTGTTGATCCAATGGTAGATCCTGTTCTCT
GGACATCTCTCATTCCCATGCTTCCTGCTGGCCTTTCTCGTACATCTGAG
GTTACAAAGACGGAGCCGGATTCAACCTACACTATGTTTCAGCCACAGGA
GTGA
>MaERF036
ATGATGGATACATACAAGAAATCACCCTTGAAGCCATGGAAGAAAGGACC
AACTAGAGGAAAAGGTGGCCCCCAAAATGCTTCCTGCGAGTATCGAGGCG
TTCGTCAAAGAACGTGGGGTAAATGGGTTGCCGAAATAAGAGAGCCAAAG
AAAAGAACTAGGCTCTGGCTTGGTTCTTTCGCCACTGCTGAAGAAGCTGC
TATGGCTTATGATGAGGCTGCAAGGAGGCTCTATGGACCAGATGCATACC
TTAATCTTCCACACTTGCAAACACACTCCAATTCAACTATCAAAACTGGA
AAGTTCAAGTGGTTGCCTTCAAAGAATTTCATTTCAATGTTTCCTTCTTG
TGGATTACTCAATGTAAATGCTCAGCCTAGCGTTCATTTGATCCATCAGA
GGCTACAAGAGTTTAAGCAGAATGCAGTTGTTACAAGTCAATCATCACTT
TCTAGTTCATCAAACGATCCAAAGGCAGAAGAAATACAGAAAGTAGACAG
CAAGAAGAATCATGCAGAAGATCCTTCGCCAAAGGGAAAAGATGTTCAAA
CATCGGCGAATAAGATGATTGGAGATCTTCAGGAGGAGAAACCACAGATA
GACCTAAATGAGTTTCTTCAACAGCTGGGAATACTGAAAGAAGAAACACA
CTCAGAACAAACAGAGAGTTCAGGAAGTTCAACAGTGCATGAAGCTGTGC
CAAGAGATGATAATGATCAGTTGGGAATATTTTCTGACATGAGTGTTAAT
TGGGAGACATTGATCGAGATGCATGAATTTGCAGGTATTCAGGAATCAGA
AGCCACCCATCTTGAAGCATATGACCCGAATGACCACCTTAATTTCTCAA
CTTCCATTTGGAACTTTTAA
>MaERF037
ATGGAAGATGAGTTAAAGGAAAAGGTGAAACAAGATGAGTTACCAAAGCA
AATCAAGTATAGAGGAGTAAGGATAAGGCCATGGGGAAAATTTGCGGCTG
AGATTCGAGATTCGGCTAGGCATGGTGCTCGAGTTTGGCTTGGTACATTT
AACACTGCTGAGGAAGCTGCAAGAGCTTATGATCGTGCTGCTTTTGCAAT

GAGAGGTTCTTCTGCTATTCTCAACTTTCCTCATGAACATTTTCCATGCA
ATGTGGTTTATAACCCTTCTAAGCCCTCTTCTTCATCCAATTCAACCTCA
TCTTCATCTTCAAATGTTAAATCAGAACTTGGTGAACAAGTTATAGAGTT
TGAGTATTTGGATGAAAAATTGTTAGAGGAACTCTTGGATTTTGAGAGTG
TCATCAACCAGGATTTGTTCAAGTAG
>MaERF038
ATGGACAATCAACCAACACCATTCATTGAAGATGACGTAACCGCCGCTGA
TAGCCGGAGAGGTGGAACGCGCCACCCTGTCTACCGCGGTGTCCGGAAAC
GGCGTTGGGGAAAATGGGTTTCAGAAATTCGTGAACCTAAGAAGAAATCA
CGTATATGGCTAGGATCATTTCCGGTGCCGGAGATGGCTGCGAAAGCATA
CGACGTCGCAGTTTATTGCCTGAAAGGCCAAAACGCGCAACTAAACTTCC
CCGACGAGGTGGAGAATCTACCGCTTCCGGCCACATATACAGCCAGAGAC
ATTCAAGCTGCTGCAGCCAAGGCGGCGAGTATGATGAAGGCTTCACTGGA
TTGCATTGTTTCTGATGGAGATAGCGGTGGTGATGATTTTTGGGGTGAGA
TTGAGTTGCCGGAGCTTATGGAGAGTGATTGTTGCTGGAGTTCTCCGAGT
GGATCATCGTGGACTTGCTCCGGTGATATCACGGCGGCGTGGCAGGAAGT
TGAGGAGCTTTTGGTGCAGCAACCTCATGTCGTGTCTATAATGAGTACTG
TTAATAATGTTTCTTAA
>MaERF039
ATGCCAGAAAACAACAATACTACTTATGCATCATCATCATCATCATCAAG
CAAAGTTTCGACTGGGCGTCACCCGGTGTACCGAGGAGTGAGGCGTAGGA
ATAACGGAAAATGGGTATCTGAAATTCGCGAACCGAAAAAACCTAACAGA
ATTTGGTTAGGGACATTTCCAACACCTGAAATGGCAGCTATAGCATATGA
TGTAGCTGCTCTTGCTCTTAAGGGTAAAAATGCTGAGTTGAATTTCCCTA
ATTCTTCTTCTTCTTTTCCTGTTCCTGCTTCTTCATCTCCTCGCGATATT
CAAATGGCTGCAGCTAGTGCTGCAGCTGCTGTAGGTGCAGCCAATGATGC
ACTCATCAGTAATATTAATGAGGGAAATAACCATAATGTTTCAGTGGAAC
CACAAGAGTTTTTCTCAGGTGGCAATGAGAACAATTATAATGTTAATGAG
TTTGTTGATGAGGATTTGATATTTGATATGCCTAATGTTTTGGTTAATAT
GGCTGAAGGAATGCTACTTAGTCCTCCTCGTTTTGACTTTGTCAGTAATG
ACTATGAGGATGCACCAGAAAACATTTCATGTGATGATCAAAACCTTTGG
AGTTATCCTTATTTCCCATAA
>MaERF040
ATGAAACAAACAAGAAACTGTTTCTCACCTTCAATTTCAAACTCTTCAAA
ACCTAAAAAAACAGAGACAAAGAAACAGAGCAAGGTGAAGAAAAACAAAG
ACAGCAACAACCATCCGGTTTACCACGGCGTTCGAATGCGAAGTTGGGGA
AAATGGGTATCAGAAATCCGCGAACCTCGCAAGAAATCAAGAATCTGGCT
TGGAACTTACGCTTCCCCTGAAATGGCAGCTAGAGCACACGATGTTGCTG
CTTTGAGCATAAAGGGTCACTCACCAATTCTTAATTTCCCCGAAATAGCT
CACGTGCTTCCTAGGCCAGTCACGTTGAATCCACGTGATATTCAAGCTGC
AGCTGCAGAAGCTGCTGCCATGGTGGAATTTGATTCTCAGATATTGCACT
CAACAATCTCACTACTAAACTCTGAAGTCTCTGACTCACCTTCATATTCA
TCAGAGTCTTCTGAGCTGAGTGAGATTGTTGAACTTCCTAATATAGAAGA
AAATTTTGACTCGATTGAGTTGAGAAGTGAGTTTATGTTGATTGACTCAT

TGGAAAGTTGGGTCTATCCGCCATTAGAGAGTTTCTGTGACATGGTTGAA
GAACAGAGTTTTTTGTTATAA
>MaERF041
ATGGCTGCAGCTAAGAACAGTGGCAAATTCAACCAAGATTTTGGTGATGG
AACTCATAACAATCACAACAAGGTTACAAATATTGGTGAAAGCCAAGTTA
ATTGGGAGATTGAAAAAGGAAAAAGTGTTGAAATAAGCTCTTCTCAAAGA
CATCATTGGAAGCCAGTTTTTGATGAAGCTTCAAGTATGTCACATAATAG
ACCTCACAAGAAAAGCAAAAGTCCTCAACGTGAAAATCAAAACCAAAATC
AGTTTCAACCTTTCTCTGTTCCTAATTCAGCTTCTTCTTCTTCAAGACTT
GTTTTTCCTTTTGCTTTTGATCATAATTCACAACAATTTGGTGCCAACAA
CAATTTACCTTTTCACCCTCAACAACCTATTCAAACAACACAAAACCAAC
CACAACAAATGATATCTTTTGGTTCACAATCACAACAACAGAACAATCTT
GTTCCATATCCACCAATGTTGTCTCAACAACAACATCAACAACAGATTCT
TCAATATTGGAGTGATGCATTGAATCTTAGTCCAAGGGGAAGAATGTTGA
TGATGATGAACAATAACAACAATAGGTATTTGGGAGGGCAATATGGTAAT
AATAATGGACCAATGTTTAGGCCTCAAGTTCAACCTATAAGCACTACAAA
ACTCTATAGAGGAGTGAGACAAAGACATTGGGGAAAATGGGTTGCTGAAA
TTCGTCTACCTCGAAATAGAACTCGTCTTTGGTTAGGAACATTTGATACT
GCAGAAGATGCTGCTTTAGCCTATGATCGCGAAGCGTTTAAGTTAAGAGG
TGAAAATGCAAGGCTCAATTTTCCTGACTTGTTTCTCAACAAAGAAAAAG
ATAAAGAAAAAGAAGAAGCACCTTCATCACCACCTCCAACTACAACAACG
ACAAATTCAACGGTTACTTCACCTTCAAGTAACACTGCGACAAAGCAACC
TGAGCCTCCTCCGATGCAGACACTTCCAATGGAAGAGTCTAATGAGAATG
ACTCTGGAATTGGATCAAGTGACGCGACAGTGAGTGAAGGCGGAGAAGGA
GTATCAGTTTCGCAGTCTCAAGAATTGGTTTGGAGTGAAATGTCAGCATG
GTTTAATGCTATTCCAGCTGCTTGGGGACCAGGTAGTCCTGTTTGGGATG
ATTTGGATACAAACAATAACCTTTTTTCGCAGTCGCAAATTCCATTTTCC
AATCTCAATCAACAATCAGAATACAATGATTTTGATTCTCAGATGGAGTC
AGGTTCTTCTTCCATTAGGCCTTTCTTATGGAATAATGATCAAAATTAA
>MaERF042
ATGTTGGCGAAGGCTTGTGAAAAAGGGGATGGATCTAAGTCCCTGGGTAA
GATACTGGCAAGATGGAGAGAATATAATGCCCAACTTGAAACCGGCAATG
ATGCGGATAAGCCTGTTCGAAAAGTTGCTGCCAAAGGATCGAAGAAAGGG
TGTATGAAAGGTAAAGGAGGGCCGGAAAACTCACGGTGTAATTATAGAGG
TGTGAGACAAAGGACTTGGGGGAAATGGGTTGCTGAAATCCGTGAGCCGA
ATCGAGGGAGTAGGCTATGGCTAGGTACTTTTGCCACTGCCATTGGTGCT
GCTCTTGCTTATGATGAGGCAGCCAGGGCAATGTATGGTTCTCGTGCCCG
TCTCAACTTTCCTAATGTTTCAGTTGGTAGATTCTCTGAGGAATCTTCAA
ATGATTCTCTAGATGCAAATCACTTCGGTTCCTCGTTGGGAGTATCAACA
AATACCGAGTCCATGATAATACCAGATAACTCAGGCATAGGAGTAGACGA
CGGTAATAATATGGAACCACCGATTTCCTTATGCTTAAGTGTAAAGCAGG
AGAATGAGAAAGGTGAGCCGAGGATAAATTAA
>MaERF043
ATGGCTCCAAGAGACAACAGAGTCACCACCACCACCACCGGGCCCAACCT

AACAGTTCAGGCCCATGCCCAGGCCCAGAGAGAGATTCGTTACAGAGGCG
TTAGAAAACGTCCATGGGGTCGTTACGCTGCTGAGATCCGTGACCCGGGT
AAAAAAACACGCGTCTGGCTCGGAACATTTGACACCGCTGAAGAAGCGGC
GCGTGCTTACGACACAGCGGCACGTGAGTTTCGCGGAACCAAAGCGAAAA
CTAATTTTCCAACGCCGTTGGAGATTATCATCAACCGTAGCCCAAGTCAG
TGCAGCACTCTTGAATCACCATCACCACCGCCGCTTGATTTAACTCTTAC
CCCATTCTCCTCCTCCGCCGGTGGTGTTTCCAGCGGTGTTACCATGGCTT
TTCCGGTGGCGCGTCCTGTTTTGTTTTTTGACGCTTTCGCACGCGCTGAA
ACTGCGTTAAGCGTTGGTCGTCGTGAGATTTGTGGATTTGAACGTCCTAT
GGCTGATTTTCGACGCGCCGCCGTGCAGAGTGATTCTGGTTCATCTTCTT
CTGTTGTTGATTATGAAGGTGTGCCACGTCAGAGAGTGTTGGATCTTGAT
CTTAATGTTCCTCCTCCACCTGAAGTTGCTTGA
>MaERF044
ATGGTGAAATCAAATGTGGGAAAAAATTGCGGCGGAAAGAAGTGATTGTTC
TTCATTGTTTCGAGGAGTAAGGAAGAGGAAGTGGGGGAAATATGTGTCTG
AAATAAGACTACCAAACAGCCGCCAGAGAATATGGTTAGGTTCTTATGAT
TCTGCTGAAAAGGCGGCGCGTGCATTCGACGCGGCTATGTTTTGCTTACG
TGGCAGTGGTGCTAAATTTAATTTCCCTAATAATCCTCCGGACATTGCCG
GAGGAAGGTTCATGACACATTCTGAAATTAAGGCTGCTGCAACCCGTTTT
GCAAATTCGGGTCAACCCGATAATGAGTATTCGGGTTGGTCCGTTAATAC
TCCGACTGAAACACCATCTTCGTCCGAGGGAACGACGTCATCATTGTTGC
CTACGGAGTCCCCATCTCCGGCACTCTCTGAGGCGACAGTTCAGGGAGAT
TATGATTTAAAGCAAAACGGGTTATTTTCGGATATGTTTTCTGAAATCGG
ATCAGGTTACTCTATGTTTCCGGGTTTTGATGATTTTTGCGGTGATTTTT
ACGTGCCAGAGTTGACAAATTTTGATTATGAAGACGAGAATATGGATGAG
TTAGTAATTCAAGACCTGTTCTTGTGGAATTTCTAA
>MaERF045
ATGGTGAAATCAAATGCGGCGGCGGAAAGAAGTGATTGTTCTTCATCATT
GTACAGAGGAGTAAGGAAGAGGAAGTGGGGGAAATATGTCTCCGAAATAA
GACTACCAAACAGCCGCCAAAGAATATGGTTAGGTTCTTATGACTCCGCC
GTTAAGGCGGCGCGTGCATTCGACGCGGCTATGTTTTGCTTACGTGGCAG
TGGTGCTAAATTTAATTTCCCTAATGATCCTCCGGACATTGCTGGAGGAA
GGTCCATGACACATTCTGAGATTAAGGCTGCTGCAGCAAGTTTTGCTAAT
TCAGGTAGACCTGTTAACACTCCGGTTGAAACACCGTCTTCATCGGAGGG
GACGACAACTGCATTGTTGCCTATCGAGTTGCCGTCTCCTGCTTTATCCG
ATGGGGCGGTTCAGACAGATAGTGATATAACAGAAAATGGATTGTTTAAT
GACCTCTTTTCCGGTTACTCTATGTTTCCGGGTGTTGATGATTTTTGTGG
CGATTTTTACGTGCCGGAGTTGACAAATTTTGATTATGAAGAAGAGAACA
TGGATGGTTTGGTAATACAGGATTCATTCTTATGGAACTTCTAA
>MaERF046
ATGGTGAAGTCGAATGTGGAAAAACCTACCGTGGAACAAAGTGATTCATC
ATTGTACCGTGGAGTACGGAAGAGGAAGTGGGGGAAATATGTGTCGGAAA
TAAGACTACCAAACAGCCGCCAGAGAATATGGTTAGGTTCTTATGACTCC
GCAGAGAAGGCGGCGCGTGCATTCGACGCGGCGATGTTTTGCTTACGTGG

TGGTGGTGCTAATTTTAATTTCCCTAATGATCCATCAAACATTGTCGGAG
GAAGGTCCATGACTCCTTCTCAAATTCAAGCCGCTGCTGCCCGTTGTGCA
AATTCAGATATCCATCATGAGAATTCGGGTCGACCCGGTAATAACGCGGT
GGAATATGAGTCGTCATCGGAGGGGACTGCATTGTTGCCTATGGAATCTG
AAGCACCGTCTCCGGCAATGTCAGATGCAACGGTTCAGACAGATTGTGAA
TCCAAGCAAATCGGGTTGTTATCTGATTTGTTTGTAGTAAATGGGTCGGG
TAAGTTTGAACCGGATTACTCGGTTTTTTTGGGTTTTGATGAATTTGGTG
GAGATTTCTTTGTGCCAGATCTGCCAATTTATGATGACTATGGAGCAGAG
AACTTGGATGGGTTGATAATTCATGATTCGTTTTTGTGGAATTTCTAA
>MaERF047
ATGTGTGGAGGTGCTATCATTTCTGATTTCATTGGTGTCAAGCGTGACCG
TGACCTATGGTTTGAGCTTGATCCTTCTGTTGATCTACTTGGTCTTGGTG
GTGGTGCTGCTGCTTTTAACACCCCCACTTCTAAAGAGCTACCACCCCTT
AGCTTTGAGCAATTCTTCTCATATGACAAAAAAGTGGTAGCATGTGACAA
TGTTGAGAAGAAAGAAAGCTTGGAAACTGTAGAGAAGGGTAAGAAGAGCA
CTGGAGGAAAAAGAGCTCGCAAGAATGTCTACAGAGGAATCAGGCAGAGG
CCATGGGGCAAGTGGGCTGCAGAAATAAGGGACCCACAACAAGGTGTTCG
TGTTTGGCTTGGCACTTTCTCAACAGCTGAAGAAGCAGCTAGGGCCTATG
ATGCCGCCGCCAAGCGCATCCGCGGCGACAAGGCAAAGCTCAATTTCCCC
GACACTCCTGTCGTTGCTGCTCCGGATGCTGCTGCCCCTGTCACCGCTCC
TCCAGCAAAGAAGCGGTGTGTCAGCCCTGATCAAGCCAGCTCCCAGGCTA
GCTCTGAGTCAGCTTGGTCTGGGTTTGGGCTTGATGAGGAAATGGAAATC
AAACAGAAAATGTCTGACCTTGAATGGATCCTTGGATTGGATAGTGACAT
TCCTCAGGCCCAAGCCCCGGCCCAAGTTGAGGTTCAGTCTGTGCCGGAGC
CCGAGCCTTTCACCTTGCCTGCTGATTGGGACAACAGCTTCATGGACTTT
TGGACTTGTGAAGACATGGTTGACCCCATCCGTCACCTGCTTTACTAG
>MaERF048
ATGGCGCCGAAAGTTAAAAACGGTGGCGTTAACGTGAACAATAACGGAAA
CGTTAACGGCGGCGGCGTTAAGGAGGCGCATTTTAGAGGAGTAAGAAAGA
GACCATGGGGAAGATACGCCGCCGAGATAAGAGATCCAGGAAAGAAAAGC
CGCGTTTGGCTCGGAACCTTCGACACGGCGGAGGAAGCGGCGAGAGCTTA
CGACGCCGCCGCACGTGAATTCCGCGGTCCAAAGGCGAAAACAAACTTCC
CCTTCCCTATTCAGAGTCCTAGCCAAAGCAGCACCGTTGAATCCTCCAGC
CGTGACCGTGATCGTGACGGCGCCGCCGCTGATTCTTTACCGCTTGATCT
CAACTTAGCTCCTGCTTTCCAGCACCGGTTAACTCCGATGCCGGTGGTTA
ACCAGGCGTTTTGTTTCGACGCTGTTTTACGCGCCGGGATGGTGAATTCA
CGTCCTTGTTATGGTTATGGATTTGAATATAATCCTCTTAAGGAGGCGAT
TGGATTCCACGCGACGATTGGTGCGCAGAGTGATTCAGATTCTTCTTCGG
TGATTGATCTGAACCACCATGGTAGAAGCTTCGATCTCGATCTTAACTAC
CCTCCGTTGGAAGATATGCAATAA
>MaERF049
ATGGCTTCTTCATCTTCGAATCGTGAAGGTCACTACAGAGGTGTTAGGAA
GCGTCCATGGGGACGCTACGCTGCTGAGATTCGCGATCCGTGGAAGAAGA
CACGTGTTTGGTTGGGTACATTTGATACTCCTGAAGAAGCTGCTCTTGCT

TACGACGGCGCCGCTCGTTCTCTCCGTGGAGCCAAAGCAAAAACTAACTT
TCCTCCGGCGCCGGTTCCCGGCGGGATTTGCTTGGATCTCAACGCTCCTT
CCTCTGATCTCCGTTGGCCATCTATTCCGTCGCGTGGCCGTGTGCTCACT
GAGTTTCTTCAAACTGGTGTTCTGAAAGATTTCAACGCTCCTCCACCTCC
ACCCATGGGTTCGGTGGTTGGACAAAGGAATAATGAAGTTACTCTCGCCG
TCGCCGGCAGAACTATGGTGGCTGAGAACTCTCCTACGGCGGCTTATTTG
GGACTTGTTCGACGTGGGATTCCGATTGATCTGAATGAGCCTCCGCCGGT
GTGGCTTTGA
>MaERF050
ATGTCAACCTCTAGAACCTCAAACAATTCCCTTAAAGGACATAATCATCA
TCACAATCAAATGCAAATAAATTTATCTCTTCTTCAACGAAACACATCGG
TTTGTGGCGAAAAAAGAGGAAGAAAAAAGCAAACAGAACCAGGTAAGTTT
CTTGGTGTAAGGAGAAGACCTTGGGGTAGATATGCAGCTGAAATTAGAGA
TCCTGCTACTAAAGAAAGACATTGGTTAGGAACATTTGATACTGCTCAAG
AAGCTGCTTTTGCTTATGATAGAGCTGCTCTTTCCATCAAAGGTAGCAAT
GCTAGAACAAATTTCATATATCCTTCTTCTTCCGACGACGATGATGCTAA
TTTTAACAATGTTGTTGATACTCCTTTGGATGTTCAAAAAACACAACCAA
TTACCAATCAAAATAGTCTCTCTCAGCTTGATACTAATTCACATTCCAAC
ATTGAAAAACAAAATTATTCTCATTTGAACAATGACAATTTTTTATTCTC
TAATGATTATAATAACTCAGGCTATCTTGAAAGCATTGTTCCTGTTAGTT
GCTTCAAATCTATTTCCAACACCAATAATTCAAACTTTGATCATAAGAGT
AATGCAAGTGATTCAAGTGAACACCAAAATGTTTCAAACTTCATGCAGGG
TCAATCATTTTTTGACTACACTCCATTCTCTCAAGGAGCATTAAACATGG
ACACAAGTGAAACCAGTGAAGGATTATGGGATAGCAATTACAATGAACTT
TCAGCTATTTTTAACAAACCACTAAGGCATGAAAGTCCAAGCTATGGACT
AATGACAACTCAGGATGTTTCTTCAACGACCTATTCTTCTTTCGGCGATG
TTGACATGGGATACTCCCTCTTCTGA
>MaERF051
ATGATATCTATTTCTCTAATTCCTCATTTATGTGTTTATACCAACATGGA
GAATATTCACATTCACAATAGTAATAATCATCATGCAGCTACTGAAGGAT
CAATTCTTGAGAATGTATGGGCTAAAATTATAAGCAATGATGGAAGAACA
AATGTAAATGAAGCAGAATGTTCTAACACATGGGGAGAATTGCCTAATCT
TGACGAAAGAGACGGTTCGATGGAAATACTTCAAAGGCTTCCAAGTTTAG
GAAGATGGATATCAATGGGAGCAGATTATTGGGAAGAGCTTTTAGATGAT
ATTCTACCAATTACTGCAATAAGCAATACAGAAGATTTTTCATGCAACAA
AAAGATGGAAATTGAAAGTGTGAATAAATCAAATTCAAAGGAAAAAGTAG
AAGAAGATGGTGTGAGAAAAGAGAAAAAAAGAGTTGAAAAACATTATAGA
GGAGTAAGGAGAAGACCATGGGGTAAATATGCAGCTGAAATTAGAGACTC
ATCAAAGAAAGGTGCAAGAGTTTGGCTTGGTACATTTAATACAGCTGAAG
AAGCTGCTTTAGCTTATGATAAAGCTGCTTTGAGAATTAGAGGTTCAAAA
GCATGTCTTAATTTTCCAATTCAGACAGTTACAAATACAAACACTTTAGA
ATGTGGTAAAAAAGGAATTGAACATGATGTTACAAAGAGAGAAAGTTTAT
TGGATTCAAGAAAGAGAGGATTAAGGGACAGAGGAGAATTTGTTGATGTG
ATGAATACTGAACAACCTGCAACCAAAAAGATGGCAGGGTTGGAATTGGA

AGACATGTTAGAAAATGATGCTTTTGTATTTCAGGATCTAGGAATTGATT
ATTTGGATAGTTTGTTGTCTTCTTTTTGA
>MaERF052
ATGGAAAAACATATCAACATAGAAAGTGATGCTGATGACACTTCTAGCAC
ACCTTCTTTCTCTTCCTCCTCAACAACATCTACTTCATCAAATTCAGATA
CAAGCATCAAACAAGACATCAAAGCCTCTCCAAATGCAAAGGAGAGTAAG
AAAAGGCAGAGAAGTGAAAGTGAAAATAATAGCACACATCCAACTTATAG
AGGAGTAAGAATGAGAAATTGGGGAAAATTTGTGTGTGAAATTCGTGAGC
CAAAGAAAAAATCAAGAATATGGCTTGGAACATACCCTACAGCTGAAATG
GCAGCTAGAGCACATGATGTAGCTGCTTTAGCTATAAAGGGTCATTCAGC
ATACCTTAATTTCCCTGAATTGGCACAACAACTTCCAAGGCCAATAAGCA
CTTCTCCTAAAGACATTCAAGCTGCTGCTGCAAAAGCTGCAAACACTACA
TTTGTAGCTGAACCAAGACAAGAAGAACATGCCTCATCTTCAAGTTCAAC
TTTTACCAATGATGATGCTACATTGTTTGATTTGCCAGATCTTTTTCCTG
ATGATGGAAACAATGGACTTTGTTCTTATTCTTCAGAAAATTCTTCTTGG
TATTTACGTGCAGTTGATAGTGGATTCAGGCTTGAGGAACCATCTTTTTG
GGAGAAATACTAG
>MaERF053
ATGATGAATCTCGAAGCTACAACTCTTGAATCCGATCTAGCTTTTCTCGA
ATCAATTCAACGCTACCTCCTCGACGACCACCATGACTTCAACCCTCTCG
CCGCCGTAACCGCCGCCTCTCACGGCGTCGCTCCGGAGACCAACCGTACC
TCTCCATCTTCCCCAACCAACAGCGGCAGTAGTGATTCAATCTCCCACGC
GCTTCCGGAGTCGCGTGAATCACACGCGCCTCCTTGTTGGCAAAGATACA
AAGGAGTGAGGCGGAGGCCGTGGGGAAAGTTCGCAGCTGAAATTAGAGAT
CCGAATAAGAACAGTGCAAGAGTGTGGCTTGGGACTTATGAGTCGGCTGA
GGATGCGGCTTTGGCTTATGATCAAGCCGCTTTTAAAATGCGCGGCTCAA
AGGCTAATTTGAATTTTCCGCACTTGATTGGTTCCGATGTCTCGGAGCCG
ACGGTAGTCACAGCTAAGCGGCATGCACCGAAGTTGTCGTCTAGGTCGCC
GGTGCTTTTGGAGCCAAAAAGGAAAATGAGCCGAGTCAATGGCTATACGT
TAATATGA
>MaERF054
ATGAACACTCATGCTTGGAACACTAATGTGGCCACCCTTGATCACTTTGA
ATCTGCCACAAAGACACCGTTTCATGACACTTCAGCTCCGGCAGCACCGG
CGCCAATTTCCCGGAGTTCAAGCTTTCGGAGCATTTTCTTTGCGGAGAAT
TGGGCGGAGCTGCCGTTGAAGGAGGATGATACCGAAGACATGGTCATCTA
CGGGGCTTTGCGCGAAGCTGCTGCCACCACGGGCTGGTTCCCGGTTAGTA
ACAATGTGGTGAACAATGCTGATGTAGCTGCGAAAATAGAAGATCAAGGT
CAAAGTAGTACCACTTCTTCGTTGACGCGTGTTGCTCACGCGCCAACGAC
TAGTAAAAGGTTAGGCTACCGTGGAGTTAGGAGAAGGCCTTGGGGAAAGT
ATGCAGCGGAGATAAGGGATCCTAAAAGAAATGGCGCGAGGGTGTGGCTT
GGGACTTATGAAACAGCTGAGAATGCAGCTTTGGCTTATGATCGAGCTGC
GTTTAAAATACGTGGCTCGAAAGCTAAGTTGAATTTCCCTCATTTGATCG
ACTCAGATTACACTGGGTCGGTTAAGTTCACACGGAAAGAGCACTAG
>MaERF055

ATGTGTGGTGGAGCAATCATCTCCGACTTCATTCCAGCTGCGGCGGCGAT
AGGTGGTTCCCGCCGTGTCACGGCGGACATCTTATGGCCGAATTTGAGGA
AACCCAGTTCAAGGAATTCAAGGAAACCGTTTTTGCTTGACGATGATTTT
GAGGCTGGGTTCAGAGAGTTTAAGGATGATTCTGATTTCGATGAAGACGA
GGATGAAGATGATGATGAAGGGTTGTTGGTTGGTGTCAAAGGTTTCACCT
TTGGTTCCAATAACAACAAAGCTTCTAAGTCCTTTGTTCGTGGATCAAGT
GCTGCAAAATCTGTTGCAGTGAAATCAAACGAGCAAGCTGAAAAGGAATC
GAAGAGAAAGAGGAAGAATCAATATAGGGGTATCCGTCAACGTCCATGGG
GAAAATGGGCAGCTGAGATCCGTGACCCAAGGAAAGGTGTTCGTGTTTGG
CTCGGAACATTCAATACTGCTGAAGAAGCTGCAAGAGCTTATGATGATGC
AGCTAGGAGAATCCGTGGCAAGAAAGCCAAGGTGAATTTCCCAGAAGAGG
CTCCAATTGCTTCCTCAAAACGTCTTAAGACAAATCCTGAGACACAGCTG
TTGAATAAAAATCTGAACTCTTTCAAGCCGAATGGAAACCAAATGTTCAA
TTTCGGCGAAAATATGGAGAACTTCTATTCTCCTTTGGATCAGGTGGAAC
AGAAACCATTGGTTAACAACCAATATGCTGACATCGGAGCCTTCACCGGA
AATGGAGTTCAGCTTGCATCTGCTGATGTTACAGCTTATTTCAGCTCGGA
GCATTCAAGCAATTCATTTGATTATTCTGATTTATGCTGGGGAGAACAAG
GCCCGAAAACACCTGAGATTTCATCCGTGTTTTCTGCTGTTGCTGCTGCT
CCCGTGGAAGGTGAATCTCAGATGAACATGCAGTCTAACAACTCTCAGCC
TATGCAAGAGGAATCTGCAAAGACACTCTCTGAGGAGCTCGCAGATATCG
AATCCCAGCTGAAGTTCTTTGACAACTCCTTTGATGATAGCTGGAGTGAT
GCTTCATTGGCGGCTTTGCTCGGTGCTGATGCAACTCAGGATGGTGGAAA
CACAATGAACCTCTGGAGCTTTGATGACATGCCTTCCGTCGCAGGCGGAG
TTTTCTGA
>MaERF056
ATGGAACAAGCACTTCAACGTCTCAACGGAATGTCTTCAATGGAAGAACA
AGACAACCACCACCACCACCACCACAAAAAGACCCCCTCTTCCTCTTCCA
CCGCCGCAACAAACAAAAAGGGCGCCACGGTAAGATACCGTGGTGTCCGA
CGCCGTCCATGGGGCCGTTATGCTGCCGAGATAAGGGACCCACACTCAAA
GGAACGCCGCTGGCTTGGAACCTTTGATACAGCCGAACAAGCCGCTTGTG
CTTACGACTGCGCCGCCCGTGCCTTGCGCGGTTCTAAGGCCAGAACCAAT
TTCTTCTATCCCGAAACCAATGAACAACAAAAATCTCAACACCGCCATGT
GAGCAAGTTCAACGGTGGTGATTCTCTTACCCATACTTGTTCAAACCCTA
ACCCATGTTTGGTTCCTTCTTTTGTTCAAAACAACCCTAATAATACTATT
TCTTCTTCCTCCAATGTTAATGTTATACAACATGGTAGTTGTAGTGATTT
TATTTGTGGAAATGATAATAATGTTGTTGAAGAAATTATTGATGAAGACT
CGGATTTTTTTCCGAGAGAATCTACTGGTTTGTTGGAAGAGATAGTTCAT
AAGTTTATGAAAAGTACAAAAAAAGAAAAGAAGGTGAAAAATGAGAGTTA
TGGTTCAATTTCTCAACCAATATCTCATAACATTGTTCCTGTGGTTGAAG
GTACCTTTGGTACTGTCTCTTTTGATCACCTACAAGGTTTTCCAATGCAG
CAATTTGAGAGTTTTAATAATGGGTTTAACTTTAACAACACTGAGGTTTT
GTGTGTTGGGGAAAATCATCATGCAGCAGAGTGCTCTATCATGGAAGATG
TTTTTTATTATCCTCAACTTTTTAGTTCTTTTTGCAGCTAG
>MaERF057

ATGGCTGTTCCAAATTCTATTTCTAATGATATTGTTGGAACATTATTGTT
ACCAGATAAGGAACAAGAAGACTCTATCATAGTATCCACCCTCATTCACG
TTCTCTCCTCCTCTAATAATGTTGTTGCTCCTGTTTCTCAGACGCTGAAT
TGTCAGTGTACCCGTATCCAACAAAATTTCGGGGTGCCAACCAACTTACC
CCCTAACAACCAACATTACAAGGAAAGGGAATTAGTTAAAGCAGCCACGT
CATCAAGTGCAAGTGGGTCCCTCTTGTCATACCAGAACCAATATGATCCT
GACACGTGTTGTGTGTGCAAAATCAACGGCTGTCTCGGTTGCAGATTATT
CTTTGAGAAAGAGAAAGTGGAGAAGGGGAAGAAATACAGGGGAGTGAGGC
AGAGAGCATGGGGGAAATGGGTGGCTGAGATAAGAGACCCAAAAAGAGCA
ACTCGTGTTTGGCTTGGTACTTTTCAAACAGCTGAGAATGCTGCAAGAGC
TTATGATCAAGATGCTATTAAGTTTCATGGAGCTAGAGCTAAAATCAATT
TTGATTTCTCTGATTATGATGTAGTTGCTGATGAAAAGAAGCAAAGTGTG
ACAGTGCAGGATTTTGTTGGTGAAGTTTCAGGAGTGAAAAACCAAACAAA
AACTACTATCTTATCATTTGGGTCACCAGAAGTGAACTAG
>MaERF058
ATGTACGGAAATAGTAATTTTGATTCCGATCTTGCCCTTTTAGACTCTAT
TCGCCGCCACTTGTTAGGAGAATCAGAATTTATATTCGGAGCTCCAACAA
ATGTTTCGGGTAATACCCGGGTTTTCTCTCGGAGCTCCAGTTTCAGTAGT
TTATACCCATGTTTAAGTGACAATTGGGGTGAACTACCACTCAAGGAAGA
TGATTCTGAAGATATGGTACTTTACGGCGTCCTCCGTGACGCTGTAAACG
TCGGGTGGGTTCCGTCTCTTGAAGTCGGGTCACCTGAAAGTATTTCATCG
GTTTTTCCGTTAGAAATAACGGTTAAACCGGAACCGGATGTTATGCCGGT
GGAGAATATTCCCACGGTGGCGTCTACGGCGGAGCAAGTGGTTCCTGAGG
GACCAAAAGCTGCTCCGGTGAAAGGAAAGCACTACCGCGGCGTGAGACAA
CGGCCGTGGGGAAAATTCGCGGCGGAGATTCGTGACCCGGCGAAGAACGG
AGCTAGAGTTTGGCTCGGAACATTTGAAACCGCTGAGGATGCGGCTTTGG
CTTATGATAGAGCTGCGTATAGGATGAGAGGTTCAAGAGCTTTGTTGAAT
TTTCCTCTTCGGGTTAACTCCGGTGAACCCGACCCGGTTAGAATAGCTTC
AAAACGTTCTTCGCCGGAACGTTCTTCATCGGAAAGTGGTTCTCCGGCAA
AGAGAAAGAAAGTAATGACAGGTCAGAGTGGATTGAAAACAGGACAAGTG
GGAAGTCAAGTGGCACAACAATGTACACGTGGAGGACAGTTATTGGTTTC
CTAA
>MaERF059
ATGGCTAACTATGAAGAAGTTTCAGCTCTAAAACGCATTAAATTACATCT
TCTTGGTGAATTTTCTCCACTTCCATCTCCAATTTCAACACCATGTTTTG
ATTTTGATTTTGATTTTGAATTTCAAACAAACCAAACATCATCTCAATTC
GAATTTCAAACAAATCAAACCACTTCTCAATCTGAATCCAACTCTTCCAC
TTCTAATTCTTCAATTTCTTTGGATCAATATTTCACCAACCTCCTTGAAA
CAGATGCACAAATTCCTCTCTTTGAATTTGACACGAAACCACAATCAATG
GAACATGAGAGCCCCAAAGCTCTAACATCTCATCCTGTTAACCAACCTCA
ACGAACTGTAGAGAAAAAGCCTAAACTGAGCCGAAAACCGTCGCTGGAAA
TTGCTCTACCGAAGAAAACCGAGTGGATTCAATTCGGAAGCTCCGATCCG
AAGCCGGAGGTTGTTGCACAGAAGCCGGAAGTGGTTGAGAAACAGCACTA
CAGAGGAGTTAGACAAAGACCATGGGGAAAGTTTGCAGCTGAGATCCGTG

ACCCGAACAAACGCGGATCAAGAGTTTGGCTCGGAACTTTTGAAACGGCT
ATCGAAGCTGCTAAAGCCTACGATTCAGCCGCTTTCAGGCTGCGTGGATC
TAAAGCGATTCTCAACTTTCCGCTTGAAGTCAACGCGGCGGCGGTGGCGG
AGACCTCCGGCGACAAGAAACGTTGCCGTGAGGAGGAAGAGGAAGAGGTG
GTAGAAGTAAAACCGGTGGTGAAAAAGGAGAAAATAACGGAATATGATAT
GAACTGTTTTAAAGAGATGCCGTTAACGCCGTCATTTTGGACTGGCTTGT
GGGACGGTGATGTTAAGGGCACGTTCAGCGTTCCTTCGTCGCCGTTAACT
TCCTTTTGTTTGTCACCTTTAGTTGCTGTGTGA
>MaERF060
ATGGCTAACTATGATGAAGAAGTTTCAGCTCTTAAACGCATCAAATTTCA
TCTTCTTGGTGAATTCTCTCCACTTCCATCTCCAATTTCAACACCTTGCT
TTGATTTTGATTTTGAATTTCAATCAACCCAAACATCATCTCAATTCGAA
TTTCAAACAAATCAAACCACTTCTCAATCTGAATCAAACTCTTCCACTTC
TGATTCTTCAATTTCTTTAGATCATTATTTCACCAACCTCCTTGAAACAG
ATGCACAGATTCCTCTCTTTGAATTTGACACAAAACCACAATTAATGGAA
CATGAGAGCCCCAAAGCTCTTACATCTCATCCTTTTAACCAAAATCAACG
AAATGTAGAGAAGAAGCCTAAACTGACTCGAAAACCGTCGCTGGAAATCG
CTCTACCGAACAAAACCGAATGGATCCAATTCGGAAAACCAGATCCGAAA
CCGGAGGTGGTTGTACAAAAACAGGAAGTAAAGCAGCACTACAGAGGAGT
TAGACAAAGACCGTGGGGAAAGTTCGCCGCCGAGATCCGTGACCCGAACA
AACGCGGATCAAGAGTTTGGCTTGGAACTTTCGAAACGGCTATCGAAGCC
GCTAAAGCCTATGATTCAGCCGCTTTCAGGATGCGTGGATCTAAAGCGAT
TCTCAACTTTCCACTTGAAGTCAACGCGGCGGCGGTGGCGGTGGCGGTGG
CGGAGACCTCCGGCGACAAGAAACGTTGCCGTGAGGAGGAAGAGGAAGAG
GTGGTAGAAGTAAAACCGGTGGTGAAAAAGGAGAAAATAACGGAATATGA
TATGAACTGTTTTAAAGAGATGCCGTTAACGCCGTCATTTTGGACTGGCT
TGTGGGACGGTGATGTTAAGGGCACGTTCAGCGTTCCTTCGTCGCCGTTA
ACTTCCTTTTGTTTGTCACCTTTAGTTGCTGTGTGA
>MaERF061
ATGGACAAGGAAGACATTGTCCTTCTAGAACTTCAACCAACCACTTCTTT
GTCTTCCTTCATTATTGCCTCAACCTCTTCTAATTCCTCCTCCTCCTTAG
AAGAAGAAGAAGAAACTACTAACTCTAAATGCAAAAAAATAAGCAATACC
ATCAACAAAGAAAAGAAAAAGAAGAAAGTGGGAATGGTGAATAAAGTGAA
TGATGGGAAGCATAATCCAACGTATAGAGGAGTAAGAATGCGTCAATGGG
GAAAATGGGTATCAGAAATTAGAGAGCCAAGAAAAAAATCAAGAATTTGG
CTTGGAACTTTTCCAACTCCAGATATGGCTGCTAGAGCTCATGATGTGGC
GGCTCTTACTATCAAAGGCTCTTCAGCTTACCTCAATTTTCCCGAACTAG
CTGCCGTGTTACCACGTCCAGCTAGTGCCTCTCCCAAAGATATCCAAGTT
GCGGCTGCCAAAGCAGCCGCAACTGTTTACAACCATCCAATTACCAATCA
ACTCGAATTGGATGGTGAAGCTGAAGCTGAGTCGAGCCAAGCTGTTTCTT
CTTCTTCTTCTTCGTCTTCTTCAAGCCATAGTGATGGATCATCATTGAAG
GGTGAGGATGATATGTTTTTGAACCTTCCTGATCTCACACTTGATTTAAG
ACATAGTGGTGATGATGGGTTTTATTATTCTTCATCGGCTTGGCTTGTAA
ATGGAGCTCAACAAATAGAATCGGCTTTCCGGCTTGAAGACCCTATTCTA

TGGGACTCTTATCAAGTCACATAA
>MaERF062
ATGAAATCATTACTTGATGAGTCTAGTTACGTTCAAAAATCAGAGAACCA
CTCCTACACAAGCTCATCATCAGAAACATGTTGCTACGAAGAAATCTTGT
TAGCATCAGAACAACCAAAGAAACGTGCTGGGAGACGAAAATTCAAGGAG
ACAAGACATCCAGTGTACAGAGGAGTACGAAGGAGGAACAACAACAAGTG
GGTTTGTGAAGTGCGTGTTCCAAACGACAAGTCCACGAGGATTTGGCTAG
GAACATATCCAACACCTGAGATGGCAGCACATGCTCACGACGTTGCGGCG
TTAGCACTTCGTGGTAAATCAGCTTGTCTGAATTTTGCTGACTCGGCGTG
GCGATTGAATTTGCCTGCGTCGAACAATGCGAAGGAGATTAGGAAAATGG
CGGCGGAGGCTGCTTTGGGATTTGCCGTTGAGGATAGTAAGGAAGAAATT
ATGATTAGTAATTGTGAAGTAATTTGTAGCAGTGATGTTAATAGTGTTGG
TGTTATGGAAGTTGATAATAAACCTTTGCAGGGACTGTGTGTGGAAGTTA
CAGAGAAGGAGGAAATGTTGCATGATTGGTTTCGGAGTATGGCGAATGAG
CCTTTACGATCTCCGGCAACACCATTTATAAGATATGGTATTGGTAGGGA
TCATTGGAATAGTGTTGAAATTGACCAGGTTGATGCAGAGGTGTCCTTGT
GGAACTTTACCATCTAA
>MaERF063
ATGGCTTCTTCATCTTCTTCTTCTTCTTCTTCTTCCAAAAGACACCCAAC
ATACCACGGAATTAGAAGCCGAGGAGGAAAATGGGTGACTGAAATCCGCG
AGCCTCGTAAGACAAACCGCATATGGCTAGGCACATTCCCTACCCCTGAG
ATGGCAGCCGCGGCTTATGATGTCGCGGCTTTGGCCCTCAAAGGCGGGGA
CGCTGTTCTAAACTTCCCTAATTCAGCTAGCAAGTACCCGGTGCCTGCGT
CCAATTCACCGGATGATATCCGCAGTGCTGCCACTGCCGCAGCCGAATTA
ATGACGGCTGAAGCCGTTAATAATGATGCTGCTGGCTTTAATGGTAGTAA
TTGGTATGAAAATACTGAGTTTCTTGATGAGGAAGCTATATTCTCCATGC
CAAGATTAATGGTAGAAATGGCTGAGGGAATGCTGCTTTCTCCTCCTAGA
ATGAATCCACCACCGTCTGAGTATTTGCCTGAATACTATACTTCGGGAGA
AAGTTTGTGGAGCTATTATTGA
>MaERF064
ATGGTTCAATCAAAGAAGTTCAGAGGTGTCAGGCAGAGACATTGGGGTTC
TTGGGTTTCAGAGATTCGTCACCCACTTTTGAAAAGAAGGGTGTGGCTAG
GAACATTTGAAACAGCAGAAGAAGCAGCTAGAGCATATGATCAAGCAGCG
ATTTTAATGAGTGGTAGAAATGCCAAAACCAATTTTCCAATAACACAAAC
ACCTGAAGGTGATCCAAAGAGCAGCACAAGTACTGAGAACAAACCTTCAA
CATCTTCAAAGGATCTAGAAGAAATATTGCATGCTAAGCTTCGAAAATGT
GGTAAGGTACCATCTCCTTCAATGACTTGTTTGAGGCTTGACACAGAAAA
CTCTCACATTGGTGTGTGGCAAAAACGTGCTGGGAAGTGTTCTGATTCAA
ATTGGGTTATGACTGTTCAACTTGGGAAAAAGAAGAGTGTAACCGAAGAT
AGTGGTAGTAGTAGCAGTAGTAGTATTGTGCCTTCATCGGTGGTCGCAAC
AGGAGAGGAAATTGTGCGAGGAGAGATTGATGAAGAAGATAGAATTGCAC
TTCAAATGATAGAGGAACTTCTTAATGACAAGAACTGTCCTAGCCCAACA
ATTATCAATAATAAGCAAGGGGATGATATTCGTAACAGCTTTTTTCTTTA
A

>MaERF065
ATGTCAACCTCTAAAAAGATCACATCAGATTCATCTTTCAAAGGATATCA
TGAAACAAATCAAACACAAATGTGTCTGTCTCTACTTCAAAGGAACACCA
ACACATCAGTTTGTGGTGAAAAGAGAGGAAGAAGGAAACAAACAGAACCA
GGAAGGTTTCTTGGTGTTAGGAGAAGACCTTGGGGTAGATATGCTGCTGA
AATTAGAGATCCTACAACTAAAGAAAGACATTGGCTTGGTACTTTTGATA
CTGCTCAAGAAGCTGCTCTTGCTTATGACAGAGCTGCTATTTCCATGAAA
GGAAACCAAGCTAGAACAAATTTCATTTACTCTGATACCATCAATTTTCA
CACACTTGTTTCTTCTCCTATAGATCTTCAAACTCTCTTACCAGTTTCAC
AGTTACTCACTAGTAACACTCAAACAAATCAAAATAGTACTGTTTCTCAC
CTTAACACTTCACACACTAGTGGAAATGATCAAAACATTTCCTCATTGAA
CAATGACTTAATCATGAGTACTGTTGATCATGAAAAAACAACATCTTATG
GATCAACTCATGATAATAATTTCTTCTTTTCCAATGACACTAATAACTCT
GGCTATTTAGAATGCATTGTTCCTGATAACTGTTTCAGACCTGCTTCAAA
CTCAAGAAACAGCAATGTAAGTGCTTCAAGTGATGAAAAAGTTGATAACA
ATGCTGAAAGCAACAAAACTTCAATGGAAGGTCAATCACATTTTGGTATG
ACTTCATTCTCTCAAGAAATTCCAACAAGGGTCTCTAACTTTTCAGAGTT
TTCTTATTGTCCAAGTGAAATCAGTCAAGGTTTCTTGGATTGGAATTCTA
ATGAACTTTCAGCTATATTTAACAACAATCCATTAAGAGTTGAAGATGAA
TGCATGGATACATTAATGTATCCTAATTATCCCATCATTGAAAATCTAAG
TCAAAACTATGTGATGATGAATGATCAAGCTGCTTCTTCAACTAACTATT
CTCCATCACTTGAATTTGGTTACCCTCTCTTTTAA
>MaERF066
ATGGAGGAAGCACTTAACCGTCTCAACGGAACACCCTCAACCATCCAAGA
ATCTGAAACCAAAAAACCAAACAAACGCACTTCACGCCAAAACACCGCCT
CCGCTGCTGCTGGCAGCGGAGGCGGTACTAACCGCTACCGCGGAGTTCGC
CGCAGACCGTGGGGCCGTTATGCAGCTGAAATTAGAGACCCTCAGTCCAA
AGAACGCCGCTGGCTTGGTACTTTTGACACAGCTGAAGAAGCTGCTTGTG
CTTACGACTGTGCCGCACGTGCCATGCGTGGTCTCAAAGCTCGCACCAAC
TTTGTTTACCCTACTTCTCCTCCTCCAAACACCACTTTTCCTTCTTTCAA
CTTCACAAAAAATTCTCAAGCTTTTGAAAAAACTGCTACACATAACCGCC
ACGTGGCGTCTTGTGGTTGGTCAGAATCACAAGGTGTTGATTTTACTCAC
CAAAGAAACCCTTCAACTTCTTCACTTGACATGCTTCTGTTTCGTGAATT
CATTAACTCTTCGAATTCCAAACCTTCTTTGGTTTCTTCTTCTCCTCAGA
ATCACTTCCACGACCAGTATTCTAGTTCTAGTACTAGTTCTGTTTCTGGT
TCTGGTTCTACTTTTCACGCAGGTTGTTGTTTGGGGAATTCTTGCGGAGG
GGGTGCAAATAGCAACCATCACAACACTTTTGTTGGTTCAAAAGTTTCTG
CTGATGAAGACTTTGAGTTTTTTCCTAAAGAATCTTCTGATTCTGGATTA
TTGGAAGAGATTGTTAACAGGTTTTTACCGAAACCTAAGCAAGAGATTAA
GACGGAGATTTTGCCAAAGACAGCAACTTTCTGCGACCCTTTTGTTCCTG
CACCTGTGTATTATCAAAATGGTGTTGGGTTTGATCAAAATGGTCTAAAT
ATGCAGCAGTTTGAGAATTTTAATAATGGGTTTAATAGCACTTTTCATTC
TCTGCCTACTCATGGAAATGAACAGATTATGGCGAATCATGCTGAGAATT
CTGTTATTCAGTATCAAGATCTTCTTAATGCTTTTGCACTAAGGATGCAG

AATGCTTAA
>MaERF067
ATGAATTTCTTCATGTCATCATTTAGCGACTACTCAGACAATTCTTCTTC
TTCAGAAACAAGTTCATCAAGCCGTACAAGTACTTCTGAAGTGATTTTAG
CATCTGCTAGGCCAAAGAAAAGAGCAGGGAGAAGAGTGTTTAAGGAGACA
AGACATCCAGTGTACCGTGGAGTACGACGTAGGAACAATAACAAGTGGGT
TTGTGAGATGAGAGTTCCTAATAGTTATAACAAAAACAATAATATTAAGT
CAAGGATTTGGCTTGGAACATATCCAACACCTGAAATGGCTGCACGTGCA
CACGATGTTGCTGCACTTGCTCTTAAGGGAAAATCAGCTTGTCTTAATTT
TGCTGACTCAGCATGGCGATTGAGGTTGCCGGAGTCCAATGATGCGGTGG
AGATAAGGAGAGCAGCGATGGAGGCTGCTCAGCTGTTTGCGGCTGAGGAC
ATGTACAAGCAGGAATATACCGTTGAAGAACATATTCCGGCGGCCGCGGC
GGAATTTGAATTTGAAGACATGCAAGATTTACTTTTGAATATTGCGAATG
AGCCTTTACGTTCTGCTCCTCCTTCCCCAACAAATTATGGTAGCTATAAT
TGGGGTGATATAGAGATATTTGACACTCAAGTTTCACTGTGGAGCTTTTC
GATTTGA
>MaERF068
ATGACTACAAACAAAAACACACACCGTCTCAACCTTACAAAAGAAAACAC
TACTCCAAACAAACCATTTCCGAAAATTGTAAGAATCACCGTCACGGACA
AAGATGCTACCGACTGTTCCAGCGACGAAGATTCAACCACGCGCTTCTTA
AAACGTAACCGACCTAAAAAGTTTGTTAACGAGATTATCATTGAGCCATG
CGTGAGTGAGAATAACACCAGTACCGTTTCCAGGAAGAGAAACCGAAACA
GAAAAAGAACCACCGCCGGCGGAGGAAAAACAAGCGCTCCGGCGAAGAAA
TACCGGGGAGTGAGGCAGAGACCGTGGGGAAAATGGGCTGCGGAGATAAG
AGACCCGGCGCGTGGAGTGCGCGTGTGGCTTGGTACATTTCAAACTGCTG
AAGAAGCTGCTATTGTCTACGACAACGCGGCTATTAAGTTGCGTGGACCA
GACGCGCTAACTAATTTCATAACTCCACCTTCATCTGCCACGTGTCAGTT
ATCACCACCACCACCACTACCGGAAAATGAAAACGCTCCTCCACCTTCAT
TTCCGTTACCGGAAAATAGCAGTTACATTTCCGGTGAAGAATCTCAATCT
CAATCTCAAAACAATAATAATAAGAGTTTATTTTCACCTACTTCAGTTCT
TCAGTGTTGTTCATTCTCAGAAGAAGTAGCTGAATCTCAAAACAATGAGT
CAATGTTTTCAATTCCAAGTGATATACAATTTGATTTTCAGGGTTCTTCA
CCAGCAAATGATGCATTCTACAACTTCAATGGCTTTTCAGATAGCATGTT
CTACGGTGATATGGATTTTGAGTATTTTGATTTTGGTTTAGAAAGCTTGC
AGCCAATTAAGGATGAAGATTTTTTTCAAGATATTGATGATTTATTTGCT
TCAGATGCTCTTCTTGCTGTTTAA
>MaERF069
ATGGAAGGAATGCAAAACAGAGATATTGGATCATGTTTATCAAATCTCAT
ATTATCAAGTTCAACAAACACACTTGACTCAATTTTCTCAGATATTCCAT
CAACAAACACCACCACAAACAACAACACCTTCTTGGGTTGTTCAAATTTT
GAACCTTTAGGTTCCTCTGTTTACCTTCGCCAGAGAGATATTTTACAAAA
ATTCTACGAAGAAAGCCGATTAAACGGCTCATTCGTTCCATCTTCATTTT
CAAATCAATCTCTACTAAACCCATCTTTATATACAACAAATTCAAGTTCA
GTTTCAAGTACAAGTTCGTTCACTTCTTTGGTGAATCCTTGCAAGAAAAA

GCTATACAGAGGAGTGAGACAGAGACACTGGGGAAAATGGGTAGCTGAGA
TTAGACTTCCTCAGAATAGAATGAGAGTTTGGCTTGGAACTTATGAAACT
GCTGAAGCTGCAGCTTATGCTTATGATCGTGCAGCTTATAAGCTTCGCGG
CGAATACGCTCGTTTGAATTTTCCAAATTTGAAAGATCCAACAAAGTTAG
GTTTTGGTGATTCTACTAGATTGAATGCTTTGAAGAATTCTGTTGATGCT
AAAATTCAAGCTATTTGTCAAAAGGTGAAAAGGGAAAAAGCTAAAAAGAT
TGCTGCTAAAAAGATGAAGAAGAATTCAGGTAGTGAAGCTGCAAAGAGTG
ATAAAAATTCAGAGAAGATTATTAATTCATCTTCTTGTTCTTCTTCTTCG
TCTTCGTTACCGATTTCACCTATAACTTTTTGTGATGATTGGGTGAATGA
ATTGTTTTCACCAACTGTTTCTGAGGATGGAATTTGGAAAGGAGAGAATT
CGCCGAATTCTGTTTCGACAATGGTGACAGAGGAAACAGAGTTTGAAGAT
TGTTCCTTAGCAAGGATGCCATCTTTTGATCCTGAATTGATTTGGGAAGT
TCTTGCTATTTAG
>MaERF070
ATGAATTGGTCTACTTCAACAACAAGCACAAGTGATGAAAACAACAAGAA
ATTTAAGGGAATTAGGCGTAGAAAGTGGGGAAAATGGGTATCAGAGATTC
GTGTTCCAGGGACTCAAGAACGTTTATGGTTGGGAACTTATGCTACACCG
GAAGCTGCTGCGGTGGCACACGACATAGCTGTTTATTGTTTAAAGAGACC
TTCTACTTTGGACAAACTCAACTTTCCTGAGATTTTGTCTTCTTATGGTA
TTCAACAAAGAGATAATTTGATGTCTCCGAGGTCTGTTCAGAAGGTTGCT
TCTGATGTTGCAATGGATGTTGATGCACGAAAGATTGCAAGCCAAGCAAT
AACTTTGCCAGAAACTCATCACATGAATAATGTTGTTGCTGATGATGTGT
TTTGGTGGGAAGGTTTGGGTGATGAACAAGGAGTTACTCATGTTTCTAGC
CAACAACAAGAGAGCTTGAACATTTCCATTGAAGATTATCTTTAG
>MaERF071
ATGTCTTCAAGTGGTGCTGCTAGTTCTGCATACAGAGGAGTCCGCAAGAG
GAAATGGGGAAAGTGGGTTTCCGAAATCCGCGAGCCGGGAACCAAAACAA
GAATATGGTTGGGAAGCTTCGAGACACCGGAGATGGCAGCAGCTGCATAC
GACGTTGCTGCCTTGCATTTTCGGGGACGGGAAGCCAGGCTCAATTTCCC
TGAACTCGCAACCACTCTTCCTCATCCTTTAAGCAACAATGCTGACCACA
TTCGAATGGCAGCACACGAGGCCGCCTTGAGACTCAGAGCCAACATGCTG
GTGCCACCAGATAATAACAGTGGAACAGGCTCAGCCAGTTCCACTGATGT
GGTGGCGCCTCTGACTGTGAGACTCTCTCCGAGTCAAATTCAAGCGATTA
ATGATTCGCCTATGGATTCACCTCCTACATGGATGCAAATGTCACACCCC
TTTATGATGGATGATCAAACCGTGTTGTTTGGTAATAATGGATACGGCTA
TGAATTTGATGAGAACGAATGGGAGGATATGCAACACACTGATTATCTTT
GGGATCCTTGA
>MaERF072
ATGGAAGGTGGTGAAAGAGGAAAAGGAGAGAGAAAGAGAAACGGTGGTGG
TGGTGGTGAAGGAGAGAGAAGATACAAAGGAATTCGTATGAGAAAGTGGG
GAAAATGGGTAGCTGAAATTCGAGAACCAAACAAACGTTCACGAATTTGG
CTAGGTTCATATTCTACACCTATCGCCGCCGCTAGAGCTTACGACACCGC
TGTTTTCTATCTCCGGGGACCATCAGCTCGTCTCAATTTCCCGGAGCTAC
TCGCCGGTGAAAACAACGCGGTTGTTGGTGGCGGAGATATGTCGGCTGCT

ACTATAAGAAAGAAAGCTACTGAAGTTGGTGCTAGGGTTGATGCTCTTCA
AGCAACTGTTCATCACCATCACCATCATCAGCATAACCGGAACCAGCATC
GTGTTATGCCGGTTCCGGAGATGCTTAACGGTGATGGTGATTTTGCTGAG
AGGGTTGATTTGAATAAGGTTCCTGAACCGGAGAGTTCGGGTTCTGAGTG
GGATGTTAATTGA
>MaERF073
ATGTACACAGAGAGTAATTATGAATCCGACTTGGCTATTTTAGAATCTAT
TCAGCGTCACTTGCTCGGAGATTCCGATGTTTTTACATTCGGAGCTCCAT
CTAATGTTCCAGTTTATTGTCGGAGCTCAAGCTTCAACAACCTTAAACCA
TGTCTGAGTGAGAATTGGGGTGAACTTCCACTACAAGAAAACGATTCAGA
GGACATGATTCTTTATGGTGTACTTCGTGATGCTGTTAACGTTGGGTGGG
TTCCATCTCTCGAAGCATCTTCACCAGAGAGTTTTTCATCTGGTTTCACG
CCGGAAAAGATTGTTAAATCAGAGCCGGATATTTTCCCGGAAATGCAGAG
TTCGTCTGAGATGATTGTTTCTACGCCGGTGGTTGCGCCGGCAACGGGGA
AGCATTACCGGGGAGTGCGACGACGGCCATGGGGAAAATATGCCGCGGAG
ATTCGTGACCCGGCTAAAAATGGCGCGCGGGTTTGGCTTGGAACATTTGA
AACAGATGAGGATGCAGCTTTGGCTTACGACCGAGCCGCATATAGGATGC
GTGGCTCGCGGGCTATGTTGAATTTTCCGCTCCGGGTTAATTCGGACGAA
CCCGACCCCGTTAGAGTGTCGTCAAAGTCAAAACGATACTCATTGGCTGA
GTCATCTTCTTCATCGGAGGGTGGTTCGATAGTGAAACGGAGGAAGAAGG
TGGTGGGGGAAATGGTGACTCCTCAAGGTGGAATGAAAATGGCGCAAGTG
GATTTTGGGTCACGACAGAAGTTTAGAGGATCATCCACATTGAGTTAA
>MaERF074
ATGGAACAAGTTTCTGTCCCAACAGACAAAGAAATTGAACTAACCACTTC
CTCCTCCATTGAAGATAACAAAACCAAAAACAAAGGTGATAATGGGAAGC
ACCCATTGTATCGTGGAGTACGAATGCGTAGTTGGGGAAAATGGGTTTCA
GAAATTAGAGAGCCAAAGAAAAAATCCAGAATCTGGCTAGGCACTTTTCC
TACACCAGAGATGGCAGCTCGGGCCCATGACGTTGCGGCCCAAGCCATCA
AAGGCAGCTCAGCTTACCTCAATTTCCCCGAATTAGCTCAATATCTTCCG
CGCCCAGCTACCAATTCTCCTAAGGACATTCAAGCCGCTGCTGCTAAGGC
AGCGGCTATGGACTATCACCATGAAGCCCAGAACCAGGCTGAGGCCCGCG
CCATACAAGAATCCTTAAATGATGAGGATGACACATTCTACGACCTCCCT
GATCTCTTACTCGATTCAAATAAGAGTTCAAGTGAGTTTCATTATTCTTC
CATACCATGGTTCGTGGCTGGAGCTGAATATCCAAACCCAAGTTTTGTGC
TGGATGAGCCTTTAACGTGGGAACCTTAG
>MaERF075
ATGTATCCAACTAACAACAACTCTGTCTCTTCCTCTTCCTCCGACATGTC
GCTTCCGAATTCAGAGGGCTCTCATTGGATGTCTGTTTGCAAAGAGGAGA
TGAGGTTAGCGGCAACTACTCCGAAGAAGCGTGCAGGGAGGAAGAAGTTC
AAGGAGACTCGCCACCCGGTGTATAGGGGCGTGAGGAAGAGGAACTTAGA
TAAATGGGTTTGTGAAATGAGGGAGCCTAACAAGAAGACTAAGATTTGGC
TAGGCACTTTTCCAACTGCTGAGATGGCAGCCCGGGCACACGATGTTGCT
GCAATGGCATTGAGGGGCCGCTACGCTTGTCTCAACTTTGCAGACTCTGC
GTGGAGGCTCCCTAAACCTGCCAGCACTCAGGCAAAAGATATACAAAAGG

CGGCTACAGAGGCCGCTGAGGCTTTCAGACCAGACCAGACTTCAATGACT
AATGACAATGACAATGACAATGACCGTGACCGTGACCGTGAGATGGCTGT
AGTTGCCACTGCAACAACAGAGAAGCAGAGTATGAATTTTATGGAAGAGG
AAGAAGAGGGAGTGATGAACATGCCAGAGATGTGGAGGAATATGGCGCTA
ATGTCCCCTACACATAGCTTTGAGTACCATGATCAGTATAAATATATTAA
TGAAGAATTTGAAGATGCAGAGGTATCACTATGGAGTTTTTAA
>MaERF076
ATGTTTACAACTAACAACTCTTCCTATTCACACCCCTTTTCTCCAAATTC
GTCTGAAAATTCATTACAGAATTCAGAAGGCTCTCAAGGGATGTCAATTT
CCAACGAGGAGGTGAGGTTAGCTGCGACTACTCCGAAGAAGCGTGCAGGG
AGGAAGACGTTTAAGGAGACTCGCCACCCGGTATACAGGGGTGTGAGGAA
GAGGAATTTAGATAAATGGGTTTGTGAAATGAGGGAGCCTAACAAGAAGA
CTAAGATTTGGCTAGGCACTTTTCCAACTGCTGAGATGGCAGCCCGGGCA
CACGATGTTGCTGCAATGGCATTGAGGGGCCGCTACGCCTGTCTCAACTT
TGCAGACTCGGCGTGGCAGCTCCCTAAACCAGCCACCACTCAAGCAAAAG
ATATACAAAAGGCGGCTGCACAGGCCGCTGAGGCTTTCAGACCAGACAAG
ACTTCGAAGAATAATGACTTTGACATGGCTATATCCGCCTCTGCCACAGA
GGAGCAGAATAGTCTTTGTATGGAAGAGGAAGAAGAGGGAGTGATGAACA
TGCCAGAGATGTGGAGGAATATGGCGCTTATGTCACCGACACATAGCTTT
GGGTATCAAGAGTATGAAGATATTCATGCTGAAGAGTTTCAAGATGAAGA
GCGAGGATGGATTAAAGCAGATCTGAAGCAGAAGAAGGATGGATGTGCAT
ATCTAAAGCAAAAAGGGGATGTGGATGGAAGAAGAAAGATCACGGGTGAT
GGTGGTGACAGAGGAATTAGCTAA
>MaERF077
ATGTTTACAACTAACAGCTCTTCCTATTCACACCCCTTTTCTCCAAATTC
CTCTGAAAATTCATTGCCGAATTCAGAGGGCTCTCAAGGGATGTCAATTT
CCAACGAGGAGGTGAGGTTAGCCGCGACTACTCCAAAGAAGCGTGCAGGG
AGGAAGAAGTTCAAGGAGACTCGCCACCCGGTATACAGGGGTGTGAGGAA
GAGGAACTTAGATAAATGGGTTTGTGAAATGAGGGAGCCTAACAAGAAGA
CTAAAATTTGGTTAGGAACTTTTCCAACGGCCGAGATGGCAGCCCGAGCA
CACGATGTTGCTGCAATGGCATTGAGGGGCCGCTACGCCTGTCTCAATTT
TGCAGACTCTGCGTGGCGGCTCCCTAAACCCGCCAGCACTCAGGCAAAAG
ATATACAAAAGGCGGCTACACAGGCCGCTGAGGCTTTCAGACCAGACCAG
ACTTCAATGACTAATGACATTGACACGGCTATAGCCGCCTCTGCCACAGA
GGAGCAGAGAAGTCTTTGTATGGAAGAGGAAGAAGAGGGAGTGATGAACA
TGCCAGAGTTGTTGAGGAATATGGCGCTTATGTCACCTACACATAGCTTT
GGGTATCATGAGCATGAAGATATTCATGTTCAAGACTTTCAAGGTTTTCA
AGATGAAGAGAATGGAAACAGTTCTGTTCTACAACCATTTCTATTAGTAG
TACTGATTCTTCATATATACATAGCCGCGTTTGTCCGTAATATCATGGAT
TGTGATTGTTGCCGTCGTGACCTTACAATTGGCAAAGTGCTAAAATTAAT
GGGGTTTGCTACTTATAATCAGAAGCCAGTGATTAATATGCTCAGCAATT
TCCTAAGGAGTTTCATTCAGAAGGGTATGTTTCTAGCTTCTTTGGAATTG
GACTTTTACTTCTTTTTCCCATCTTGA
>MaERF078

ATGATTACTACTAACAACTCTTCCTATTCACACTCCATTTTCTCAAAAGC
ATCTTCTTCCTACGACACATCATCACCGGATTCGGAGGTACGATTAGCGG
CTAGTAACCCGAAGAAGCGAGCAGGGAGGAAGATATTCAAGGAAACTCGC
CACCCGGTGTATAGGGGTGTGAGGAAAAGGAACTTAGATAAATGGGTTTG
TGAAATGAGGGAGCCCAACAAAAAGACTAGGATTTGGCTAGGGACTTATC
CAACAGCCGAGATGGCAGCCCGAGCCCATGATGTGGCTGCCATGGCATTG
AGAGGCCGCTACGCCTGTCTCAATTTCGCAGACTCGGTGTGGCGGCTCCC
TATTCCAGCAACCTCCAATATAAAAGATATTCAAAAGGCGGCTGCTGAAG
CCGCCGAAGCTTTTAGACCAGACAAGACTTTAATGACTAACGATATTGAC
ACGGTCGTAGCTGTCATCGCCACAAAGGAGCTGAATATTTTTTGTGTGGA
AGTTGAAGAAGAGGATGAAGTGTTGAACATGCCAGAGTTGTGGAGGAATA
TGGCGCTAATGTCCCCTACACATAGCTTTGGGTATGATGATCAATATGAA
GATATTCATGTACAAGACTTTCAAGATGATGACGAGGACTTTAAAAAAAG
GACTATTACAATGAGTTGGGTTGTGACTGCAATTGGAGTTTGTTCGCCGC
GTTTCACCGTAATATCAAGGATTGTGATCGTCGGGTTGACTCCGAACCCT
GCTTTTCTATGTCTGGTTTCTTTGTTTTCTTCTTGA
>MaERF079
ATGGAAATTCAATTTGAGGAAGCAAAGAGTTTGAGACCAAAAAGGGTGAA
CAAATTCAAAGGGAGAAACAAAAAGAGTGAAAAAAGAGACAAGTTTGTTG
GGGTGAGACAAAGACCATCAGGGAGATATGTTGCTGAGATAAAGGACACA
ACACAAAACATAAGAATGTGGCTTGGAACATATGAAACAGCCGAGGAAGC
GGCAAGAGCTTACGACGAAGCCGCAACACTTCTTCGCGGTTCCAACACTC
GCACCAACTTCGTCACACATGTTTCCTATGATTCCCCTCTCGCTTCTCGG
ATTAAACATCTTCTCAACAACCGAAAAAAAGGTACCAAACAAGAAGAAGA
TATGGATGTTAGAAGCAGCACCACTAGCCGTGCTGACACGTCTAGTGGCA
CTACACATCGTACTAATGCTAGTAGCACCACTAGCCGTGCTGACACGCCT
AGTGGCACTACTAGTACTAATGCTAGCGGTACTACCAATAGTACTAGTAC
TAGCAACGTCACTAGCACCAATGCGGTTATTAGTATTAATTCGAGCAATA
ACAACATCGATGAAAACATTGAAAACCCGGTTTCTAGTGTGACGGCAATA
CAAAACACAAAGCTATTTGATGATGCATATAGGCCAGATTTGAGCAAAAT
CAAAGAGTATGAGACAGGTTCAAAGTCCAATGTTAAATGGGATTTTGGAC
CTATTTTTGATCATTTTCATTTTGGTCAAGGGTTGGATATGACAAACAGT
GATGGAATATTGTATGATATGGTTGATGAAGGAGTTTCAGAATTTGAAAG
GATGAAAGTTGAAAGACAAATATCAGCTTCACTTTATGCAATTAATGGTG
TGCAAGAATACATGGAAACTGTTCATGACTCCAATGAAGCTTTATGGAAT
CTTTCACCTTTTTGCTCATTTTTATGTTGA
>MaERF080
ATGTTAGTGAAAAGCCAAACTAACGGGGATGGACCCAAGTCCCTCGCAGA
TACATTGGCAAGATGGAAAGAATATAATGCACGGCTTGAATCGAGTGGTG
AAGCTGTGAAGCCAGTTAGGAAAGTTCCTGCCAAAGGATCAAAGAAAGGG
TGTATGAAAGGTAAAGGGGGACCCGAGAACTCGCGTTGTAATTATAGAGG
TGTTAGGCAAAGGACGTGGGGAAAATGGGTTGCTGAAATTCGTGAGCCAA
ACAGAGGAAATAGGCTCTGGTTAGGTACATTTTCAAATGCAGTAGGCGCT
GCTCTTGCTTATGATGAAGCAGCGAGGGCAATGTACGGTTCTTGTGCGCG

ACTGAACTTTCCCAATGTAGAAGTGACCAATTTCTCTCATATGGAATCTT
TGAAAGATTCTCCTGTTGCAGAACAATCTGGTTTTGTGAAGACAACAGCA
GAAAATACCGAGTCTGTGATATCGCCCGATAACAATCGGGTAGATGCAGA
TGAGGATGTTGACATGAAACATCTTTCCTTGTCCTTAAGTATGACATCGA
ATCATGAGGAGAAAGAGGGTGAATCAGGGACTAGTTGA
>MaERF081
ATGAATTTTTATTCTCCTAATTTAAAAAACCAAAACCAATACTTCACTGA
AAACTCCTTCTCGAATAATATCTATTGGGAATTAGAAGATTTCGTTAATG
TCTTCAATGATGATATCACTTCCAACAAGTACTCACTATCACAAACGAAA
GAATCTTTATTATTACCATTATCATTATCTGCTGAGTCCAACTCTTCATT
ATCATTGCTAGGCAATGGATCACTAGAAGTCTCATCCAATACACAAGTGA
TCAAAGATACACAATCATCATTATCATCATTATCATCCTCGCCAATTAAA
GAAAATAACCAAAGCGTGTTTAGAGGAGTAAGAAGAAGGCCATGGGGGAA
ATTTGCAGCGGAGATAAGGGACTCAACAAGAAAAGGGGCTAGAGTATGGC
TTGGAACATTCAACACGGCCGAAGAAGCTGCTTTAGCTTATGACCAAGCC
GCTTTTTCAACAAGAGGCTCATCGGCAGTGTTGAATTTTCCAGAGGAAGT
AGTTAAAGAATCACTGAAAGAAATGGCTAAAAACTCTAAGCCTTTGGAAG
AAGGTACTTCCCCTGTGTTGGAAATCAAGAGAAAACACGTGAAGAGAAAA
TCATCCAAGGAGGTTCGTAAAAAGAAAACCGAAGCTGATCATCATAGTGA
TCGAATTCAGATAGAGACTAATACTAATTCTCAAAATGTGTTTGTGTTTG
AGGATTTGGGTGCTGAATATCTGGAGCAACTATTGAGCTTGAATTCATGA
>MaERF082
ATGAATTTTGATTTTCCTGCCTTAAAAAACCAAAATCTTCTATACTTCAC
TGAAAATTCCTTCTCTAATAATATCTCTTGGGAACTAGAAGATTTCGTTA
ATTTCTTCAATGATGATATCAATTCCAACAAGGACTCACAGTCACTAACG
AAGGCCTTCAATGATGAATCATTATTATTACCATTATCATTATCTGCTGA
ATCCAACTCTTCATTACCATTGCTAAGCAATGGATCACTAGAAGTCTCAT
CTAATACTACTTATACACAAGTGATCAAAGAGACACAAACAGTATCATTA
TCATCATCCTCCTTGCAACCACCGCCAATTAAAGAAAGTAACAAAAGGGT
GTTTAGAGGAGTAAGAAGAAGGCCATGGGGAACATTTGCGGCGGAGATAA
GGGACTCAACAAGAAAAGGGGCTAGAGTATGGCTTGGAACGTTCAACACG
GCCGAAGAAGCTGCTTTAGCTTATGACCAAGCCGCTTTTTCAACAAGAGG
TTCATCGGCAGTGTTGAATTTTCCAGAGGAAGTAGTTAAAGAATCACTCA
AAGAAATGGCTAAAAACTCTAAGCCTTTGGAAGAAGGTACTTCCCCTGTG
TTGGAAATCAAGAGAAAACACGTCAAGAGAAAATCATCTAAGGAGGTTAG
TAAAAAGAAAATAAAAGCTGATCATCATAGTGATCGAATTCAGATAGAGA
CCAATACTAATTCGCAAAATGTGTTGGTGCTTGAGGACTTGGGTGCTGAA
TATCTTGAGCAACTATTGAGTTTGACTTAG
>MaERF083
ATGGTTACCAAAACAAAGAACAACGCCAATCTCAAAATCAAAAAGCCTTT
CAACAACAACAACAACAATGCAACCGAGATTCACTTCCGCGGTGTAAGAA
AGCGACCATGGGGCCGTTACGCCGCCGAGATTCGCGATCCCGGCAAGAAA
AGTCGCGTTTGGCTCGGCACTTTCGACACCGCCGAAGAAGCCGCCAGAGC
CTACGACAACGCCGCTCGTCAGTTCCGCGGCCCCAAAGCCAAGACCAATT

TCCCTCCTCCTCCTCCTCCTTCCGACACCAAGGAAGATAGCCCCAGTCAG
AGCAGCACCGTTGAATCATCCGCCCCCAACCCTGAGCGTGAAGTCACGCG
CCCTCGTGAAGTTTCCGCCGGAGGTGGCGTCATGGATCGGTTTCCGTTTC
TCTCGATTCAGCAACAGATCATGCCGTTTTCTGCCGGCGCTGGTGTCGGC
GCCGGTGCTGTTGAGGGAATGGTGACTGCTATGCATCCGGTTTTCTTTTA
CGATCCAGCGGGTCGAGCTGAGTTTCTGAACAAGCGGTTTACTAACCGGT
TTGAACCGGAACCGGTTCAGTTTAACATCGGGTTTGGCGGTGGTGGTGGT
GGGGTCCAGAGTGATTCAGATTCATCATCTGTTGTTGATTGTCAGCCGAA
GAGAGCTCTCAATCTTGATCTTAACTTAGCTCCACCATCGGAATATTAA
>MaERF084
ATGAACTACTCTTCCTCCTTATCTCCCACCACCCTCACCTCCGATCAAGA
ACTCTCCGTCATCGTCTCTGCCTTAACCAACGTCGTCTCCGGCTCCACCT
CCACCGAATTTCGCCTCCCGGATCCAACCGTCGGAAGCAGCAGTAGCAGC
TTAGAAAGAATAGTTCCACCGACGAACGTGGAAACCTGCCGGGAATGCAA
CATAGCAGGATGTTTAGGATGCAATTTCTTCTCTGAAGAGAACAAGAAAA
AACAAAAGAGAGCTAAGAAGAAGTACAGAGGAGTGAGACAGAGACCATGG
GGAAAATGGGCTGCTGAGATTCGTGACCCGAGACGAGCGGTCCGTGTTTG
GCTTGGAACTTTTACCACGGCGGAGGAAGCTGCCAGAGCTTACGACAATG
CCGCCATCGAGTTCCGTGGGCCAAGAGCCAAGCTCAATTTTCCACTTGTG
GATGAATCACTTAAGCAAGTTGAGGAGGAGCCAGAAGTGGTTGTTCCTTC
GGAGCATGTAAAGGAGGAGAATATGGATCAAGAAATGCAAATTGAAACAA
CGGTGGGATTTGAGAATAACAAGGATAGTGATTTTTGGGATAGTATTGGG
GAAGCTGATTTTCAACAACTTATGAGGTTTATGGATTTTGGTGGAGATTC
TTCTGGTTCTAGAACAGGGAACACTTTTCATTAG
>MaERF085
ATGAACTACTACTCCTCTTTCTCCCCCGCCACCCTCACCTCCGATCAAGA
ACTCTCCGTCATCGTCGCCGCTCTAACCAACGTAATCTCCGGTTCAACCT
CCACCGGAAGCAGCAGCAGCAGCTTAGAAAAAATAGTTCCACCGGCTACC
ATGGAAACCTGCCGTGAATGCAACATAGCAGGATGTTTAGGATGCAATTT
CTTTCCCGAAGAGAAAAAACAAAAACAAAAACCAAAGCAAAAGAGAGCGA
AGAAGAAGTACAGAGGAGTGAGGCAGAGACCATGGGGAAAATGGGCAGCA
GAGATTCGTGATCCGAGACGTGCTGTTCGTGTTTGGCTCGGAACATTTAC
AACAGCGGAGGAAGCTGCCAGAGCTTACGACAATGCCGCTATCGAGTTCC
GTGGACCAAGGGCCAAGCTCAATTTTCCTTTTGTTGATGAGTCACTTAAG
GAACAAGAAGTGGTTGTTCCTAATTTGGAGAATGTAAAGGATGAGAGTTT
GAATCAAGAAATGCAAACAGAGACAAATATGGGATTTGAGAATAATATGG
ACTATGATTTTTGGTATAGGATTGGGGAGGATGATTTTCAACAGCTTATG
AGGTTTATGGATTTTGATGGAGATTCTAGAACAGGGAGCACTTTTAATTA
G
>MaERF086
ATGAACTACAACTACTCTTCCTCCTTCTCCCCCACCATACTCACCCCCGA
TCAAGAACTCTCCGTCATCGTCTCCGCCTTAACCAACGTTGTCTCAGGCT
CCACCTCCACCGAATTTCGGCTACCGGATCCAACCATCGGAAGCAGCAGC
AGCAGCAGCTTAGAACGAATAGTTGCACCGGCAAACGTGGAAACCTGCGG

GGAATGCAACATAGCAGGATGTTTAGGATGCAATTTCTTCTCTGAAGAGA
ACAAGAAAAAACAAAAGAGAGCTAAGAAGAAGTACAGGGGAGTGAGGCAG
AGGCCATGGGGAAAATGGGCAGCAGAGATTCGTGACCCGAGACGTGCTGC
CCGTGTTTGGCTCGGAACATTCAACACGGCGGAGGAAGCTGCCAGAGCTT
ATGACAATGCTGCTATCGAGTTTCGTGGACCGAGGGCCAAGCTCAATTTT
CCAATGGTGGATGACTCTCTTAAGAATGTGGAGGAGCCAGAAGTGGTTGT
TCCTTCGGAGAATATAAATGATGAGAATATGAATGACCAGGGAATGCAAA
CAGAGACTAATATGGACTGTGATTTTTGGGATAGTATTGGGGAAGCTGAT
TTTATGGATTTTGGTGGAGAGTCTTCTGATTCTAGAACAGGGAACACTTT
TAATTAG
>MaERF087
ATGGAACATCAAAAACAAAAGAGCAACGAAGACAAGCGCAGAGAGAAACT
AATGATAAAGAGTAGGTCCAAGTTTGTTGGAGTTAGACAAAGGGCTTCAG
GGAAATGGGCAGCAGAGATTAAAGACACATCAAAGAACATTAGGATGTGG
CTTGGTACCTATAAAACAGCTGAGGAAGCTGCTCGAGCTTATGATGAAGC
TGCTTGTCTCCTTAGAGGTTCAAACACTCGTACCAATTTCTCTACTACTC
ATTCCATTCCTACCAATTCTCCTATATCTCTCAAACTCAAAAACATCCTT
CATCGTAAATCCATCTCAAATCAAAGCCAAATTCAAAGTCAAAACAAGTC
TACTATGATGAGTTCCTCTTTTCAAGGTGCTCCTATTGATAATAACATTA
TGGTAATGGAAAACAACTCTTCTTCATCTAGTAGTGAAGAGTCAAATTCT
TTGTTTTGGGCTCAGAATCAAGTATCTGAATATAATCCTTATGGTGTTGA
TATGAATATGATAAACTGTTCAATGAGTATTACACCAAATACATTGGAGT
TTGATTATCCTTGGTCATTGTCCCAAGAAAGGATTAACGAGTTAACAACA
CCAAAAGATAATATGAATGTATATGGAATGAATGAGTGCTATGTGGATGA
TACATATGAATCTAAGTATGAATGTGATGCCAATTATCCATTTTCTCACT
TGTTCTGTTTTGCTTGA
>MaERF088
ATGGCAACAACCATAGATATGTACAATAGCAATAAAAACATCACACATGG
TTTCTTAGATCCATATAACGAAGAACTCATGAAAGCACTTGAGCCTTTTT
TGAAAACTAGTTCTTCATCAATCACTGAACAATCTCCACTTACACCAAAT
TTGAATCACAATTACTTCTCCTACGGAACTCAAACAAGCTCCATTGGACT
CAACAAACTCACTGCATCACAAATACTTCAAATCCAAGCTCAAATCCAAT
TCCAAACACCTCAACAACACCACCAAAAAAGCCACCTTACTCAAAAGCCC
GTTCCAATGAAGCACTTTGGAACTCCCTCAAAATCCACGAAACTCTACCG
TGGAGTTAGACAACGTCATTGGGGAAAATGGGTTGCTGAGATAAGACTCC
CAAAAAACCGTACTCGTCTCTGGTTAGGTACATTTGATACAGCAGAGGAA
GCAGCTTTTGCTTATGATAAGGCAGCTTATAAGCTTAGAGGTGAATTTGC
ACGTCTCAATTTTCCTCATATGCGTTTTCATGGAAACTACAATCCTCTCC
CTTCCTCGGTTGATTCCAAGCTTCAAGCTATTTGTGAAACCTTAGCCATT
TCTCAAAAACAGGGGAATACAGAAAAACTCTGTTCCTCTGTTGAAGATGT
AAAACCTGTTGTTTGTGTTCCTGCACAATTGAAAGATGAATTGGTTTGTC
ACGAGTTTGAGGAGTTTAAGGTTGAGAGCACTGTGTTGTCGCGTATTTCT
GATGAATTTTTACCTGGTTTGTCATCTCCGGAATCAGGTGTTACTTTCTT
GGATTTCTCAGATTCTAGCCAGTGGGATGAAGTGGAGAATTTTGGGTTGA

AGAAGTACCCTTCTGTTGAAATTGATTGGGAAGCATTATGA
>MaERF089
ATGCCAATGATGTTTCCTGGTATTAATAGAGAGGGAGAGATGTCAGCTAT
GGTCTCTGCTTTAACACATGTGATATGTGGTGATCAGAATAATGATGATG
GTAGTGTTCTGAATCAGAATATGAATATGAATCAGAGTACATTTGAAGGA
GATTGTGTTGATATCAAGCCTTTTGGTTCTTCTTCATCTTATGGTGGTAA
TTCTCTTCTCAAGAGAAGAAGGGAAGATGGTGGATTCTTTGACAATTCAT
CCTCTGCTATTCCCCAAGGTGTAGAATGTCCAAGCAACTGGACAAACACA
ATAACAACAACAGGAAGGAGCCAAATGGAAGAACAAATATACGAATACAG
AACAGACAATAACATTAATGTGAAAAATGAAGAGCAACCAAAAAGAAAAT
ACAGAGGAGTAAGACAAAGACCATGGGGAAAATGGGCTGCAGAGATAAGA
GATCCTTTCAAAGCTACAAGAGTATGGTTAGGTACATTTGAAAACGCTGA
AGATGCAGCCAAAGCCTATGATCAAGCTTCATTGCGTTTTAGAGGAAACA
AAGCAAAACTCAACTTCCCTGAAAATGTTAGGCTCAAGGAACAACCGAAC
CATTTGAACATGCTACATTCTAATTTGTCACATCAATCTAGAACTGATCC
AATGGTTCACAATGAAGTTCTTCATACTTTGCAAGGTTCAAACAAGTATT
ATGATTACTTTAATGGTCAAAACTTTCCTGTGGCTTCACTTCAAACTTCA
ATGTCACCGTCAGCTTCTTATTCTTCTTCTTCTTCTACGACTACTTTTGC
TTCTTCTTTTAGTTCTCCTCAGACAACTTCTTCAATTCCTTCAGGTTATT
CTACCCAGTTGCCAGCATGGTCTTCTGGATATAGCTCCTCTCCCTCAGGG
TGA
>MaERF090
ATGGGTGAGTGTGAGATATCTCTACCTAACTCACAGAAAGAACCTAAAAC
TTCATCTTCGGAGCCTCATTTATCCGACCCAAATGAGAAACGGTCCAAAC
GACCCAGAGAGAGCAACCACCCGGTTTACAGAGGAGTCCGAATGCGAGCA
TGGGGTAAATGGGTATCCGAAATCCGCGAACCGAGAAAGAAGAATCGGAT
CTGGCTAGGAACGTTCGCCACGCCGGAGATGGCTGCTAGAGCCCATGACG
TGGCAGCTCTCGCCATCAAAGGCAACTCCGCCATACTCAACTTCCCGGAA
CTCGCCGCTTCAATGCCCCGACCCGATTCAAACTCTCCCCGTGATGTTCA
AGCTGCCGCTGTTAAAGCCGCTGCCATGGAAGTTCCCGATCAACAAACAT
CATCATCATCTTCGCCGTCTTCATCTTCTTGTTCGTTGGCGGTTTCTTCC
TCCGACGAACCTTCAACACCAGATGAACTTGGAGAAATAGTGGAACTACC
AGCATTGGGAACAAGTTTTGAATTACCCGACCCGTGTAATATGGTATTTT
CCGACCCGATTGATGGATCATGGCTTTATTCACACTACTGTTGGTATAAT
AACAGCATTTACGATGAAAGGGATTATTATTTTATGGAGGATCAGATTTC
AATGCAGAAGCAGCAACAAGACAGCTCTGAAAGTATTACGACTAGTATGA
TACTTTGTGGTTTTGAGGGTACTTCTTCTTTGTGGCAACATTAA
>MaERF091
ATGGTAAGCTTAAGAAGGCGCAGACTTTTGGGACTATGCTCAGGAAATAA
TTCATTTGTCACTCCACTTCCTCTATACTGTGAGAATCTAGCTCGGTATG
AAAATTCAAGTCAGAATGCTAATCTTAAGAGCGGACAATCTGTTGTTTCG
GATATCGCAAGCATCCGAGACAGTGTAGGAACACAGGATTCCCAGAACAC
TGCTGTAAAAGATGAATCAGGATCATCAAATGTGTCTGGTTCTAGCCTGT
CTAAAGAGCAACCTAGTCAACAAAGCATAGGTCCTCCTGTTAAACGTAGA

AAGCGACATTCAAGAAAACCTAGAGAGAATCAGGAAACATGCTTAATGAG
AGGGGTGTATTTCAAAAATATGAAATGGCAGGCCGCGATAAAAGTGGACA
AGAAGCAGATCCACCTAGGGACTGTTGCGTCGCAGGAAGAAGCTGCCCGC
TTGTATGACAGAGCTGCTTTTATGTGTGGGAGAGAGCCCAATTTTGAGCT
TTCTGAAGAAGAAAAGCGTGAACTGAGTAAGTTCAAATGGGAAGAATTCT
TGGCCTTGACTCGAGAGACAATAACCTGTAAAAAACACAAGAGAAGTCAT
GGTCTGGGACCGGTTAATATGGTCGATGAGCCTTCATTGCGCAGAGGTGA
TTGTGACAGTAAGCAAGGAGTTACTGACTTCTCTGTCAACGGTGAACCAG
AGCAAGAAACAACTGGCTCTAGAGATATATGA
>MaERF092
ATGGTTCGAGTGAAAGGTAGAGATTATATGCCAAGGAAGAAAATCGTGAA
TTTGCCAGTGATTACTCGTTACGGCGGTGGATCTACGGTGGCGAAAAAAG
CAGAATCACCAGAACCGATGAAACCCGCGGTGGCTGCGGCGGTGAAGGAA
ATCCGTTACCGCGGCGTGAGGAAGCGACCATGGGGTCGATTCGCCGCCGA
AATTCGAGATCCATGGAAGAAAACTCGTGTATGGTTAGGAACTTATGATA
CCGCGGAACAAGCGGCTCAGGCTTATGATACTGCTGCTATCAAATTCCGT
GGGTCCAAAGCCAAAACTAATTTCGCTATCCCTGAACATATTATTGCGGC
CGGGGTAGTTGTTACCCCGGCCGCTATTCAGAATCCTCGGCCTCCGGTTG
TTACTGATAACCGTGGGGCTGCGGTTTCGAAGTTTATTGAGCCTGTTGCT
GAATTGATTAATTGGCCTACTTCTAGTGGTATGAGTAGTACTGTTGAGTC
TTTTAGTGGACCTAGGGTTCATGTTGTTGGTGGTTCTTCTTCATCTGCTG
CTTCCAGGGTTCATGCTGCTGGTGCTGCTGTTGTTGCTCCTAGACCTCTT
GCTGCTGTTGGTGCTGGTGGAGATTTTCACAGTGATTGTGATTCATCTTC
ATCAGTTGTTGATGATGATGAAGATTGTGTTATTCTAACATCTTCTGCAT
CAGTTCAGAGGCCGCAACCACAGCCACAGCCACAACTGCTTGATATTGAT
CTGAATTTCCCCCCACCATTGGATGATGATGAACTGATCCGTGTCACCAC
CCTCTGTCTTTGA
>MaERF093
ATGGGTGCTCCTGTTTATGATCAAAGTTTGAAGGTTTCTATTGTGCCAAT
GGAATCAAGTAAAAAGAGGAAAACTAGGAGTAGAGGAAAAGGGACTAAAT
CTGTGGCTGAGATTCTAGCAAAGTGGAAGGAATACAATGAGAAACTATAT
AAAGATGATGGTAAACCGAAGCGTAAAGCACCAGCTAAAGGTTCAAAGAA
AGGGTGTATGAAAGGTAAAGGAGGACCTCAAAACTCGGAGAATAAGTATC
GAGGTGTTAGACAGAGGACTTGGGGGAAATGGGTTGCTGAAATTAGGGAA
CCAAATAGAGGGAGTAGACTTTGGTTAGGTACTTTTCCGACAGCTCAGGA
GGCTGCTCTTGCTTATGATTATGCTGCTAGAGCTATGTATGGTCCTTCTG
CGCGACTTAACTTCCCGGATATCGCGGATTATAGTTCTATTCAGGAATAT
TTGAAGGATTCTTCATCAGCTGCTGCTGCTGCTAGCTGTTGTTCTTCAGT
GGCAACAACTCCTGCAACATCTGAGACAACAACTGTATCTGGTCATTCTG
AGGTTTGTGCTGCTGAGGATGTTAAGGAGATACCTAAGTTCCCTCTCAAT
ATGAACAATACTGTTGATGTTTGTCAGAAGGGTTATTATGAAGCTTCATC
TCCAACAAGTAGAATGAAGCTTGAGCCGAAGGATGAGCCTGTTGATATCA
TAGGCCTTGGTGGTGGTGAAATCCAAGATGCAAATTCAGAAGGAACACAA
ACACATGATGCTGTGCAGGTTGGAGAGGGTGTGAGTAATGACCAGATGGA

TTTTTCATGGATGGATAACTTTGACATCACTGATGACTACTTGAAGAGTT
TTTCGATGGATGAGTTCTTTCATGTGGATGATTTCTTTCAGGTGGATGAG
CTTCTGGGGCATATAGATAATAATCCAATTGATGAATCTGGGGTGATGCA
AAGTTTGGATTTTGGACAAATGGGTCTTCCTGAAGAGAGTAATAATCCTC
AGGCTGGGACTACTTCAAGCTTCTTTTATGAATTGGAAAATCCTGATGCT
ATGCTGTTGGGAAGTTTGCCTCACATGGAACACACAGCATCAGGTGTTGA
TTATGGATTACCTCTGAACTTCAATGGTGGAGTAGAAGATACACCGTTTC
TTGATTTGGATTATGATCTAAATCACGATTCAGGAGGAATGAAAGGAAGG
AAGAATGATTAG
>MaERF094
ATGGATTTACATTCACCTCTTCATCATGTTCAAGAAACCAAACCCACCGC
CAACACCGTTGTTACCACCACTCCAAGTGAAACATCTAACAACAGCACTG
ATAACAGCAGCAACAGCATTAAAAATAATAACCGTAAATCCAAAGGTAAA
GGTGGACCTGATAACAACAAATTCAGATACCGTGGTGTAAGACAACGTAG
TTGGGGTAAATGGGTTGCTGAAATTCGTGAACCACGTAAACGTACTCGTA
AATGGCTTGGTACTTTTTCAACTGCTGAAGATGCTGCTAAAGCTTATGAT
CGTGCTGCTATTATTCTCTATGGTTCTAGGGCTCAGCTTAATCTTCAACC
TTCAGCTTCATCTTCTTCCTCTCAAAACTCTTCATCTTCTTCTCGTAACT
CTTCTTCTTCCAATTCCAATAATACCCTTCGTCCTTTACTTCCTCGTCCT
TCTGGTTTCTCTCTTTACAATAATCTAGTTCCTTTTGGTGTTTACAATAA
TTTTCATCATCATCATCAACCAGTTTTCTATAATAACAATAATAGTTTAG
TTCATTTTCATCATAATCCTCATCATGAAATGGTGCAAGTTCAAGTGCAA
CAACATCAACAATATCATCAACATCAAGATTTTGAACATGCAAGTGGTGA
TTCTGTTAAATCAATTACCTCGTATGGTCAGAATATTCACGATCAAGAGC
AACATGCAAAACATGTTTTGGATCAACAACATGCACAACATGTTATGAAT
CAGCAACAACAACAGATGAATATTCAGAATTGTGTGGGGTCATCAAGTTT
TGGTTGTTCTCAAAATAATAATGATATTGATGGAACAGTACTGGATCTGG
ATCCAGTTGGTGTTTGTGTTGGTTCTCCTAATTCTATGTGGCCAGCTTTG
ACAAGTGAGGAAGATTATACAACTAGTTTATGGGATTATAATGATCCTTT
CTTCTTTGATTTTTGA
>MaERF095
ATGTCTCTACAGAAGCTCAAGCAACTAAAACTCTCAACACCAAGTTCAAT
TTTTGAAGACAACCCATCAATCAAAACAAAGAGAAAACCCAAAAGAAAGC
TTGTTCGAGTCATAATCACAGACCACGACGCCACAGATTCCGATTCCTCC
GGCGACGAAGAGAGAAAGAATAACAACCAAACAACAAAACGAAGAAAACC
CAAGAGAGAAATCCTTCATATCAACATGCACCTTCCCGTACCCAATTCTT
CTCTCTCATTTTCTTCTCCTTCATCTTATTCTTCACTTGCTTCGCCGGAG
AAAACCAACAAAAAGTTCAAAAGAGTGAGTAGACCCAAAAACCCACCAAC
CTCCGCCGTCACGCGCCGCCACATTAAGTTCAGAGGAGTGAGACAACGGC
CGTGGGGAAAATGGGCTGCTGAGATCCGTGACCCGACCCGAAGAAAACGT
CTCTGGCTTGGAACATTCAGCACGGCGGAGGAAGCCGCCGTTGAGTATGA
CAGAGTTGCGGTGATGATACACGGTCCTAACGCCGTAACAAATTTTCCAA
TAACGCCGGTGAAGATGGAAACTGAAACTGAAGTGCTTACGGCGGTTAAC
GGTAGAAGTGACGGCGGAGGTGGTTACTCCGACGCTTTAGCATCCTCAAC

GTCTGTTTCAACTTGCGACGGCGGAGGTGGCTATACCGACGCTTTAGCGT
CGCCAACGTCTGTTTTAACGTACGACTGTGATTCGACGCCGTTCGACGGT
TTCCGTTACGTCGACGTGGACGCTTTTGGGTTTCACATCGACGCGCCGTT
AAGTTTACCGGAAGTTAACGTTACGCTGACGTGTCATCAGAAGCTGGAAA
AAGAAAAGTTTGAAGAGTTTGATCTTGACGAGTTTATGACGTGGCCGTAC
TGA
>MaERF096
ATGGGTCGTGGAAACGGCACCACCGCAACAACTGCGGCTGAACCTGGTTC
AAACCCGGTTTTTTTCAAAGAGCCGAGATACAGAGGCGTTAGAAAAAGAC
CGTGGGGCCGGTTCGCCGCCGAGATCAGAGACCCTTTGAAGAAAGCAAGA
GTTTGGCTCGGAACGTTCGACACGGCGGAACAAGCGGCGCGTGCATACGA
CACCGCCGCTAGAAACCTCCGGGGACCAAAAGCCAAGACGAATTTTCCTC
TTCAACAGCCTTTTTACCATAATCTTGACGCCGGAGATCCGTTTTCCGAT
CAACGTTTCTTCACCGGCGCCGGTGCCGGAGTTGTTGCCGGTGAGTATCA
AGATCATCGGAGACCTACTTCATCTGGCATGAGCAGCACCGTTGAGTCTT
TCAGCGGTCCACGTCCAGTTGCTCCTCCGGCGCCGCCGTCTGTAGTTGCC
GGAAGAAGGTACCCACGTACACCACCGGTTGCTCCAGAGGATTGCCGTAG
TGACTGTGATTCATCATCGTCGGTGGTTGATGACGGCGACAACGACAACG
CGGCTTCGTCGGTGATGCTTTCGTTCAAACGCGCTCCGCTGCCGTTTGAT
CTCAACGCGCCGCCGTTGGAGGATGCTGACGTGGCAGGTGGTGAGGATCT
GCACTGCACCGTTCTATGCCTCTGA
>MaERF097
ATGGATTTCTTCAATAATTCACAAGATCCATTCAATGGTGAGTTAATGGA
AGTTCTTGAACCTTTTATGAAGAGTTCTTCCACTACTCCCTCATCACCCC
CTTCTTTAAATTCATACCTCCCTTCAACTTCATCTTCATCACCTTTTTAC
CCTTCTACCCCTTCTTTCTTCCAACCTTCTTCTTTTTCTCCTCAACCTTC
ATCTTCTTTTCCTTCACAGCCCAATTTCTACACAGAAAATGGTTATGGTT
CATCAATGATGAGTTACCAATTTCCTCCTTCCTTAGGTGATTCATCATCA
AATAGCCAAAATAATTTTATTGGCTTTGAACAACAACCACAACCAAATTC
TTTTATTGGGCTAAACAACTTAACCCCATCTCAAATTAACCAGATCCAAG
CCCAGATCCAGTTTCAGGCCCAACAACAACAAAACAACAGCACAAGCTTG
AACTTTCTTGGGCCTAAGCCCATCCCAATGAAACAGCCAGGTGTACCTCC
AAAGCCCACAAAGCTTTATAGAGGTGTTAGGCAAAGACATTGGGGGAAAT
GGGTAGCTGAGATAAGACTACCTAAGAATAGGACAAGGCTTTGGCTTGGT
ACTTTTGATACTGCTGAAGAAGCTGCTTTAGCTTATGATAGAGCAGCTTA
TAGATTAAGAGGTGATTTTGCTAGACTTAATTTCCCAAACATGAAAGACC
AACAAGGTATGTTTGGTGAGTTTAAGGTTTTGCATTCATCTATTGATGCT
AAACTTGATGCTATTTGTGAGAGTTTAGGTAATAGTAGTGATGTCAAGAA
ACAAGGGAAGGTTGAGAAAGGTTCTAAGAAGAAGGTTTTGAAGAAAGAGG
TTCAATCTCCTCAACCATTGATTGTTGTTGAGAATAATGATGATGATAAC
AACAACAACAACAACAAGGTTGTTATTGAAGGTTCTTCTTTGTCTTCACC
TAGTGAAGGTTCTGATGATTCTTCACCACTTTCAGATCTTACTTTTGGTG
AATTTGCTGAGCCACAGTGGGATAATGGTTGTGAACAGTTTATGAATCTG
CAGAAGTTTCCTTCTTATGAGATTGATTGGGCTTCTCTTTGA

>MaERF098
ATGGGTCCAGCAAGTCAGAAGCATAATTCATCTTATGTCTCGAAAAGCAC
AAGGAGGTTGAGAATCATCTATGATGATCCTGATGCAACAGATTCATCAT
CTGATGAATCAAATCATTATGTCCAGGAACCAGGGAAGAGGAAGAGAGTT
GTGCTTGAACTTGCAATCCCTAATTCTGAGGAAACAGTGACTGGAAAGAA
TAAGAAGAGACGTGGAAAACCAACACCTACAGTCAGACAACAATCTTGCA
AATATAGAGGTGTTCGAAGGAGGAAATGGGGTAAATTTGCTGCTGAGATC
CGTAATCCGTTTACAGGTAAACGGAAATGGTTGGGTACTTTCGACACGGC
TGAAGATGCTTCAAAGGCATATCAAGATCAAAGACTTGAATTTACAGCCA
GGGCTACGCGTTTGTCTAATGAGAATAGGAACAAAAATTTCAAAAATCGT
GTTACGATTAAGACTACAGTTGATGCTTTAAAGCCATCTTCGCCCGATCT
GGTTAATTGTGTTCCTAAATTGATTGAGAGTGGTAAGATTTCAGGTAATG
AAGCTATTGAGACTAATTCTTTTCAAGTGGTGCAAAAACAGGATGTAGTT
GGGAGTCCGGATTTGTCTTGGTTGGATGTAGATTGTTTAACATTTGATGG
TTCGGGGCAAGGCTTGGTTCATCTTGGTTTCCGTAATGACCTTCAGGTTT
CTGATTTTGATAAGAATGGACCAATTGCGATCGATCTTCCATATCTTAAT
TTGGGTGACTTTGAGGCAGGAAATATGATTTTTAATGATGTTGGTGCTGA
ATCTGTTGATGAGGATGAGATTGCTAGTTGGATTGAAGAACCCTCGGACG
CACCAGGTCCATAA
>MaERF099
ATGGTTAAGACAGAGATTAACAAGATCAACAAAGAAAAATCAAAGCCAAT
GCAACAACAAACTTTATCATCAACAGCAGCATCATCAAATGAGAAGAAGA
AAAAATACAAAGGAGTGAGAATGAGGAGTTGGGGTTCTTGGGTATCAGAA
ATTAGAGCTCCAAATCAAAAAACAAGAATATGGTTAGGTTCATATTCAAC
TGCAGAAGCAGCTGCTAGAGCCTATGATGCTGCACTTTTATGCCTTAAAG
GTTCCTCTGCAACAAATCTTAACTTCCCTTTAGAATGTTCTTCACATTTC
ATTCCTCAAGAAAACATTGCTATGTCACCTAAATCCATCCAAAGAGTAGC
TGCTGCTGCTGCTGCTGCTGCTGCTGCTAGTAACAATGATATTGCTATTA
GCACCCCACCAACTTCTCCTCCTAATCTTGCTTCAACAACTACCTCATCT
TCAATAGTTTCATCTCCATCAATGTCATCTTCTCCTTCTGATCAAATTGA
TGATGATGTTTCACTGTTTTCATCTTTTGGGGCCTACACTACTAGCTGTG
ATGATCATTATCAACCAAATGAATCAATGGCTATGATGGATTCTTGGTAT
GGATTTGATGGTTTACAATCACCAAAACATGTTGTTGATCAAATGCTAAG
TGGTGCGTTGTTTGATATTGATTCAACTCAAGTACTTCTTGATGATTGGT
ATGAAGAAGGTGACATTAGTTTGTGGAGCTTCTACTAA
>MaERF100
ATGTCCTTGTTAACGGTGGCGCATCAAAGAGGTTCAGGTGAGTTCACCAA
ATTCACGGTGAAGCGCGACGGCGATGATCACGACGATGGTGGAAATTCTA
GTGCATCACAATCATTAGATTTCAACCAAATGCATGGTGATAGAGAGTTC
ACAGAAATGGTTTCAGCTCTTACACATGTTGCATCATCAGGGTCTAATCA
AATGAGTAGTAATGAATGGATTCAAAGAAGTGGTTTTCCTTTGATATCAA
GTTTTGGAAATGCTTCATCTTCTTCTTCTTCTTATTCTTCAAGTTCTTCT
AGCTTTGTTTTACCCTCTGGATCATCATGGAGTGGTCAAAAAAGAGGGCG
TGATGAAGAAAGTAGTGGTTCTAATCAAATTTTACAACAATCTATTCCTA

GACTCTTTAGAACTGTTATGGTACCTTCTCAAGGACAAGGAGAATCATTA
TCATCATCATCATCAGTGACTGAAGAAGCTCGCACAAATACTACCATAGC
CACCTCCTCCTCAGCGTCGGCCGCATCATTGGAAGAGTCCGGCGAGCAGA
GGAGAAGATACAGAGGAGTAAGACAGAGACCATGGGGAAAATGGGCAGCA
GAGATACGAGATCCACACAAAGCACAAAGAGTATGGCTTGGAACATTTGA
TACAGCAGAATCTGCAGCAAGAGCATATGATGAAGCTGCATTGAGATTCA
GAGGAAGCAGAGCAAAACTCAACTTCCCTGAAAATGTTAGATCAATTATG
CCGCCACCGCCACCGCAGCCACAATCACTATCACAGCTTCAATCTTTTCC
GGCTGCCGCGGTTCCCTCTCGAACGCCGCAGCCGCCGGTTCAAGGAGCCT
CTGATTTGAGAGACTATTTGGAATATTCTCATATTCTTCAAAGTTCTGGT
GACTTTCAACTACAACAAATGCAGCAACAACAACAACAACAATTACAAGC
TTCTAATTTGGTTCAACAATGGTATTATAATGCACAATTGGCAGCACTTC
AATCACATTCATTGTTATCTTCATCATCATCGCTCTCTATGCCTTCTAAT
ATGTTACCATCAACACCTTCATTTTCACCCTCCACTCAACTTTCATCCAC
CTCTGCTTCATTTCCCCTGTTTTCTAGTCAACAAATGGGATTTTTCCGGC
CGCCAGAAAACCGCCCGCCTAGTGGTGGTCATGGCGGCGGAACAGAGTTT
CCGCCGTCAACATGGTCAAATACTGATGGCCATCCACCACCACCTTATGG
TTGA
