# Supplementary material for: Genome-Wide Identification of ERF Transcription Factor Family and Functional Analysis of the Drought Stress-Responsive Genes in Melilotus albus
Source: Int J Mol Sci. 2022 Oct 10;23(19):12023. doi: 10.3390/ijms231912023 (PMC9570465; doi:10.3390/ijms231912023)
Supplement: Supplementary file 1 [file ijms-23-12023-s001.zip › Table S2.pdf]

Gene name	Gene ID	Group	Proteinlength(aa)	Molecular weight(Da)	pI	Protein GRAVY	Subcellular localization
Malbus0101835.1	MaERF006	I	408	45915.75	6.03	-0.549	nucl
Malbus0202543.1	MaERF026	I	358	39282.61	6.41	-0.659	nucl
Malbus0401158.1	MaERF041	I	432	48782.37	5.96	-1.023	nucl
Malbus0600266.1	MaERF069	I	320	35619.04	8.72	-0.504	nucl
Malbus0704595.1	MaERF088	I	296	33682.02	6.37	-0.538	nucl
Malbus0802502.1	MaERF097	I	363	40500.87	5.18	-0.754	nucl
Malbus0105752.1	MaERF014	II	175	20122.54	8.73	-1.005	nucl
Malbus0200993.1	MaERF022	II	239	26887.55	4.9	-0.637	nucl
Malbus0201184.1	MaERF023	II	155	17821.81	7.8	-1.045	nucl
Malbus0202615.1	MaERF027	II	218	23944.53	5.41	-0.465	nucl
Malbus0402906.1	MaERF044	II	211	23425.91	4.68	-0.509	nucl
Malbus0402907.1	MaERF045	II	197	21511.88	4.67	-0.355	cyto
Malbus0402909.1	MaERF046	II	215	23570.07	4.69	-0.433	nucl
Malbus0600306.1	MaERF070	II	164	18537.77	5.79	-0.538	nucl
Malbus0601340.1	MaERF072	II	170	18875.07	9.64	-0.909	nucl
Malbus0802645.1	MaERF099	II	245	26642.67	5.07	-0.391	nucl
Malbus0101988.1	MaERF008	III	207	23440.21	5.3	-0.654	nucl
Malbus0101990.1	MaERF009	III	189	20384.56	6.06	-0.655	nucl
Malbus0105251.1	MaERF013	III	260	28657.88	5.65	-0.608	nucl
Malbus0200681.1	MaERF019	III	181	20225.87	7.91	-0.519	cyto
Malbus0301440.1	MaERF31	III	245	27226.53	4.97	-0.9	nucl
Malbus0304052.1	MaERF038	III	188	20756.32	4.8	-0.412	nucl
Malbus0304337.1	MaERF039	III	206	22574.83	4.62	-0.529	nucl
Malbus0400313.1	MaERF040	III	206	23263.32	5.9	-0.486	nucl
Malbus0500043.1	MaERF052	III	220	24398.69	5.78	-0.851	nucl
Malbus0503845.1	MaERF061	III	257	28132.32	5.62	-0.556	nucl
Malbus0503955.1	MaERF062	III	238	26986.48	6.11	-0.532	nucl
Malbus0503956.1	MaERF063	III	173	18886	4.94	-0.469	nucl
Malbus0600161.1	MaERF067	III	218	24753.66	6.85	-0.592	nucl
Malbus0600316.1	MaERF071	III	186	20770.17	5.29	-0.562	nucl
Malbus0601550.1	MaERF074	III	192	21553.94	5.15	-0.742	nucl
Malbus0602944.1	MaERF075	III	230	26516.72	6.02	-0.93	nucl
Malbus0602948.1	MaERF076	III	257	28961.36	6.97	-0.939	cyto
Malbus0602949.1	MaERF077	III	308	34905.8	7.08	-0.405	nucl
Malbus0602959.1	MaERF078	III	261	29510.64	6.53	-0.305	cyto
Malbus0705194.1	MaERF090	III	247	27460.45	4.95	-0.669	nucl
Malbus0105967.1	MaERF015	IV	269	29626.95	4.99	-0.591	nucl
Malbus0200523.1	MaERF018	IV	247	27855.22	5.7	-0.801	nucl
Malbus0302349.1	MaERF032	IV	506	56147.54	6.69	-0.904	nucl
Malbus0303553.1	MaERF036	IV	289	32661.44	6.08	-0.828	nucl
Malbus0401244.1	MaERF42	IV	193	20927.46	9.21	-0.701	nucl
Malbus0503598.1	MaERF057	IV	229	25425.68	8.42	-0.441	nucl
Malbus0701600.1	MaERF080	IV	195	21357.79	8.39	-0.792	nucl
Malbus0801067.1	MaERF093	IV	403	44260.05	4.86	-0.693	nucl
Malbus0801091.1	MaERF094	IV	321	36300.42	7.07	-0.987	nucl
Malbus0200076.1	MaERF016	V	176	19880.65	9.32	-0.643	nucl
Malbus0200876.1	MaERF020	V	203	22786.49	6.45	-0.713	nucl
Malbus0200931.1	MaERF021	V	189	21897.79	8.44	-0.746	cyto
Malbus0203970.1	MaERF028	V	195	21937.48	5.96	-0.662	nucl
Malbus0303182.1	MaERF034	V	268	29980.39	5.7	-0.612	nucl
Malbus0504519.1	MaERF064	V	216	24063.15	8.66	-0.657	nucl
Malbus0603750.1	MaERF079	V	309	34573.04	6.34	-0.799	nucl
Malbus0704315.1	MaERF087	V	238	27271.42	6.9	-0.807	nucl
Malbus0201834.1	MaERF025	VI	355	39747.2	4.77	-0.619	nucl
Malbus0600255.1	MaERF068	VI	307	34304.94	5.04	-0.732	nucl
Malbus0703361.1	MaERF084	VI	227	25461.29	5.1	-0.666	cyto
Malbus0703362.1	MaERF085	VI	216	24637.6	5.49	-0.715	cyto
Malbus0703364.1	MaERF086	VI	218	24195.72	4.73	-0.652	nucl
Malbus0801550.1	MaERF095	VI	300	33615.87	9.19	-0.698	nucl
Malbus0101711.1	MaERF005	VII	324	36736.64	5.82	-0.964	cyto
Malbus0200170.1	MaERF017	VII	377	41727.7	4.75	-0.775	nucl
Malbus0403691.1	MaERF047	VII	265	29226.96	4.95	-0.474	nucl
Malbus0500879.1	MaERF055	VII	385	42401.63	4.81	-0.705	nucl
Malbus0101031.1	MaERF003	VIII	144	16465.41	8.95	-1.074	nucl

Malbus0203984.1	MaERF029	VIII	205	22599.42	10.19	-0.817	nucl
Malbus0303096.1	MaERF033	VIII	271	30009.61	4.88	-0.836	nucl
Malbus0401475.1	MaERF043	VIII	210	22952.87	9.55	-0.447	cyto
Malbus0404045.1	MaERF048	VIII	207	22643.18	7.8	-0.615	nucl
Malbus0404046.1	MaERF049	VIII	169	18384.99	9.99	-0.42	nucl
Malbus0404323.1	MaERF050	VIII	291	32772.48	5.78	-0.9	nucl
Malbus0503004.1	MaERF056	VIII	296	33419.88	6.46	-0.761	nucl
Malbus0504791.1	MaERF065	VIII	344	38683.32	5.29	-0.735	nucl
Malbus0600078.1	MaERF066	VIII	352	38845.64	7.67	-0.748	nucl
Malbus0702998.1	MaERF083	VIII	232	25303.24	9.38	-0.725	nucl
Malbus0800991.1	MaERF092	VIII	270	28849.85	9.13	-0.213	nucl
Malbus0801834.1	MaERF096	VIII	224	24084.76	6.83	-0.593	nucl
Malbus0101030.1	MaERF002	IX	136	14834.21	5.9	-0.804	nucl
Malbus0101032.1	MaERF004	IX	212	23845.25	4.73	-0.769	nucl
Malbus0101843.1	MaERF007	IX	188	21540.95	8.41	-0.866	nucl
Malbus0104017.1	MaERF011	IX	225	25257.63	9.17	-0.615	nucl
Malbus0104722.1	MaERF012	IX	216	24782.83	6.85	-0.787	nucl
Malbus0303509.1	MaERF035	IX	417	46531.95	8.76	-0.818	nucl
Malbus0303828.1	MaERF037	IX	141	16058.95	5.45	-0.633	nucl
Malbus0404525.1	MaERF051	IX	292	33138.34	5.53	-0.618	nucl
Malbus0500096.1	MaERF053	IX	185	20427.92	9.07	-0.589	nucl
Malbus0500098.1	MaERF054	IX	198	21948.41	9.33	-0.601	nucl
Malbus0503830.1	MaERF058	IX	267	28943.57	7.74	-0.413	nucl
Malbus0503833.1	MaERF059	IX	310	34998.42	5.5	-0.599	nucl
Malbus0503834.1	MaERF060	IX	311	35212.71	5.58	-0.584	nucl
Malbus0601540.1	MaERF073	IX	265	29314.89	6.22	-0.516	nucl
Malbus0701909.1	MaERF081	IX	249	28048.14	6.37	-0.679	nucl
Malbus0701917.1	MaERF082	IX	259	29056.49	6.04	-0.576	nucl
Malbus0705394.1	MaERF091	IX	243	27300.6	9.12	-0.873	cyto
Malbus0102177.1	MaERF010	X	368	39788.53	6.8	-0.712	nucl
Malbus0201381.1	MaERF024	X	263	29801.36	6.08	-1.078	nucl
Malbus0300584.1	MaERF030	X	171	19032.18	8.69	-0.707	nucl
Malbus0704687.1	MaERF089	X	300	33405.68	6.6	-0.824	nucl
Malbus0803481.1	MaERF100	X	417	45288.42	6.51	-0.732	nucl
Malbus0100331.1	MaERF001	VI-L	371	41535.06	5.15	-0.659	nucl
Malbus0802632.1	MaERF098	VI-L	287	32025.66	6.86	-0.789	nucl
