# Supplementary material for: Genome-Wide Identification of ERF Transcription Factor Family and Functional Analysis of the Drought Stress-Responsive Genes in Melilotus albus
Source: Int J Mol Sci. 2022 Oct 10;23(19):12023. doi: 10.3390/ijms231912023 (PMC9570465; doi:10.3390/ijms231912023)
Supplement: Supplementary file 1 [file ijms-23-12023-s001.zip › Table S3.pdf]

Gene name	Forward primer sequence	Reverse primer sequence
MaERF004	ATTCCAAACTCTTCCACTTCAG	AAAGTTCCTAACCAAACCCTC
MaERF008	TGAGAATAATGAAGGGAGGAAGGA	GTCAGCAACACCACAATCAC
MaERF010	CATCATCAGCAACAGAAGCA	GTCTCTGTCTCACTCCTCTG
MaERF012	GGTTAGGAACATTTGATAGTGCTG	AAAGCCACAACAGGAGAACAC
MaERF016	GTTTCTGAGATTCGTCATCCA	CAAGTGAGTGAAGGTGAAGG
MaERF017	GCAATTATCTCCGACTTCATCTC	AGCACTTGATCCACGAACAG
MaERF034	ACTCTTTCAACTTGTGCTACTC	CCATCATTCTTCGCCATATCC
MaERF037	GGTACATTTAACACTGCTGAGG	GAGGTTGAATTGGATGAAGAAGAG
MaERF054	GAAGGAGGATGATACCGAAGAC	GTTTCATAAGTCCCAAGCCAC
MaERF058	TTTAGACTCTATTCGCCGCC	GCCGTAAAGTACCATATCTTCAG
MaERF085	AGGATGCAATTTCTTTCCCGA	CCGCTGTTGTAAATGTTCCGA
β-tubulin	CCTTGGTGGTGGAACTGGT	GGAGATGGGAACACTGAGAAAG
