# Supplementary figures and images for: Genome-Wide Identification of ERF Transcription Factor Family and Functional Analysis of the Drought Stress-Responsive Genes in Melilotus albus
Source: Int J Mol Sci. 2022 Oct 10;23(19):12023. doi: 10.3390/ijms231912023 (PMC9570465; doi:10.3390/ijms231912023)

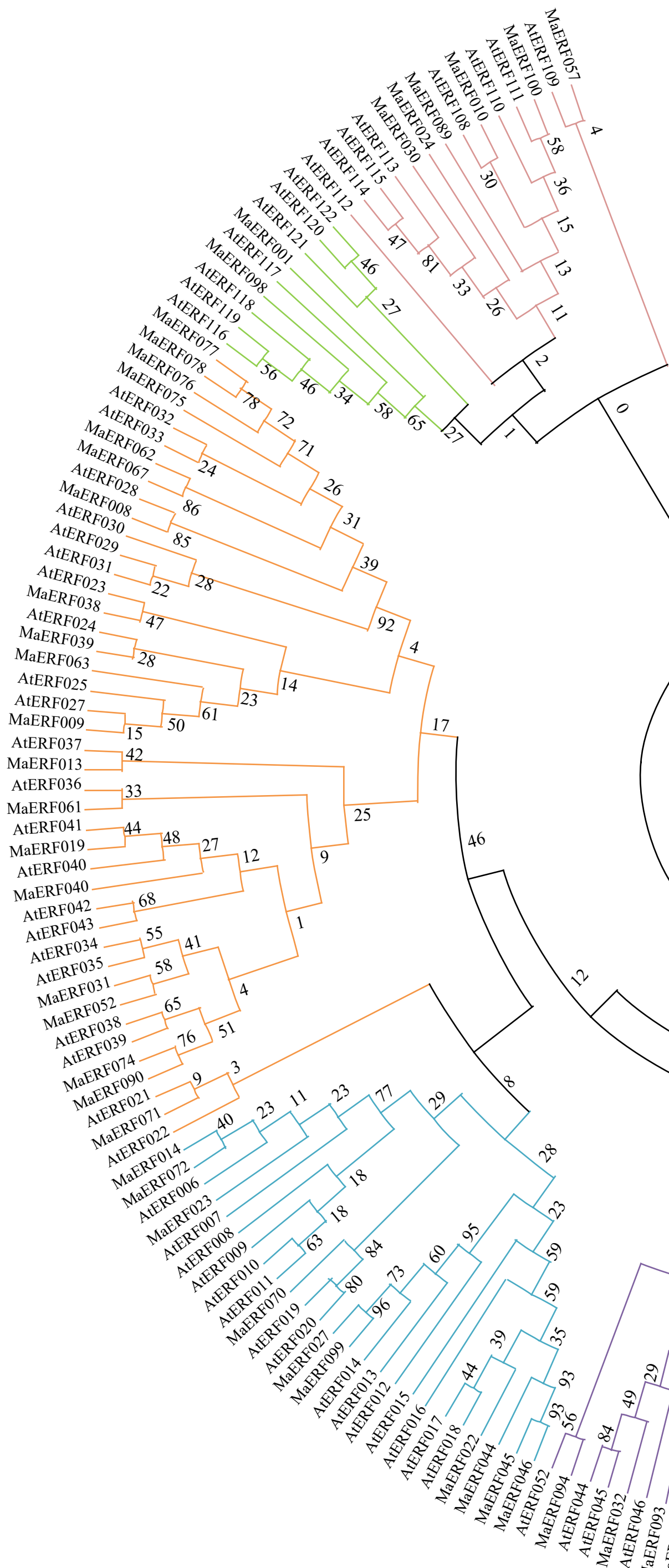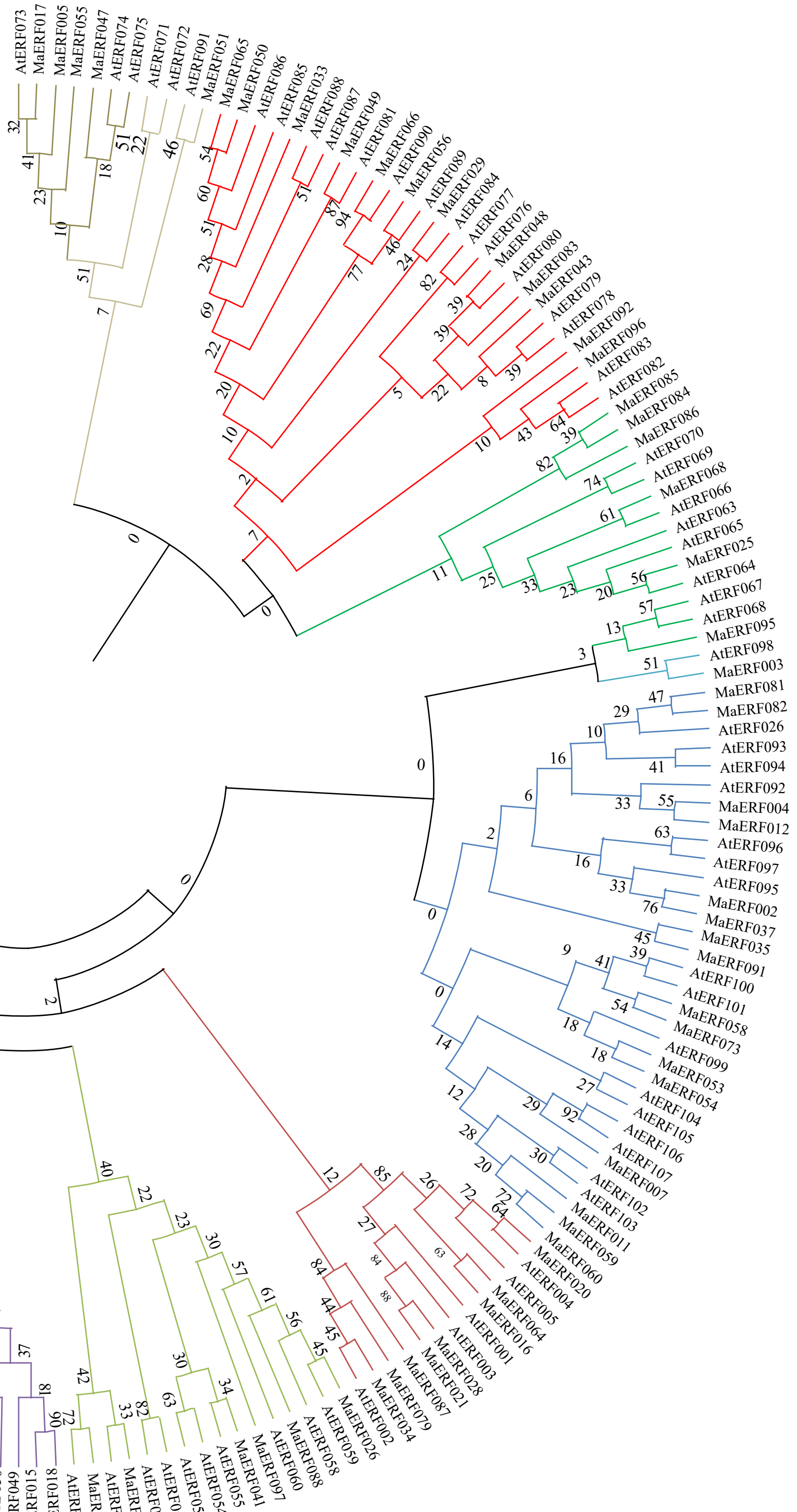

Supplement: Supplementary file 1 [file ijms-23-12023-s001.zip › Figure S1.pdf]
